# When Origin Matters: Properties of Mesenchymal Stromal Cells From Different Sources for Clinical Translation in Kidney Disease

**DOI:** 10.3389/fmed.2021.728496

**Published:** 2021-09-20

**Authors:** Sandra Calcat-i-Cervera, Clara Sanz-Nogués, Timothy O'Brien

**Affiliations:** Regenerative Medicine Institute (REMEDI), CÚRAM, Biomedical Science Building, National University of Ireland, Galway, Ireland

**Keywords:** mesenchymal stromal cells (MSCs), tissue source, good manufacturing practice (GMP), advanced therapy medicinal products (ATMPs), kidney disease, cell therapy, clinical application

## Abstract

Advanced therapy medicinal products (ATMPs) offer new prospects to improve the treatment of conditions with unmet medical needs. Kidney diseases are a current major health concern with an increasing global prevalence. Chronic renal failure appears after many years of impairment, which opens a temporary window to apply novel therapeutic approaches to delay or halt disease progression. The immunomodulatory, anti-inflammatory, and pro-regenerative properties of mesenchymal stromal cells (MSCs) have sparked interest for their use in cell-based regenerative therapies. Currently, several early-phase clinical trials have been completed and many are ongoing to explore MSC safety and efficacy in a wide range of nephropathies. However, one of the current roadblocks to the clinical translation of MSC therapies relates to the lack of standardization and harmonization of MSC manufacturing protocols, which currently hinders inter-study comparability. Studies have shown that cell culture processing variables can have significant effects on MSC phenotype and functionality, and these are highly variable across laboratories. In addition, heterogeneity within MSC populations is another obstacle. Furthermore, MSCs may be isolated from several sources which adds another variable to the comparative assessment of outcomes. There is now a growing body of literature highlighting unique and distinctive properties of MSCs according to the tissue origin, and that characteristics such as donor, age, sex and underlying medical conditions may alter the therapeutic effect of MSCs. These variables must be taken into consideration when developing a cell therapy product. Having an optimal scale-up strategy for MSC manufacturing is critical for ensuring product quality while minimizing costs and time of production, as well as avoiding potential risks. Ideally, optimal scale-up strategies must be carefully considered and identified during the early stages of development, as making changes later in the bioprocess workflow will require re-optimization and validation, which may have a significant long-term impact on the cost of the therapy. This article provides a summary of important cell culture processing variables to consider in the scale-up of MSC manufacturing as well as giving a comprehensive review of tissue of origin-specific biological characteristics of MSCs and their use in current clinical trials in a range of renal pathologies.

## Introduction

According to the World Health Organization 2019 Global Health Estimates, chronic diseases are one of the leading causes of mortality worldwide (1). Amongst them, chronic kidney disease accounts for 11–13% global prevalence (2, 3). Based on the course of the injury, kidney diseases and their spectrum of clinical manifestations are stratified into acute kidney injury (AKI), chronic kidney disease (CKD) and end-stage renal disease (ESRD) (4, 5). Persistent loss of kidney function over time leads to kidney failure and at that stage, the current standard of care includes renal-replacement therapies (RRT) (mainly hemodialysis and peritoneal dialysis), or organ replacement. Both strategies suffer significant drawbacks that underpin the need for new preventive and therapeutic approaches.

Cell-based regenerative therapies have the potential to change the paradigm of conventional clinical care. The use of complex biological entities such as cells to promote tissue regeneration and homeostasis, provides a therapeutic alternative to treat and even cure a wide range of diseases. The current cell-based clinical landscape in kidney disease uses hematopoietic stem cells (HSCs), mesenchymal stromal cells (MSCs), and a wide range of blood-derived cells, such as T cells, natural killer (NK) cells, and dendritic cells (6, 7). Notably, blood cell-based therapies using myeloid and T cells are gaining relevance as cellular immunotherapy products to regulate the immune response after procedures such as kidney transplantation (8, 9).

**Figure 1:**
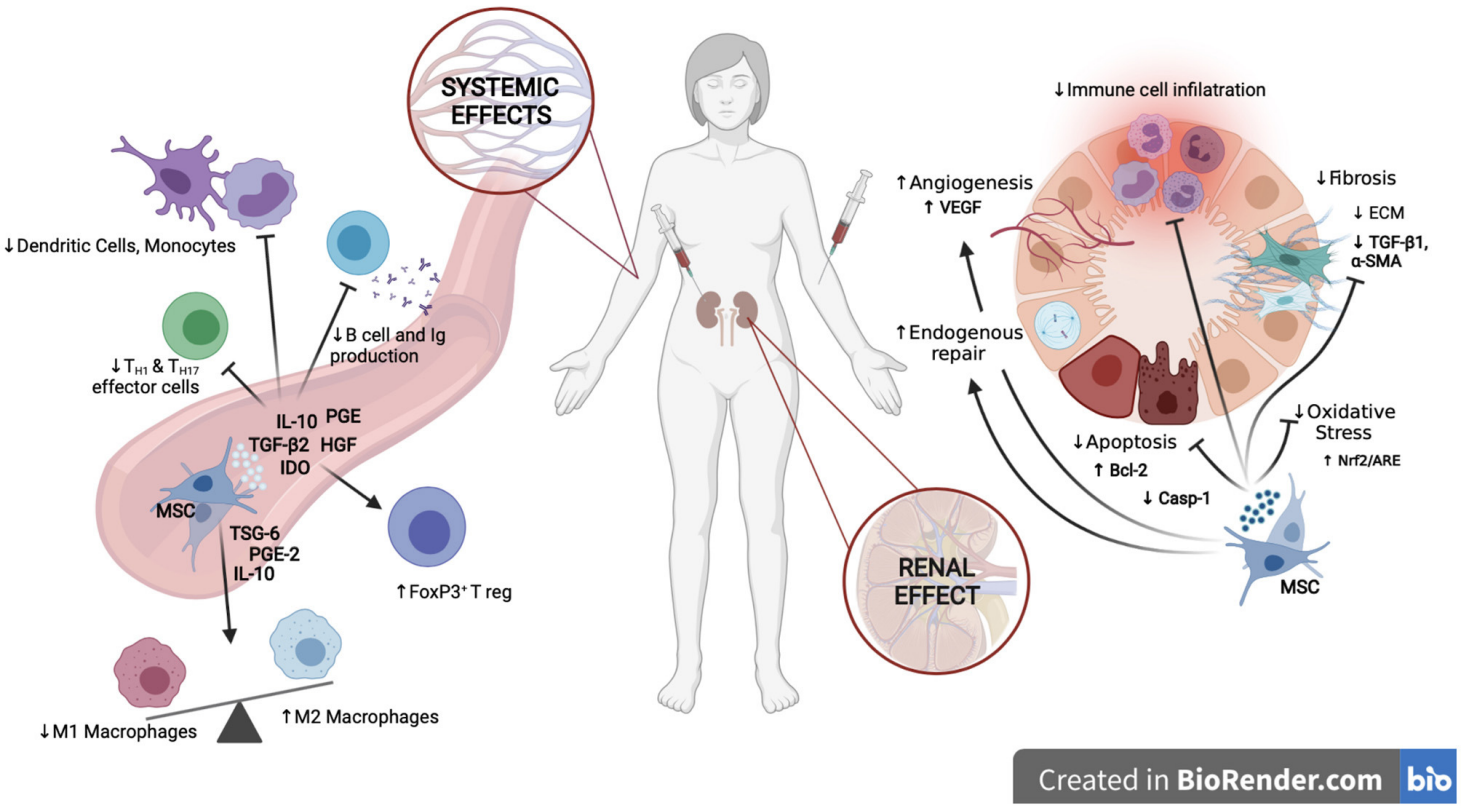
Mechanisms of action of MSCs in kidney disease. At the renal level, MSCs enhance endogenous mechanisms of repair, confer cytoprotection by dampening apoptosis and oxidative stress, promote vascular preservation and regeneration, diminish renal fibrosis and reduce infiltration of immune cells, creating an anti-inflammatory and pro-regenerative environment. At the systemic level, MSCs inhibit the pro-inflammatory activities of both, the innate and adaptative immune system, enhancing the expansion of tolerogenic T reg and M2 Macrophages while inhibiting M1 macrophages, monocytes, dendritic cells, and T and B lymphocytes. Created in BioRender.com.

On the other hand, MSCs, which are considered an advanced therapy medicinal product (ATMPs) under EU regulation, have been extensively investigated during the last decade due to their ability to inhibit inflammation and initiate tissue regeneration. The immunomodulatory and anti-inflammatory effects, *via* interactions with immune cells, together with paracrine secretions of anti-apoptotic, anti-fibrotic and matrix remodeling factors, are the main MSC-mediated mechanisms contributing to kidney protection and regeneration (10–12) (Figure 1). The effectiveness of MSCs in the treatment of a variety of nephropathies has been largely investigated in pre-clinical models, showing promising results (13). This has encouraged the translation of their use in clinical settings and currently, several early-phase clinical trials have been completed, and many are ongoing, to explore MSC safety and efficacy in renal transplantation, autoimmune diseases, and organ regeneration, especially in late-stage chronic kidney disease patients (Table 1). Nevertheless, the road to their routine use in the clinic is far from being a reality. Results in the clinical arena have highlighted the need for better defined therapeutic products. The intrinsic heterogeneity of MSCs in addition to efficacy and safety needs to be extensively investigated before they become a sustainable and affordable therapy (43–45).

**Table 1:** Summary of clinical trials in KD using MSC registered at ClinicalTrials.Gov. Search done on 23rd April 2021.

**NCT number**	**Status**	**Phases**	**Start date**	**Cell source**	**Donor source**	**Dose frequency (N)**	**Infusion route**	**Results**	**References**
**Acute Kidney Injury**									
NCT00733876	Completed	Phase 1	2008	BM-MSC	Allogeneic	2 × 10^6^ cells/kg body mass *N* = 1	Ia	No AE or SAE ↓40% hospitalization stay and readmission CKD was stable up to 16 mo follow-up No hemodialysis required	(14–17)
NCT01602328	Completed	Phase 2	2012	BM-MSC^a^	Allogeneic	2 × 10^6^ cells/kg body mass *N* = 1	Iv	~Recovery, need for dialysis, 30-day mortality, AE and SAE between treated and control groups	(18)
NCT01275612	Withdrawn	Phase 1	2010	BM-MSC	Autologous	1 × 10^∧^6 cells/kg *N* = 1	Iv	Patients evaluated not meet the primary criterion	(19)
NCT04194671	Not yet recruiting	Phase 1|Phase 2	2020	UC-MSC	Allogeneic	NA *N* = 2, 7 d apart	Iv		
NCT03015623	Active, not recruiting	Phase 1|Phase 2	2017	BM-MSC^2^	Allogeneic	SBI-101 + 2.5 × 10^8^ vs. 7.5 × 10^8^	Time of hemodialysis		
NCT04445220	Recruiting	Phase 1|Phase 2	2020	BM-MSC^b^	Allogeneic	SBI-101 + 2.5 × 10^8^ vs. 7.5 × 10^8^	Time of hemodialysis		
**Sepsis-Induced AKI**									
NCT02421484	Completed	Phase 1	2015	BM-MSC	Allogeneic	0.3 vs. 1 vs. 3 × 10^6^ cells/kg body mass *N* = 1	Iv	No AE or SAE ~Efficacy between treated and control groups	(20)
NCT03369275	Not yet recruiting	Phase 2	2018	BM-MSC	Allogeneic	3 × 10^6^ cells/kg body mass *N* = 1	Iv		
**Chronic and End-Stage Kidney Disease**									
NCT02966717	Active, not recruiting	Phase 2	2016	BM-MSC	Allogeneic	1 × 10^6^ cells/kg body mass *N* = 2, 2 weeks apart	iv		
NCT02166489	Completed	Phase 1	2014	BM-MSC	Autologous	2 × 10^6^ cells/kg body mass *N* = 1	iv	No AE or SAE ~ Renal function	(21)
NCT02195323	Completed	Phase 1	2014	BM-MSC	Autologous	2 × 10^6^ cells/kg body mass *N* = 1	iv		
NCT03321942	Unknown status	Phase 1	2017	AT-MSC	Autologous	NA	iv		
NCT03939741	Recruiting	Phase 1|Phase 2	2019	SVF	Autologous	1 × 10^6^ in 5 mL *N* = 1	iv		
**Focal Segmental Glomerulosclerosis**									
NCT02382874	Completed	Phase 1	2015	BM-MSC	Autologous	2 × 10^6^ cells/kg body mass *N* = 1	iv		
**Atherosclerotic Renovascular Disease**									
NCT04392206	Recruiting	Phase 1	2020	AT-MSC	Allogeneic	3 vs. 5 × 10^6^ cells/kg body mass *N* = 1	Time of hemodialysis		
NCT01840540	Completed	Phase 1	2013	AT-MSC	Autologous	1 × 10^5^ vs. 2.5 × 10^5^ cells/kg body mass *N* = 1	ia	↑ Cortical perfusion ↑ Renal blood flow ↓ Tissue hypoxia ↑ GFR 3 mo follow-up	(22)
NCT02266394	Completed	Phase 1	2014	AT-MSC	Autologous	NA	ia		
**Diabetic Nephropathy**									
NCT01843387	Completed	Phase 1|Phase 2	2013	BM-MSC^c^	Allogeneic	1.5 vs. 3 10^8^ cells *N* = 1	iv	Trend to stabilized or improved eGFR	(23)
NCT03288571	Not yet recruiting	Phase 1|Phase 2	2019	UC-MSC	Allogeneic	NA *N* = 3, 2 w apart in each kidney	intra-renal		
NCT04216849	Recruiting	Phase 1|Phase 2	2020	UC-MSC	Allogeneic	1.5 × 10^6^ cells/kg body mass *N* = 5, course of 32 w	iv		
NCT04562025	Recruiting	Phase 1	2020	UC-MSC	Allogeneic	1 × 10^6^ cells/kg body mass *N* = 3, weekly	iv		
NCT02585622	Recruiting	Phase 1|Phase 2	2017	BM-MSC^d^	Allogeneic	0.8 vs. 1.6 vs. 2.4 × 10^8^ cells *N* = 1	iv		
NCT04125329	Recruiting	Phase 1	2020	UC-MSC	Allogeneic	1 × 10^6^ cells/kg body mass *N* = 3, monthly	iv		
NCT03840343	Recruiting	Phase 1	2019	AT-MSC	Autologous	2.5 vs. 5 × 10^5^ cells/kg body mass/ *N* = 2, 3 mo apart	ia		
**Lupus Nephritis**									
NCT00698191	Completed	Phase 1|Phase 2	2007	BM-MSC/UC-MSC	Allogeneic	1 × 10^6^ cells/kg body mass *N* = 1	iv	↓ Proteinuria ↑ Disease improvement	(24–26)
NCT01741857	Completed	Phase 1|Phase 2	2012	UC-MSC	Allogeneic	1 × 10^6^ cells/kg body mass *N* = 2, 7 d apart	iv	↓ Proteinuria ↑ Disease improvement	(27)
NCT00659217	Unknown status	Phase 1|Phase 2	2008	BM-MSC	Autologous	NA *N* = 1	iv		
NCT01539902	Withdrawn	Phase 2	2012	UC-MSC	Allogeneic	5 × 10^7^ *N* = 2, 7 d apart	iv	~ Remission rates in treated and placebo groups	(28)
NCT03580291	Not yet recruiting	Phase 2	2018	UC-MSC	Allogeneic	2 × 10^6^ cells/kg body mass *N* = 2, 7 d apart	iv		
NCT03458156	Active, not recruiting	Phase 1	2017	UC-MSC	Allogeneic	1 × 10^6^ cells/kg body mass *N* = 1	iv		
NCT03174587	Completed	Phase 1	2017	BM-MSC	Allogeneic	1, 2 and 3 × 10^6^ cells/kg body mass^⊲^ *N* = 3	iv	No AE Infusion was tolerated	(29)
NCT04522505	Active, not recruiting	Phase 1	2017	BM-MSC	Allogeneic	1, 2, 3 and 10^6^ cells/kg body mass^⊲^ *N* = 3	iv		
NCT04835883	Recruiting	Phase 2	2019	BM-MSC	Allogeneic	2 × 10^6^ cells/kg body mass *N* = 2, 12 d apart	iv		
NCT04318600	Completed	Phase 1	2014	Amniotic-MSC	Allogeneic	1 × 10^6^ cells/kg body mass *N* = 3, monthly	iv		
NCT03917797	Recruiting	Phase 2	2019	UC-MSC	Allogeneic	NA	iv		
NCT03673748	Not yet recruiting	Phase 2	2021	BM-MSC	Allogeneic	1.5 × 10^6^ cells/kg body mass *N* = 1	iv		
NCT02633163	Recruiting	Phase 2	2018	UC-MSC	Allogeneic	1 vs. 5 × 10^6^ cells/kg body mass *N* = 1	iv		
**Kidney Transplant**									
NCT00659620	Unknown status	Phase 1|Phase 2	2008	BM-MSC	Autologous	NA	iv		
NCT00658073	Completed	Phase 1	2008	BM-MSC	Autologous	1-2 × 10^6^ cells/kg body mass *N* = 2, 24 h and 2 w after Tx	iv	↓ Acute Rejection ↓ Risk of opportunistic infections, ↑ eGFR 1-year follow-up	(30)
NCT00734396	Completed	Phase 1|Phase 2	2009	BM-MSC	Autologous	1 × 10^6^ cells/kg body mass *N* = 1	iv	No AE Resolution of tubulitis without IF/TA in two patients	(31)
NCT00752479	Completed	Phase 1|Phase 2	2008	BM-MSC	Allogeneic	2 × 10^6^ cells/kg body mass *N* = 1, 7 d post Tx	iv	↑ Serum Creatinine > Acute Graft Dysfunction ↑ Regulatory T cells ↓ Memory CD8^+^ T cells	(32)
						2 × 10^6^ cells/kg body mass *N* = 1, Tx	iv	↓ Memory CD8+ T cells ↓ Donor-specific CD8+ T cell cytolytic response ↑ Expansion of CD4^+^CD25^+^FoxP^+^ Treg cells	(33)
NCT02012153	Recruiting	Phase 1	2013	BM-MSC	Autologous	2 × 10^6^ cells/kg body mass *N* = 1, 1 d before Tx	iv	↑ Graft function for 5 to 7 years follow-up ↓ CD8^+^ T cell in 3 of 4 patients ↓*ex vivo* T cell donor-specific cytotoxicity	(34)
								↑ CD4^+^CD25^+^FoxP^+^ Treg cells ↑ Naïve and transitional B cells. 1 patient successfully discontinued immunotherapy with CsA	
NCT02492490	Unknown status	Phase 1|Phase 2	2014	SVF	Autologous	1 × 10^6^ cells/kg body mass *N* = 4, 0, 7, 14, 21 d after Tx	iv		
NCT02561767	Completed	Phase 1|Phase 2	2015	BM-MSC	Autologous	1 × 10^6^ cells/kg body mass *N* = 4, 0, 7, 14, 21 d after Tx	iv	No AE or SAE ↑ GFR. Renal function stable ↑ B cell levels	(35)
NCT02563366	Unknown status	Phase 1|Phase 2	2015	BM-MSC	Allogeneic	1 × 10^6^ cells/kg body mass *N* = 4, 0, 7, 14, 21 d after Tx	iv		
NCT02490020	Completed	Phase 1	2016	UC-MSC	Allogeneic	iv: 2 × 10^6^ cells/kg body mass, 48 h before Tx +/− ia: 5 × 10^6^ cells/kg body mass, during Tx *N* = 2	iv + ia	No AE No MSC engraftment → Post-operative complications → eGFR	(36, 37)
NCT02563340	Unknown status	Phase 1|Phase 2	2015	BM-MSC	Allogeneic	1 × 10^6^ cells/kg body *N* = 4, 2 w apart	iv		
NCT02492308	Unknown status	Phase 1|Phase 2	2014	SVF	Autologous	1 × 10^6^ cells/kg body *N* = 4, 0, 7, 14, 21 d after Tx	iv		
NCT02409940	Completed	Phase 1	2013	BM-MSC	Allogeneic/ Autologous	0.2-3 × 10^6^ cells/kg body *N* = 2, 1 d pre- and 30 d post-Tx	iv	No AE or SAE ↑ Graft function ↑ CD4^+^CD25^+^FoxP^+^ Treg cells ↓ CD4^+^T cell proliferation	(38, 39)
NCT02565459	Recruiting	Phase 1	2015	BM-MSC	Allogeneic	1 vs. 2 × 10^6^ cells/kg body mass *N* = 1, Tx	iv		
NCT02387151	Completed	Phase 1	2015	BM-MSC	Allogeneic	1.5 - 2 × 10^6^ cells/kg body mass *N* = 2	iv		(40)
NCT02057965	Active, not recruiting	Phase 2	2014	BM-MSC	Autologous	1 vs. 2 × 10^6^ cells/kg body mass *N* = 2, 6 and 7 w after Tx	iv		(41)
NCT03478215	Recruiting	Phase 2	2016	BM-MSC	Autologous	1, 2, and 3 × 10^6^ cells/kg body mass^⊲^ *N* = 1	iv at surgery		
NCT01429038	Completed	Phase 1|Phase 2	2012	BM-MSC	Allogeneic	1.5 vs. 3 × 10^6^ cells/kg body mass *N* = 2, 3 and 5 d post Tx	iv	No MSC engraftment 2 Kidney/MSC HLA MM 1 MSC MM	(42)

*MSC From Commercial Entities:*

a *AC607 (AlloCure Inc.),*

b *SBI-101 plasmapheresis device in combination with MSC (Sentien Biotechnologies Inc.),*

c *Rexlemestrocel-L (Mesoblast Ltd.),*

d *ORBCEL-M^TM^ (Orbsen Therapeutics Lt.).*

*~, Similar; ↑, Increase; ↓, Decrease; ⊲, Dose-escalated study.*

*AE, Adverse events; AKI, Acute Kidney Injury; AT, Adipose Tissue; BM, Bone Marrow; CsA, Cyclosporin A; d, day; eGFR, estimated Glomerular Filtration Rate; GFR, Glomerular Filtration Rate; HLA, Human Leukocyte Antigen; ia, intra-arterial; IF, Interstitial fibrosis; iv, intra-venous; Kg, Kilogram; MM, Mismatch; mo, month; MSC, Mesenchymal Stromal Cell; NA, Not Available; SAE, Severe adverse events; SVF, Stromal Vascular Fraction; TA, Tubular Atrophy; Treg, Regulatory T cells; Tx, Transplant; UC, Umbilical Cord; w, week; y, year*.

One of the current roadblocks relates to a lack of standardization of manufacturing protocols across laboratories and manufacturing centers, which hinders inter-study comparisons within the field (46) and may have significant effects on cell phenotype and performance (47–49). Heterogeneity within MSC populations is another major obstacle; there is now a growing body of literature highlighting unique and intrinsic properties according to tissue origin and donor-related features, with characteristics such as sex, age and disease status having shown to affect their properties (50–54). In this regard, although clinical data has provided evidence for the safety of MSCs (55), attention has also been given to the immune compatibility and hemocompatibility of specific MSC infusions, urging the inclusion of HLA mismatch assessment and expression of procoagulant factors within the safety release criteria (44, 46, 56).

**Figure 2:**
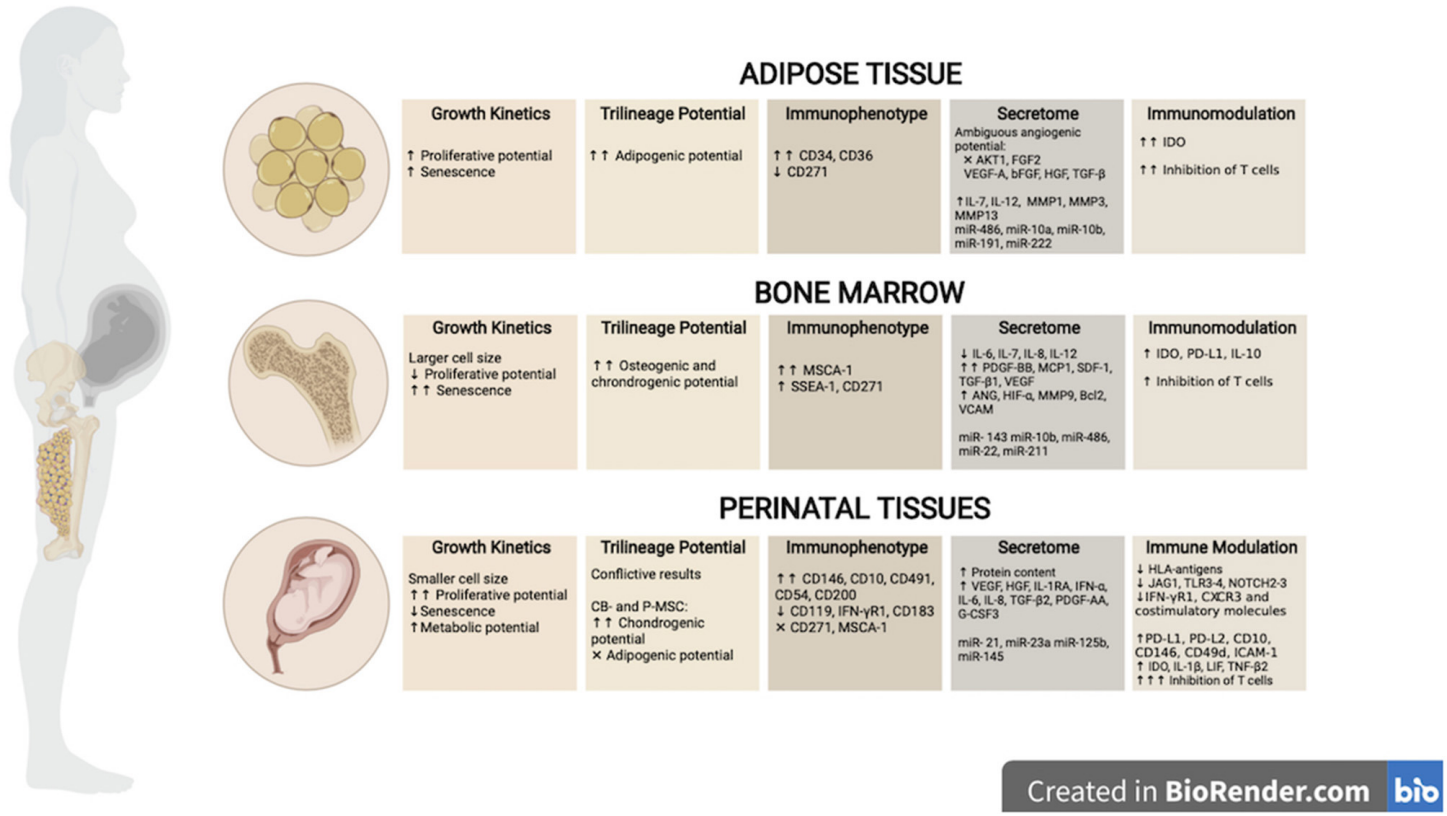
Biological properties of tissue-derived MSCs. MSCs can be isolated from adult tissue sources such as adipose (AT)- and bone marrow (BM), as well as perinatal and/or birth-associated tissues, including amniotic liquid (AM), cord blood (CB), placenta (P) or umbilical cord (UC) tissues. Tissue of origin have shown to impact the biological properties of MSCs. This figure illustrates the main differences described in the literature regarding growth kinetics, differentiation abilities, immunophenotype, secretome, and immune modulation between cell sources. Created in BioRender.com.

MSCs were initially discovered by Friedenestein et al. as a non-hematopoietic population of cells within the bone marrow (BM), that were plastic-adherent, had fibroblastic phenotype, were able to generate colonies *in vitro* and undergo osteogenic differentiation (57, 58). Later, several groups identified their ability to differentiate into other mesodermal lineages such as adipocytes and chondrocytes, and their ability to be sub-passaged and expanded *in vitro* (59, 60). Since then, MSCs have undergone an extensive diversification and cells with similar characteristics have been isolated from nearly every vascularized tissue (61) as a subgroup of pericytes that reside near vessels, contributing to their homeostasis and regenerative processes (62–64). As a summary, MSCs have been obtained from adult tissues such as adipose tissue (AT-MSCs) (65), dental pulp (DP-MSCs) (66) and other dental tissues (67), endometrium (EM-MSCs) (68, 69), menstrual blood (Men-MSCs) (70), peripheral blood (PB-MSCs) (71, 72) and from several perinatal and birth-associated tissues, referred hereafter as perinatal tissue-MSCs (PT-MSCs) including MSCs from amnion membrane (AM-MSCs), amniotic fluid (AF-MSCs), umbilical cord blood (CB-MSCs), placenta (PL-MSCs), umbilical cord tissue (UC-MSCs) and Wharton's jelly (WJ-MSCs) (73–78) (Figure 2). It is important to note that placental tissue can be fetal or maternal in origin, and therefore, MSCs derived from the two types of tissue should be individually characterized.

Current studies focus on trying to understand the mechanistic characteristics underlying MSC-like cells and their therapeutic effects with respect to the tissue of origin. To date, little is known about tissue-specific properties being able to predict clinical efficacy. Considering the significant effect that origin may have on functional properties, and possible therapeutic outcomes, it has now been recognized that the choice of cell source should be considered when optimizing manufacturing protocols for particular clinical applications. In addition to this attention to the source of MSCs, efforts should focus on developing more homogeneous manufacturing approaches to reduce inter-study variability and improve the interpretation and comparability of results from different centers, which ultimately will help to advance the field. Nevertheless, it seems plausible that an ultimate consensus or harmonization will not be reached due to reasons such as intellectual property as well as infrastructure and resources available for large-scale production.

In this article we provide a comprehensive review on the origin-specific biological characteristics of MSCs and their use in current clinical trials in a range of renal pathologies, and attempt to identify intrinsic biological characteristics with beneficial effects. We have also reviewed the literature regarding culture processing variables that are important to consider during the scale-up and manufacture of the cell product. As part of this study, we conducted a search in the ClinicalTrials.gov database of current registered clinical trials on kidney disease. According to our search, fifty-four clinical trials are being or have been conducted around the world to study the safety and efficacy of MSC-based ATMPs in a variety of renal diseases (accessed in April 2021, https://clinicaltrials.gov) (Table 1). We acknowledge that this list may not be exhaustive as it is derived from one well-known clinical trial registry, and it is possible that some other clinical trials may be listed in other national or international registries, which have not been considered in this review. The search includes clinical studies at all different stages (completed, recruiting, or not enrolling). Search terms included: “mesenchymal stem cells,” “mesenchymal stromal cells,” “kidney injury,” “kidney disease,” “kidney transplant,” combined in various modifications with “AND” and “OR.” When possible, information about MSC tissue source, donor (allogeneic or autologous), and cell processing variables such as cell plating densities, passage number, culture media supplements and culture devices for cell expansion, were extracted (Table 2). When available, additional sources of information were retrieved from hand searches of relevant papers and/or websites.

**Table 2:** Summary of MSC culture processing variables publicly available from clinical trials of KD.

**NCT number**	**Cell source**	**Donor origin**	**Media supplements**	**Cell densities**		**Passage number**	**Cell culture system**	**References**
				**Isolation**	**Plating**			
**Acute Kidney Injury**								
NCT00733876	BM-MSC^a^	Allogeneic					2D Flask	(79)
NCT01602328	BM-MSC^b^	Allogeneic					2D Flask	(18)
NCT03369275	BM-MSC	Allogeneic	XF				3D Bioreactor	
NCT02421484	BM-MSC	Allogeneic	XF				2D Flask	(20)
NCT01840540	AT-MSC	Autologous	5% hPL	100, 000 cells/cm^2^	100 – 250 cells/cm^2^		2D Flask	(22, 80)
**Chronic Kidney Disease**								
NCT02166489	BM-MSC	Autologous	10% FCS			P1-3	2D Flask	(21)
**Diabetic Nephropathy**								
NCT01843387	BM-MSC^b^	Allogeneic	10% FCS			P2	2D Flask	(23)
NCT02585622	BM-MSC^c^	Allogeneic					3D Bioreactor	
**Lupus Nephritis**								
NCT00698191	BM-, UC-MSC	Allogeneic	10% FCS	100, 000 cells/cm^2^	1, 000 cells/cm^2^	P2-P5	2D Flask	(24–26)
NCT01741857	UC-MSC	Allogeneic	10% FCS	100, 000 cells/cm^2^	1, 000 cells/cm^2^	P3-P5	2D Flask	(27)
**Kidney Transplant**								
NCT01429038	BM-MSC	Allogeneic	10% FCS			P2	2D Flask	(81)
NCT00658073	BM-MSC	Autologous	HSA			P3-4	2D Flask	(30)
NCT00734396	BM-MSC	Autologous	10% FCS	160, 000 cells/cm^2^	4, 000 cells/cm^2^		2D Flask	(31)
NCT00752479	BM-MSC	Allogeneic	5% hPL	200, 000 cells/cm^2^	200 cells/cm^2^	12 d in culture	2D Flask	(33)
NCT02492490	SVF	Autologous	10% FCS				2D Flask	
NCT02561767	BM-MSC	Allogeneic	10% FCS				2D Flask	(35)
NCT02490020	UC-MSC	Allogeneic	HSA				2D Flask	(36, 37)
NCT02409940	BM-MSC	Allogeneic/Autologous	10% hPL	200, 000 cells/cm^2^	500, 000 cells/cm^2^	P3	2D Flask	(38, 39)
NCT02012153	BM-MSC	Autologous	5% hPL	200, 000 cells/cm^2^	100–200 cells/cm^2^	P1	2D Flask	(34)

*AT, Adipose Tissue; BM, Bone Marrow; FCS, Fetal Calf Serum; HSA, Human Serum Albumin; hPL, Human Platelet Lysate; SVF, Stromal Vascular Fraction; UC, Umbilical Cord; XF, Xenofree media.*

*MSC Commercial Names:*

a *AC607 (AlloCure Inc.),*

b *Rexlemestrocel-L (Mesoblast Ltd.),*

c *ORBCEL-M^TM^ (Orbsen Therapeutics Lt.)*.

## MSC-Based Therapies in Kidney Diseases

### Disease Overview

Generally, kidney diseases have been subdivided into acute kidney injury (AKI) and chronic kidney disease (CKD), according to the duration of the injury. While AKI is described as an abrupt decline in renal function, CKD emerges after years of progressive and persistent loss of glomerular filtration rate and albuminuria (4, 5). Although they were originally considered two individual entities, it is now clear they share an intrinsic link: maladaptive repair following AKI leads to progressive CKD, and at the same time patients with underlying CKD are more likely to develop AKI resulting in a deterioration in renal function (82). Often asymptomatic, the progressive nature of CKD leads to a vicious cycle of injury that ultimately causes renal failure or end-stage renal disease (ESRD) (83). At the time of late-stage CKD diagnosis, renal function has declined beyond physiological reserve and kidney failure is diagnosed. Despite significant advances in understanding the pathophysiology of AKI and CKD, current therapeutic and pharmacological approaches only offer supportive treatment to handle and manage underlying complications (84). In recent years MSCs and their derived by-products, mainly paracrine signals and extracellular vesicles (EVs), have emerged as a novel cell-based therapy to treat acute and chronic kidney injury. Herewith, this section reviews the growing body of preclinical and clinical evidence on the potential role of MSCs in recovery after kidney damage.

### Mechanisms of Action of MSCs in Kidney Disease

#### MSCs in Acute Kidney Injury

AKI is considered a severe clinical syndrome in hospitalized patients, with a prevalence of 1–9%, and is especially common among critically ill patients, affecting 45% of patients admitted to the intensive care unit (85). The abrupt decline in renal function is accompanied by an alteration of the homeostasis within the kidney. The decline in glomerular filtration rate results in accumulation of serum creatinine, blood urea nitrogen (BUN), and/or reduction in urine output (85). Along with the original insult, functional disturbances cause a reduction in renal cell mass due to cell death, impairing renal function and facilitating the subsequent progression to fibrosis. Notwithstanding all efforts to manage the associated clinical manifestations, AKI is still considered an independent risk factor for mortality and development of CKD (86). Within the multiple etiologies of renal injury, ischemia-reperfusion injury (IRI) is the most prevalent form of AKI, together with vascular obstructions within the renal circuit (85), and drug-induced nephrotoxicity (87). IRI is also an unavoidable event during kidney transplantation, limiting graft functionality and increasing the risk of rejection and graft loss (88).

Inflammation plays a central role throughout the process of kidney injury (89). Shortly after the injury, activation of inflammatory pathways induces the recruitment and infiltration of leukocytes such as neutrophils, monocytes, and dendritic cells. T and B lymphocytes have also been linked to contributing to kidney injury. Conversely, regulatory T cells and M2 macrophages are essential in suppressing inflammation, enhancing tissue remodeling and causing repair. However, if uncontrolled, the endogenous mechanisms of tissue repair within the kidney could promote additional damage and irreversible fibrosis (90). Together, the immune system, the ischemic environment and the endogenous mechanisms of repair converge in a complex milieu of profibrotic and proinflammatory cytokines and chemokines. In this context, MSCs have been proposed as powerful candidates to dampen the severity of AKI and promote effective regenerative processes.

Infusion of MSCs in several *in vivo* models of AKI has resulted in improved renal function by decreasing tubular injury, promoting angiogenesis, reducing oxidative stress as well as inflammation, promoting a pro-regulatory and anti-inflammatory phenotype (91–93). The main mechanisms whereby MSCs have been found to elicit such renoprotective effects are related to paracrine signaling and shedding of extracellular vesicles (79, 94). MSC-based therapies have been proven to stimulate the regeneration of tubular epithelial cells by increasing intra-renal levels of HGF (95–97) and TSG6 (98), promoting the activation of pro-survival pathways such as AKT/ERK (99); decreasing tubular apoptosis, by upregulating Bcl2 and downregulating Caspase 3 (100), and inhibiting the endoplasmic reticulum stress response (99). Moreover, MSCs help in counterbalancing the oxidative damage by enhancing the activity of free radical scavengers (101), favoring the activation of the Nrf2/ARE pathway (102) and downregulating the expression of NOX2 which are key ischemia-related insults (102). A large part of the beneficial effect of MSCs is related to their interaction with both, the innate and adaptive immune systems. The complement system serves as a key moderator of the immune system and MSCs have been described to interact with this system in a synergistic manner to modulate the host immune response (103). Conversely, in the context of kidney injury, MSCs have been found to inhibit the overactivation of the complement cascade, decreasing serum levels of C5a as well as intra-renal deposits of C3 and C5aR (104, 105). Downregulation of inflammatory cytokines such as TNFα, MMP9, ICAM1, NFκB (100, 106, 107) and chemokines such as CX3CL1 (108), CXCL2, and IL6, decreased the infiltration of pro-inflammatory macrophages (109) and effector T cells while promoting the presence of regulatory T cells (110). This “shift” toward an anti-inflammatory profile seems to be, in part, governed by the expression of IL10 (111) and adherence factors such as ICAM1 and VCAM1 (112). The secretion of pro-angiogenic factors [e.g., VEGF, eNOS (113–116)] has been shown to improve capillary rarefaction (107), dampening the ischemic damage and preventing the progression of interstitial fibrosis (108, 110).

Interestingly, *in vitro* experiments have found that small single-stranded non-coding RNA molecules (miRs) contained within EVs produced by BM-MSCs can protect proximal tubular epithelial cells after ischemia by targeting the expression of mRNAs associated with apoptosis, cytoskeleton reorganization, fibrosis, and hypoxia (117), endowing EVs and their miR cargos with interactive roles in the regenerative process.

Recently, a novel mechanism of action has been proposed whereby MSCs could rescue damaged tubular cells by targeting mitochondrial dysfunction and sustaining their energy supply (118), and restoring physiological dynamics (119). Another consideration in the therapeutic use of MSCs is the use of genetic modification (120–122) as well as pre-conditioning strategies such as hypoxic culture conditions (123–125), and priming of cells (126–128), which have showed superior therapeutic potential compared to that of unmodified controls (129).

#### MSCs in Chronic Kidney Injury and End-Stage Renal Disease

CKD emerges as the result of continuous kidney damage and scarring mediated by a dysfunctional inflammatory status (130, 131). The perpetuation of the injury is often a result of high blood pressure, nephrolithiasis, and several underlying conditions such as diabetes mellitus (10), systemic lupus erythematosis (132), or glomerular pathologies (133), as well as the development of *de novo* AKI (134). Regardless of the initial insult, the exacerbated renal fibrosis response that occurs throughout the course of the disease induces morphological alterations with physiological and functional consequences (135). Progression to ESRD is, therefore, inevitable.

Paracrine signaling and/or EVs derived from MSCs have been transiently found within the glomeruli and injured tubules, limiting the extent of the injury by alleviating interstitial fibrosis, recruiting leukocytes, and activating intrinsic repair mechanisms that prevent AKI-CKD transition (136–139). Similar effects have been described in several models of established CKD, where cell and cell-free strategies resulted in reduced accumulation of fibrotic tissue as a result of decreased expression of extracellular matrix components and increased capillary density, attenuation of the pro-fibrotic and pro-inflammatory environment, and promotion of M2 anti-inflammatory macrophages (140–142). However, attenuation of inflammation is not always achieved, probably due to differences in treatment time and frequency (143). In these circumstances, “licensing” strategies have proven to be efficient in promoting an early onset of MSC therapeutic effects (128).

Several studies have also explored MSC therapies in chronic scenarios where renal damage is being perpetuated by underlying pathologies, predominantly autoimmune nephritis caused by systemic lupus erythematosis (SLE) and microvascular complications of diabetes mellitus, commonly referred to as diabetic nephropathy (DN). In both scenarios, preclinical models have described the usefulness of MSCs in ameliorating the pathogenic manifestations albeit through different mechanisms due to the different nature of the insults. MSCs in preclinical models of lupus nephritis (LN) have been shown to act by suppressing the activation of the humoral and cellular immune response, evoking a systemic pro-tolerogenic milieu (144–146). Besides regulating leukocyte infiltration and inhibiting proinflammatory cytokines, beneficial actions in DN models have been also attributed to the reduction of systemic biochemical alterations and reducing renal levels of oxidative stress, apoptosis, and fibrosis while promoting renal regeneration (147–150).

#### MSCs in Kidney Transplantation

One of the most relevant clinical settings where MSCs have shown potential beneficial effects is renal transplantation. In murine models of kidney transplantation (KTx), infusion of autologous (151, 152) or syngeneic (153, 154) MSCs induced graft tolerance and recipient survival. The achievement of a pro-tolerogenic environment was, in part, mediated by the production of indoleamine 2, 3 dioxygenase (IDO), crucial in generating regulatory FoxP3^+^ T cells (112, 151). The effect was increased when BM-MSCs were licensed with the pro-inflammatory cytokine IL17A (152, 155). However, administration of MSCs was found to only elicit a tolerogenic response and enhanced graft survival when administered following graft transplantation (112, 153, 154).

Comparable effects have been reported in rodent models where single (156, 157), and multiple (158) administrations of MSCs resulted in significant improvement in graft function and attenuated expression levels of pro-inflammatory cytokines (156–158). Licensing with TGFβ1 (159) or genetic modifications to overexpress CXCR4 (160) enhanced the immunosuppressive abilities and showed an increased induction of regulatory T cells and anti-inflammatory cytokines. Beneficial effects have also been reported in attenuating cellular infiltration and tubular damage due to chronic graft rejection (156, 161, 162). In contrast with this favorable preclinical evidence, other studies have reported that administration of MSCs and their derived EVs did not exert similar beneficial effects (163, 164), highlighting the impact of timing and synergistic immunosuppressive strategies to ensure robust therapeutic effects.

### Clinical Translation of MSC Therapies in Kidney Disease

**Figure 3:**
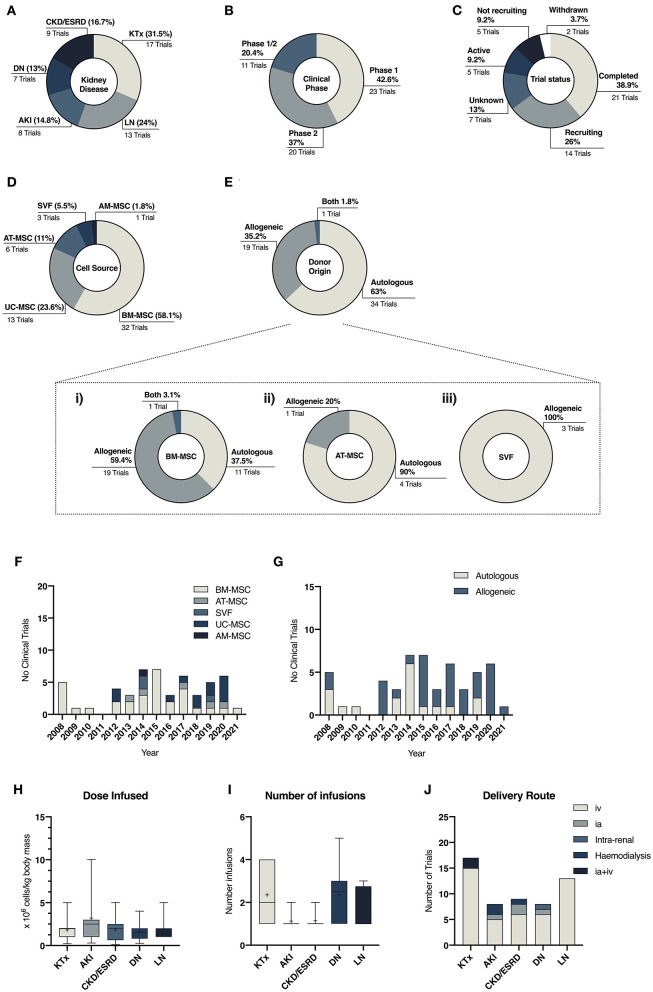
Descriptive data related to clinical trials in kidney diseases comparing the number of trials per disease **(A)**, clinical phase of the studies **(B)** and their status **(C)**. **(D,E)** Illustrate the heterogeneity of cell and donor source across all studies, while **(F,G)** depicts the change of cell and donor source preferences over the years. **(H–J)** illustrate protocol differences across different disease settings related to dose of MSC infused **(H)**, frequency of infusions **(I)** and choice of delivery route **(J)**.

Promising preclinical results, described above, have led to early-phase clinical studies that investigate the safety and efficacy of MSC-based therapies in a wide range of renal pathologies. Based on data compiled from studies registered on clinicaltrials.gov (accessed in April 2021), a total of 54 been registered since 2008. The main results of our search are summarized in Figure 3 and expanded in Table 1, which present an overview of the clinical use of MSCs in kidney disease highlighting heterogeneity in terms of tissue source and product development characteristics. Results from this search showed that MSCs have been most commonly used to improve outcomes of kidney transplant procedures (31.5%), targeting either induction of allograft tolerance or minimizing the use of immunosuppressive drugs. Other trials have focused on the renoprotective potential of MSCs in lupus nephritis (24%), AKI (14.8%), diabetic nephropathy (13%), and CKD/ESRD (16.7%) (Figure 3A). Nevertheless, MSC therapies for these conditions have yet to reach later stage clinical trials and market authorization (Figure 3B).

Results from our literature search have also highlighted the great heterogeneity within the field in terms of donor and tissue source, mode of cell delivery and cell dose (Table 1). During the last decade, BM-MSCs have been the predominant cell source in clinical trials (7). However, clinical MSC products have greatly diversified in the past decade, with equal use of BM-, AT-, and PT-MSC products in clinical trials (44, 165). In the kidney disease clinical arena, the use of BM-MSCs remains the predominant source (58%), although in the past 10 years a diversification on the use of different MSC sources have also been noted (Figures 3D–G).

Most trials have used intravenous delivery (Figure 3J), despite studies which have shown that the majority of MSCs are trapped within the pulmonary circuit (166, 167). Some studies have explored the combination of intra-arterial delivery, to facilitate homing of the cells within the kidney, and intravenous infusion (35, 36). Nevertheless, in both cases, hemocompatibility and levels of procoagulant tissue factor (TF/CD142) should be considered to avoid the onset of the instant blood-mediated inflammatory reaction (IBMIR), as it is an important aspect for the safety and efficacy of these therapies (168). Although clinical protocols have added anti-thrombotic drugs to ensure the safety of MSC products (169), several studies have reported that TF/CD142 expression varies between MSC tissue origin and is highly impacted by culture processing as well as cell-dosage (44, 168, 170). Given the procoagulant nature of MSCs, safety characterization is of utmost importance and even more so in patients undergoing organ replacement therapies that carry the burden of strong immunosuppressive regimes and their side effects such as increased cardiovascular risk (56, 171).

With the increasing presence of allogeneic therapies in the past years (Figure 3G), greater considerations should also be placed on the potential impact of MSC immunogenicity and the generation of alloreactive immune responses, as little is known of its long-term clinical implications (172, 173). Despite the presence of extensive evidence showing anti-donor cellular and humoral immune responses following administration of allogeneic MSCs (174, 175), presence of donor-specific human leukocyte antigen (HLA) antibodies has been minimally considered previously (172). This will represent a major risk when repeated injections are being included in therapeutic procedures for potentially pre-sensitized patients such as those undergoing kidney transplantation (56, 173).

Finally, another aspect to consider is whether MSCs are delivered as culture-adapted or “fresh” cells, with optimal metabolic fitness, or cryobanked “off-the-shelf” cells, which are thawed immediately prior to transplantation. While this is an important aspect for efficacy and safety of MSC therapies, results from our search, and others (176), have reported that the method of cell delivery (fresh vs. cryobanked), is often omitted or not clearly stated in manuscripts. The tendency to use cryobanked “off-the-shelf” cells has increased over time (48, 176), most likely due to the logistic advantages of this approach. However, controversy revolves around the use of banked products, with studies demonstrating reduced therapeutic potential, loss of functionality and increased susceptibility to trigger prothrombotic events (177–181), while others have showed minimal impairment of cell viability and fitness (182, 183). In this context, clinical potency has been linked with the concept of metabolic fitness and product viability at the time of infusion (184, 185) making it critical to develop manufacturing methods to rescue cryopreserved cells and restore cell functionality (186). Intriguingly, other studies have demonstrated that apoptotic or dead MSCs, and therefore less metabolically active, confer therapeutic benefits by enhancing the host innate immune response (187–189). Gaining better mechanistic insights behind the benefits that MSC therapies elicit, may it be due to viable cells, their derived by-products or rather by immune activation through dying/dead cells holds the key to elicit better therapeutic outcomes (186, 190).

#### Kidney Transplantation

Based on their immune-privileged characteristics, MSCs have been administered in conjunction with RRT to promote graft tolerance and control the host immune system with hopes of enhancing the withdrawal or minimization of immune-suppressive therapies and enhancing organ function. Initial results from a pilot study published by Perico et al. revealed the importance of timing of cell delivery. Autologous infusion of BM-MSCs in two patients seven days after undergoing KTx from a living related donor caused a severe decline in renal function and humoral and cellular acute rejection (32). The post-surgery subclinical inflammatory environment upon which MSCs were transplanted seemed to favor the development of a pro-inflammatory phenotype that could have contributed to an early graft dysfunction (154). Pretransplant administration did not result in impaired graft function, highlighting the paramount relevance of protocol optimization (33). Moreover, it showed a pro-tolerogenic graft environment supported by reduced effector CD8^+^ T cells and expanded regulatory CD4^+^FoxP3^+^ T cells that led to stable graft function after long-term follow-up (34). In one patient, long-lasting counterbalance of regulatory/effector T cells and increased presence of B cells allowed the successful discontinuation of the use of ciclosporin A and tapering of the dose of immunosuppressive drugs (34).

Several other studies have provided further insights into the applicability of MSC in kidney transplant from living related (31, 35, 36, 38) and deceased donors (36, 191). Using kidneys from brain or cardiac deceased donors would potentially increase the number of transplant recipients and meet the growing need for kidney grafts (192). However, these procedures are associated with a higher incidence of early graft dysfunction and acute rejection as prolonged ischemic time exerts an adverse event on graft survival (193, 194). Recently, the combinatorial infusion of UC-MSCs before and during surgery in recipients of deceased donor grafts was proven to be safe and resulted in no adverse clinical events. However, no significant benefit was seen in terms of reduction of postoperative complications, survival rates and graft function (36, 37). A larger study would possibly facilitate a full assessment of improvement in delayed graft function, as a lower incidence was seen in the MSC treated group (36). In a much smaller trial, third party MSCs were infused in five kidney transplant recipients from deceased donors 3–5 days after the procedure. The 6-month safety interim report revealed no graft rejection but some degree of immunization against the shared kidney and MSC donors (191).

Despite the absence of treatment-related serious adverse events in the studies described so far, a side effect of MSC systemic immunosuppressive activity was reported in a small Phase I study, where three out of six patients developed opportunistic viral infections after MSC-infusion (31). Yet, in a much larger study involving 156 patients, inoculation of BM-MSCs resulted in a significantly decreased risk of opportunistic infections (30). Although no neoplastia-related events have been described in KTx, this stresses the importance of carefully monitoring MSC preparations and monitoring infused patients, particularly in elderly and chronically immuno-suppressed patients with an increased risk to develop tumors and infections.

**Figure 4:**
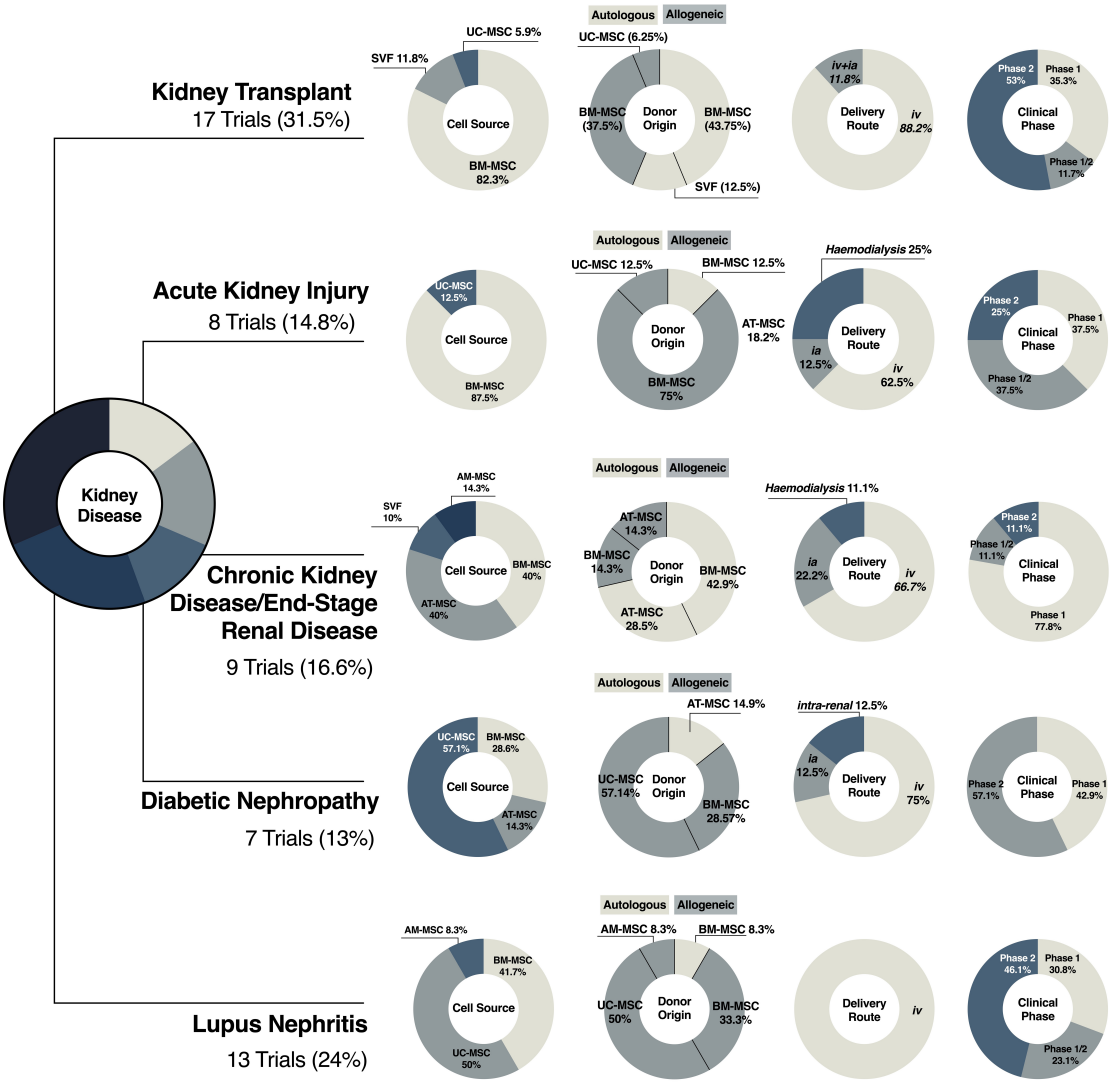
Clinical translation of MSC therapies in kidney disease. Illustrative representation of the diversification of MSC-based products in clinical trials of kidney transplantation, acute kidney injury, chronic kidney disease, diabetic nephropathy and lupus nephritis, including cell source and donor origin, and clinical variables, such as delivery route and clinical phase in.

To date, a total of eight clinical trials have been completed and results published, while nine more are yet to be completed or with no publicly available results (Figure 4). The main differences between studies can be seen between the cell source and dose regimen, as well as infusion timing and frequency (Figure 4). So far, BM-MSCs have been the choice of starting material in fourteen studies (82.3%), six using autologous (43.75%) and eight using allogeneic cells (37.5%); only two studies used SVF from autologous fat tissue and one from UC-MSCs (Figure 4). On review of the published literature, no conclusions can be drawn to determine differences in clinical outcomes on the tissue source.

Finally, although we have previously discussed the effects of timing, the rationale for administration weeks after surgery seems to be directed toward generating a pro-tolerogenic environment that would help in easing the withdrawal or tapering of immunosuppressive drugs (34). Results from current ongoing studies looking at whether MSCs in combination with mTOR inhibitor everolimus can be used for tacrolimus withdrawal may be able to shed light on the use of MSCs as a long-term effective immune-suppressive strategy (40, 41).

#### Acute Kidney Injury

Limited attempts with contradictory results have resulted from the exploration of the safety and efficacy of MSCs in recovering renal function after post-cardiac surgery AKI. An exploratory phase I trial studied the safety and feasibility of infusing allogeneic BM-MSCs in patients with several underlying comorbidities at high risk of developing AKI after open-heart surgery (14, 15). Outcomes from the first five patients showed that prevention infusion of MSCs was safe, averted postoperative decline in renal function, and decreased time of hospitalization and rates of readmission. Moreover, patients with underlying CKD had stable renal function and no disease progression after 16 months follow-up (16).

These encouraging results contrast with those from a recently published randomized, double-blind, phase II study with subjects undergoing cardiac surgery with evidence of early AKI development. Administration of commercial allogeneic MSCs (AlloCure Inc.) after AKI development did not improve the time of renal function recovery, rates of adverse events, need for dialysis or 30-day mortality (18). However, the authors recognized that infusion in an overwhelming status of the disease could have hampered the potential benefits. Further studies should aim to determine whether more favorable effects could be seen in prevention studies, such as the trial by Tögel and Westenfelder described above, rather than interventional studies when MSCs are administered after AKI onset (195).

It is well-known that sepsis, among other pathologies, can lead to the development of AKI in critically ill patients (196). A phase I study explored the safety and tolerability of administered allogeneic BM-MSCs in nine patients with septic shock (20). No infusion-associated or serious adverse events were detected, and no AKI outcomes reported. A follow-up phase II study (NCT 03369275) will further examine the efficacy of MSCs in this context.

As the regenerative medicine field evolves, new strategies are being developed to combine the use of cell-based therapies with cutting-edge biomedical devices (197–199). In this context, a phase I study is looking at the safety and tolerability of a biologic/device combination product called SBI-101 (Sentien Biotechnologies, Inc. USA). It combines a plasmapheresis device with allogeneic BM-MSCs and is designed to regulate inflammation and promote tissue repair. Two experimental cohorts using a low and high dose of MSC will be tested in AKI patients receiving continuous renal replacement therapy (NCT03015623). Furthermore, a second phase I/II trial (NCT04445220) aims to explore the use of this same device in COVID-19 patients that develop AKI.

The limited and contradictory clinical data available on the use of MSCs in AKI as well as the lack of mechanistic results challenge the possibility of drawing therapeutic roadmaps to guide the use of stromal cells in this context. Most studies related to AKI have used allogeneic BM-MSCs, emphasizing the relevance of “off-the-shelf” therapies in acute settings, where immediate therapy is needed (Figure 4). The exception is a phase I study that aimed to explore the use of autologous BM-MSCs for cisplatin-induced AKI in patients with solid organ cancer (NCT01275612). Unfortunately, none of the screened patients met the primary criterion of acute renal failure, and the study was withdrawn.

In terms of cell product preparations, illustrated in Figure 4, limited data is available; intravenous administration or infusion through the left carotid or femoral artery are the preferred delivery option whereas cell doses are inconsistent within studies (Figure 4). Timing and frequency of infusion, patient selection, guided by more sensitive biomarkers (200), and cell preparation, are some of the concerns that will have to be addressed in future preclinical and clinical studies to establish reliable therapeutic strategies.

#### Chronic Kidney Injury and End-Stage Renal Disease

Autosomal dominant polycystic kidney disease (ADPKD) is a genetic disease characterized by progressive formation and enlargement of cysts in multiple organs that have a critical effect on kidneys. The infusion of autologous BM-MSCs in a small cohort of ADPKD patients was safe and well-tolerated albeit did not improve renal function (21).

Atherosclerotic renovascular disease is the most common cause of secondary hypertension and leads to deterioration of renal function due to insufficient vascularization and ischemia (201). Current treatments based on blood flow restoration have proven unsuccessful to recover kidney injury upon damage (202). Results from a phase I study showed increased cortical perfusion and decreased renal hypoxia after infusion of autologous AT-MSC, suggesting a beneficial effect of MSCs through amelioration of the inflammatory environment and enhancement of angiogenic properties (22).

Interestingly, not only MSCs but also their by-products are being tested for CKD. In 2016, Nassar et al. administered MSC-derived extracellular vesicles from CB-MSCs in patients with CKD. Intravenous infusion resulted in significant improvement of renal function and increase in blood levels of immunoregulatory cytokines (203). Although preliminary, this study opens the window for novel strategies based on EVs derived from cultured MSCs with the potential of developing cell-free therapies (204).

In the context of CKD and ESRD, there is a tendency toward the use of autologous therapies, either from BM or AT. Although it has been recognized that MSC potency could be affected by disease status, several studies have supported the use of autologous strategies in CKD patients (205–207). Moreover, the use of allogeneic material is less desirable in patients likely to undergo renal replacement, as immune responses against donor antigens following MSC infusion have been documented (175). Overall, clinical protocols agree on administration route, frequency, and cell dosage (Figures 3H–J, 4). It should be considered that underlying patient characteristics, such as disease stage, and the intrinsic complexity of chronic diseases could be reducing any therapeutic benefit.

#### Diabetic Nephropathy

Diabetic nephropathy (DN) is the most common cause of end-stage renal failure (208). In a multicentre, randomized, placebo-controlled, dose-escalation study conducted in Australia, the safety of two doses of allogeneic BM-MSCs product rexlemestrocel-L (Mesoblast Inc.) was tested in a cohort of patients with advanced DN. No adverse events related to the treatment were reported and patients showed trends of renal function improvement after 12 weeks (23). A similar phase I study conducted in 18 different centers across the USA in patients with type 2 diabetes, showed a decrease in glycated hemoglobin 12 weeks after infusion of the highest dose (209). Although the same MSC preparation and doses were used, improvement in glycaemic control was not observed in the Australian cohort. Both studies demonstrate that infusion of MSCs in diabetic patients is safe and well-tolerated; however, their results enhance the idea that therapeutic outcomes elicited by MSC transplantation may be largely influenced by the disease stage. Results from ongoing trials may be able to confirm the suggestive effects of MSCs in restoring renal function and potentially ameliorating biochemical alterations in DN patients.

Of interest, trials related to DN present a higher heterogeneity in choice of tissue source (five trials using UC-, two BM- and one AT-MSCs) but not in donor origin, with 85% of the trials using allogeneic sources, most likely because the impact of DM on MSCs is still under evaluation (210, 211) (Figure 4). Compared with other trials, protocols have included higher cell doses, with dose-escalation studies looking at fixed doses, and a range of administration frequencies, ranging from a single intravenous injection up to five doses over 32 weeks (Figure 3I).

#### Lupus Nephritis

Systemic lupus erythematosus is an auto-immune disease characterized by the loss of immune tolerance against self-antigens that affects tissues throughout the whole body (132). Lupus nephritis (LN) is the most common clinical manifestation (212). Considering the ability of MSCs to promote a tolerogenic environment and modulate the immune system, 13 clinical trials have explored the safety and efficacy of BM- or UC-MSCs in LN patients. In this particular scenario, therapies are mainly based on allogeneic cell products (Figure 4) due to impaired immune-modulatory properties and increased senescence in patient-derived BM-MSCs (213).

Results from successful trials identified serum levels of IFNγ as a predictive biomarker of MSC therapeutic efficacy. IFNγ have also been shown to stimulate the levels of IDO (214) and to have a critical role for UC-MSCs in regulating the innate immune system through up-regulation of tolerogenic dendritic cells (145).

However, conflicting results have been published reflecting the heterogeneity among SLE and LN patients, as well as the added challenge of intrinsic confounding factors such as different degrees of disease severity and treatment regimens. While a single intravenous administration of allogeneic BM- (24) and UC-MSCs (25) was proven to evoke clinical improvement and disease remission over time (215), other studies failed to reproduce the aforementioned results (26).

To date, there are 6 more registered trials where allogeneic BM- or UC-MSCs infusion have been or are being tested in safety and efficacy exploratory studies. Based on our search, similar therapeutic regimens—cell dose, frequency, and infusion route (Figures 3I, 4)– have been or are being explored, with limited success when trying to increase infusion frequencies (28, 215) and adjust cell dose (29). Unfortunately lack of details about product preparation limit further inter-study comparisons.

## Biological Properties of MSCs Derived From Different Tissue Sources

The term “MSC” is nowadays used as an umbrella term that encompasses a variety of progenitor cells retrieved from a number of different tissues. This diversity has generated significant interest in further investigating the properties of MSCs derived from different in this review we have described the pre-clinical and clinical use of MSCs in kidney disease in which the cells have most often been harvested from bone marrow, adipose tissue and umbilical cord. It is not clear what is the rationale for use of MSCs from a particular source and if specific properties of the cells based on tissue of origin would suggest the superiority of one. In this section, we provide a comprehensive description of the biological and functional characteristics of MSCs reported in the literature depending on their tissue source to reflect further on this consideration (Figure 2).

### Cell Morphology

MSCs are widely known for exhibiting a common spindle-shaped morphology, notably at the early stages of *in vitro* culture. Although this is the axiom from different starting tissues, cell size and morphology have been shown to vary between adult and younger sources, with perinatal-derived MSCs being relatively smaller and BM-MSCs cultures bearing heterogeneous populations (216–218). Morphological differences have also been reported between UC- and AM-MSCs even when the same genetic background was shared (216). Culturing conditions can also influence cell shape, as morphological changes have been attributed to different media compositions (219) and the use of specific supplements and growth factors (220). Besides altering lifespan, aging also influences cell morphology: older, senescent cells have a larger diameter (54, 221, 222). Interestingly, differences in cell size at early passage have been linked with differential expansion potential and senescence levels (223).

### Growth Kinetics

MSC growth is characterized by an initial lag phase, where cells attach to the growing surface, followed by a log phase when cells undergo exponential growth by mitotic division. Finally, cells reach a plateau phase in which mitotic division continues but at a slower rate, as cell division is inhibited by cell-to-cell contact. This *in vitro* growth pattern continues at every passage until the hallmarks of replicative senescence start to appear, such as an increase in cell size, cell cycle arrest, interruption of mitotic divisions and accumulation of cellular debris and stress fibers (224). For clinical and experimental purposes, MSCs must undergo *ex vivo* culture expansion to generate sufficient cell numbers. However, long-term culture expansion (or *in vitro* aging) has been shown to reduce the replicative lifespan and prompt the onset of senescence (54, 225, 226). This is an important fact, as it may limit the usefulness of these cells in cases where a high degree of *ex vivo* expansion is needed such as that required for achievement of clinical therapeutic doses. Thus, in a “space race” to discover which is the best MSC source for clinical applications, the ability to withstand longer periods in culture before reaching the onset of senescence is considered to be advantageous.

Many studies have now been performed that compare the expansion potential of MSCs obtained from different tissue sources, using culture parameters such as passage number, cumulative population doubling (CPD) and doubling times (DT) to describe cellular aging. When comparing the proliferation of MSCs harvested from different tissue sources, BM-MSCs have been shown to exhibit slower proliferation rates, with DT ranging from 40 to 60h depending on the culture conditions, and earlier appearance of senescence markers in relatively early passages (between passage 6 and 7) (217, 227–231). In contrast, AT-MSCs have shown faster proliferation rates (DT of 20 to 45h) as well as the ability to sustain a longer time in culture (up to passage 8) without any signs of senescence (217, 230, 232–234). These differences were still evident when comparing proliferation and differentiation capacity of AT- and BM-MSCs harvested from the same individual, although significant degrees of donor-to-donor variability was observed (232–234). Variables such as donor, age, sex, and disease status may have a significant effect on MSC characteristics (50, 51, 53, 235), which may discourage the use of adult sources as therapeutic agents while favoring MSCs obtained from birth-associated tissues (231). In general, these cells have exhibited higher proliferative kinetics with lower CPD over time (217, 230, 236–243), often related to lower expression of senescence-associated markers or later onset of senescence (229, 244), as well as upregulation of cell cycle-related genes and DNA damage response and repair (245, 246). These studies reflect the intrinsic heterogeneity between MSC populations in growth kinetics. Individual populations may also contain cells at different stages of differentiation and/or different proportions of highly proliferative cells. These variables have also been shown to vary from donor-to-donor (219).

Determining novel predictive biomarkers of therapeutic potency is of utmost importance before clinical usage, and viability and metabolic fitness have been recently proposed as potency qualities (184). Metabolic status is affected after long-term *in vitro* expansion, and it can reflect differential stemness behavior (242, 247–249), as well as cell immune functionality (250). Overall, considering the need to generate enough number of cells, the proliferative and metabolic characteristics of AT- and UC-MSCs may favor their use over BM-MSCs (251, 252).

### Tri-lineage Differentiation Potential

The ability to undergo *in vitro* differentiation toward mesodermal lineages is, probably, the most differential property to biologically identify MSCs (253). Several culture-differentiating conditions have been reported to demonstrate the ability of MSCs to differentiate into adipocytes, osteoblasts and chondroblasts *in vitro*. Reports on tri-lineage differentiation potential have been inconsistent across different laboratories, and this may be due to the diversity of in-house protocols, culture conditions and media supplements, or the divergence in the cell preparations (219) and *in vitro* aging (54). Moreover, studies have reported a strong “tissue memory” effect, believed to be mainly driven by epigenetic factors (252, 254, 255). For instance, BM-MSCs present enhanced osteogenic and chondrogenic differentiation while AT-MSCs are usually more readily able to exhibit adipogenic differentiation (234, 241, 251).

Conflicting data however exists surrounding PT-MSCs as they have shown a heterogeneous potential to undergo mesodermal differentiation (241, 256, 257). Kern et al. reported that CB-MSCs could not differentiate toward adipocytes, similar findings were also reported for PL-MSCs (238). Other investigators, besides confirming the low adipogenic potential of CB-MSC (258), have reported higher osteogenic (247, 259) and chondrogenic potential (257, 260). Differences in identical genetic background perinatal MSC sources have also been described, with strikingly inconsistent results reported from AM-MSCs (216). Finally, similar observations have been reported for UC-MSC, with some studies suggesting higher adipogenic and osteogenic abilities (243), whereas others stated reduced differentiation compared with adult sources (240, 245).

While the field moves toward cell-free therapies (261) and mechanisms of action are mainly driven by paracrine and immunomodulatory effects (176), assessing the degree of commitment toward mesodermal linages to determine the most effective and suitable source for cell therapy may have less relevance. However, in other circumstances understanding how these differences affect the biology of MSCs could be an attractive avenue to study biological changes occurring throughout fetal development and adulthood (258), as well as to help define therapeutic strategies where use of MSCs is heavily influenced by such differentiation, such as bone and cartilage regeneration (262, 263).

### Cell Surface Markers

MSCs are not a homogeneous population but rather an amalgamation of different subpopulations bearing different cell surface markers. Currently, a “true” marker for MSCs does not yet exist, which makes MSC identification challenging. In an attempt to unify MSC identification and characterization, in 2006 the Mesenchymal and Tissue Stem Cell Committee of the ISCT proposed a panel of minimal surface antigens to define human MSC (253). Within this criterion, they defined that at least 95% of the stromal population should express CD105, CD73, and CD90, and lack (≤2%) the expression of CD45, CD34, CD14 or CD11b, CD79alpha or CD19, and HLA class II. The negative markers are commonly used to confirm the absence of contaminant cells in MSC preparations such as hematopoietic progenitors, endothelial cells, leukocytes, and co-stimulatory molecules. The vast majority of studies have reported comparable immunophenotypic profiles that follow ISCT criteria regardless of source, although with moderate donor variability (219, 246, 264). In some cases, extended culture has been seen to reduce the expression of CD105 (54) and UC-MSCs have demonstrated lower (<95%) CD90 and CD105 expression (217).

However, the ISCT criteria do not uniquely identify stromal cells, as the proposed markers are also expressed in other connective tissue cells (265). Therefore, broader flow cytometry panels have been designed to best identify MSCs beyond the minimal criteria. Most of the protocols include the assessment of CD29, CD44, CD59, CD140b, CD166, TLR4, and PDL, commonly expressed (>95%) in human MSCs; and CD93, CD133, CD243, CD235, and SSEA1, with no or very low expression levels in human MSCs. Expression of other markers such as CD71, CD146, CD106, and CD274 has been shown to be heterogeneous, and in some cases correlates with donor age (53). Adhesion molecules such as CD44 (hyaluronic acid receptor) or CD29 (integrin β1 receptor) are highly expressed in human MSCs and have been recently proposed to be included in characterization panels (266). However, expression of markers such as CD146, another key adhesion molecule, can vary between sources, being highly expressed in UC-MSCs compared with BM-MSCs (230, 237, 240, 264) and subcutaneous AT-MSCs (267). Other markers found to be increased in UC-MSC preparations are CD10, CD49d (integrin α4), CD54 (ICAM1), (240) CD200, and PDL2, whereas CD119, IFNγR1 and CD183 (CXCR3) are under-expressed (264). An additional marker with functional relevance that has been shown to vary greatly between sources is the coagulation factor III or tissue factor (TF/CD142) (268), with increased levels being described in in AT- and PT-MSCs compared with those of BM-MSCs populations (170, 218, 269, 270).

Other researchers have investigated whether surface markers such as CD271, SUD2, MSCA1, CD34, and CD44 could serve to selectively enrich MSC populations. Differences between sources led to different selection efficiency and changes in biological properties. For instance, only CD34 was able to successfully isolate AT- and BM-MSCs, and interestingly the positive sorted populations showed greater proliferative capacity, increased osteogenic potential and HGF expression (271). Due to their perivascular origin, higher levels of CD34 and CD36 have been reported in AT-MSCs, albeit their expression decreases early after isolation (230, 234, 239). On the other hand, CD271 has been reported to be absent from MSC preparations in other studies (246, 272). Other differentially expressed markers are SSEA4 (higher in BM- and UC-MSC), MSCA1 (absent in UC-MSC, highly expressed in BM-MSC) and CD271 (high in BM-, low in AT-, absent in UC-MSC) (230, 271, 273). Nevertheless, it still remains unclear whether differences in MSC surface markers are correlated with therapeutic activity or potency (266).

### Secretome Profile

It is now well-accepted that the therapeutic effects of MSCs are primarily mediated by their ability to interact and respond to environmental stimuli releasing soluble factors and EVs (274). The ability to sense changes is also translated *in vitro*, where cell culture conditions (219) or exposure to licensing strategies (275) can impact the secretome, highlighting plasticity and ability to adapt and respond to surroundings (274, 276). The so-called MSC secretome is composed of small molecules, chemokines, cytokines, growth factors, as well as EVs (277, 278). The literature has shown striking differences in the composition of MSC-secretome depending on the cell source. Moreover, variable results between studies add to the heterogeneity, further challenging the process of deciphering “true” biological properties that relate to therapeutic actions. It also makes it challenging to choose a specific MSC source to best align with the pathophysiology of the target disease.

#### Soluble Factors

MSCs have been reported to secrete large amounts of pro-angiogenic, pro-proliferative, anti-apoptotic, anti-inflammatory, anti-fibrotic and matrix-remodeling soluble factors. Several studies have shown that perinatal sources of MSC have a more diverse and protein-abundant secretome, with a more complete pro-angiogenic array (244, 246, 256, 279). Although some studies failed to detect differences in functional studies (256, 279), others have shown *in vitro* superior abilities of UC- and BM-MSCs in inducing angiogenic phenotypes (246). UC-MSCs have also exhibited greater abilities to induce vessel-like structures than maternal sources of MSCs, through enhanced secretion of HGF and VEGF (280). However, a potential confounding factor in these studies is the combination of maternal and fetal cells within PL-MSC preparations, which could be limiting their angiogenic properties (280).

In contrast with studies reporting that AT-MSCs had a weaker angiogenic secretome, lacking central molecules such as AKT1 and FGF2 (246), others have demonstrated *in vitro* and *in vivo* angiogenic potential of AT-MSC preparations in a model of hindlimb ischemia, due to the secretion of VEGFA, TGFβ, bFGF and HGF, well-known factors of endothelial cell survival, proliferation, and migration (251, 281, 282).

The secretome of UC-MSCs has been reported to be enriched with anti-inflammatory cytokines such as IL1RA and IFNα, pro-inflammatory cytokines such as IL6 and IL8; and mitogenic factors such as HGF, TGFβ2, PDGFAA and GCSF (240). BM-MSCs, on the other hand, while secreting lower levels of IL6, IL7, IL8, and IL12, have been reported to secrete higher concentrations of PDGFBB, MCP1, SDF1, TGFβ1, and VEGF (232, 240, 251, 283), exhibiting a stronger anti-inflammatory profile that increased upon exposure to hypoxic conditions, together with the expression of other angiogenic and anti-apoptotic factors such as ANG, HIFα, MMP9 and Bcl2 (284, 285). Increased levels of VCAM1 in the BM-MSC cytokine profile have been related to better angiogenic paracrine activity (275, 286).

AT-MSCs contain large amounts of IL7 and IL12 together with several metalloproteinases (MMP1, MMP3, and MMP13) and extracellular matrix components (240). Interestingly, expression of different MMPs between AT- and BM-MSCs has been previously reported, accounting for different mechanisms to promote angiogenesis (287).

Donor-to-donor variability and heterogeneity of MSC populations make it difficult to define a “secretome profile” specific for each tissue source of MSCs. Another layer of complexity relates to the use of cell culture supplements during *in vitro* expansion containing growth factors which may also affect the secretome (246). Ultimately, dissecting the secretome of each specific MSC preparation may provide insights of their advantages in any given pathology (e.g., superior angiogenic secretome identified in BM- and UC-MSC preparations might make them an optimal source for ischemic disorders).

#### Extracellular Vesicles and miRs

In recent years, EVs have been proposed as a potential mechanism of therapeutic benefit of MSCs. EVs are lipid bilayer-delimited particles released by cells into the extracellular space carrying within them a range of cargos: subcellular components such as mitochondria, proteins, lipids, microRNAs (miRs), messenger RNAs (mRNAs) and transfer RNAs (tRNAs). Their roles have been described in multiple physiological and pathological process and are considered a mechanism of cell-to-cell signaling (288). MSCs secrete microvesicles (MVs) and exosomes, and both have been widely explored as cell-free alternatives to their cellular counterparts. Cell-free therapies, if able to recapitulate therapeutic efficacy of whole-cell preparations, offer several advantages due to a higher safety profile, lower immunogenicity, potential to bypass the lung trapping effect, and potential inability to induce neoplastic processes (289). It has also been described that EVs suppress pro-inflammatory processes, reduce oxidative stress and fibrosis in several *in vivo* models (290, 291).

Currently, there is limited data available on head-to-head comparisons of the paracrine benefits of different sources of MSC. We have only been able to identify a few studies reporting differential compositions and therapeutic effects of EVs derived from different sources. Exosomes derived from EM-MSCs enriched with miR-21 have been shown to confer superior cardioprotection after myocardial infarction over that of AT- or BM-MSC (292). Furthermore, a higher content of angiogenic-related cargos in EVs from AT-MSCs, compared to BM-MSCs, has been shown to promote wound healing (293). Similar findings have been attributed to the presence of miR-125a in AT-MSCs exosomes (294) and found to be enhanced by hypoxia priming (295). Albeit limited, recent data has described higher yields of particles secreted by AM-MSCs than BM-MSCs with similar size distribution, morphology, and immunophenotype (296).

Additional studies exploring the cargo within EV preparations from various sources have also reported beneficial effects. Exosomes secreted from BM-MSCs have been found to activate signaling pathways related to wound healing and angiogenesis (297–300) while their miRNA “repertoire” has been linked with anti-fibrotic, anti-apoptotic, pro-angiogenic and pro-proliferative properties (301) and the modulation of the native immune system (302). Exosomes originated from UC-MSCs have been found to contribute to wound healing (303) and reduce renal fibrosis after ischemic events by increasing capillarity density, reducing cell apoptosis, and restoring mitochondrial dynamics through miR-30b/c/d (116).

Despite the growing body of literature studying exosomes and their cargo in several settings, minimal evidence has been reported trying to underpin the molecular mechanism of action. Ferguson et al. investigated the biological processes modulated by exosomal miRs and found that targeted pathways were related to Wnt signaling, TGFβ and PDGF signaling, proliferation and apoptosis (301). Similarly, the expression profile of miRs in MSC-EVs derived from different sources lacks consistency. Although several studies have compared the expression between EVs and their parental MSC [reviewed by Qiu et al. (304)], limited studies have explored differences in miRs produced by different tissue-derived MSCs. To our knowledge, only one study has investigated the full RNAome derived from AT- and BM-MSC exosomes (305).

Akin to what we have described in the MSC field, EV isolation techniques lack standardization and generate variable products that can yield substantial differences. The use of serum or human platelet lysate supplements or serum-free conditions challenges the direct comparison of the relative contribution of EVs derived from MSC and other non-EVs factors. In a recent study, Whittaker et al. reported that soluble factors, that were non-EV molecules, were essential and sufficient to stimulate angiogenesis and wound healing *in vivo* (306). Their results concluded that most isolation techniques generate heterogeneous preparations containing other bioactive molecules that might mislead the attribution of therapeutic benefits.

Future studies defining the properties of miRs and exosomes will help in better understanding their biological functions and implications in cell-free therapies.

### Immunomodulatory Properties

The immune system plays a central role in tissue recovery after injury. MSCs interact extensively with the immune system and promote an anti-inflammatory and pro-regenerative environment that favors injury resolution and, ultimately, tissue repair (19, 307, 308).

Many studies have demonstrated the ability of MSCs to modulate the activation, proliferation, and function of various immune cells such as T and B lymphocytes, natural killer cells (NK), dendritic cells, macrophages, and neutrophils. Such activities rely on the plasticity (309) of MSCs to produce cytokines in response to the different stages of the inflammatory process (310) and researchers are now investigating whether MSC immunomodulatory properties are influenced by their tissue of origin. Nevertheless, results from these studies are rather diverse and it is challenging to make adequate conclusions.

Some studies have compared the immunomodulatory properties of perinatal MSC, mainly UC-MSCs and CB-MSCs, with adult tissue sources (AT- and BM-MSCs). Overall, MSCs derived from perinatal tissues have the lowest expression of HLA antigens (HLA-DMA, HLA-DPB1 and HLA-DR) and immune-related genes (JAG1, TLR4, TLR3, NOTCH2, and NOTCH3) (243), together with decreased amounts of IL1α, IL6, IL8, (244) and increased IDO, IL1β, LIF, and TNFβ2 in their secretome (311). UC-MSCs also have the most prominent inhibitory effects on T cell proliferation, in both co-culture and trans-well mixed lymphocyte reaction (MLR) *in vitro* assays, followed by PL-MSCs, AT-MSCs and BM-MSCs (243). Other studies however have shown greater inhibition of allogeneic T cell proliferation by either BM-MSCs *via* increased expression of PDL1, IL10, and TGFβ1 (230, 241), or AT-MSCs, which have been shown to secrete higher levels of IDO (243, 312).

Key adhesion molecules and other immunological markers such as CD10, CD146, CD49d, ICAM1 (CD200), and PDL2 are also increased in WJ-MSC preparations, together with decreased presence of IFNγR1, CXCR3 and other costimulatory molecules such as CD80, CD86, and CD40 (264, 313). In a recent *in vivo* study performed by Tago et al., AM-MSCs and not BM-MSCs were able to reduce local inflammation and PD1^+^CD8^+^T cell proliferation when delivered into a murine model of GvHD (314). In addition, PDL1-enriched EVs derived from UC-MSCs have been proven to be the mechanism whereby UC-MSC-EVs enhance immunosuppression (315).

In line with what has been briefly described, Mattar et al. have also highlighted the intrinsic heterogeneity of MSCs, where *in vitro* data also might not relate to the complex *in vivo* situation (316). The inflammatory context is defined by a variety of cell types and stimulating factors that are determined to influence and “license” MSCs which may adapt and change their interactions with the immune system as a result (310). Therefore, future studies should aim to decipher if similarities/disparities of *in vitro* results correlate to similar *in vivo* functions and whether biological properties can help to define cell performance, providing rationale for the use of one particular cell source for any given disease.

## Considerations for the GMP-Production of Human MSCs for Kidney Disease

**Figure 5:**
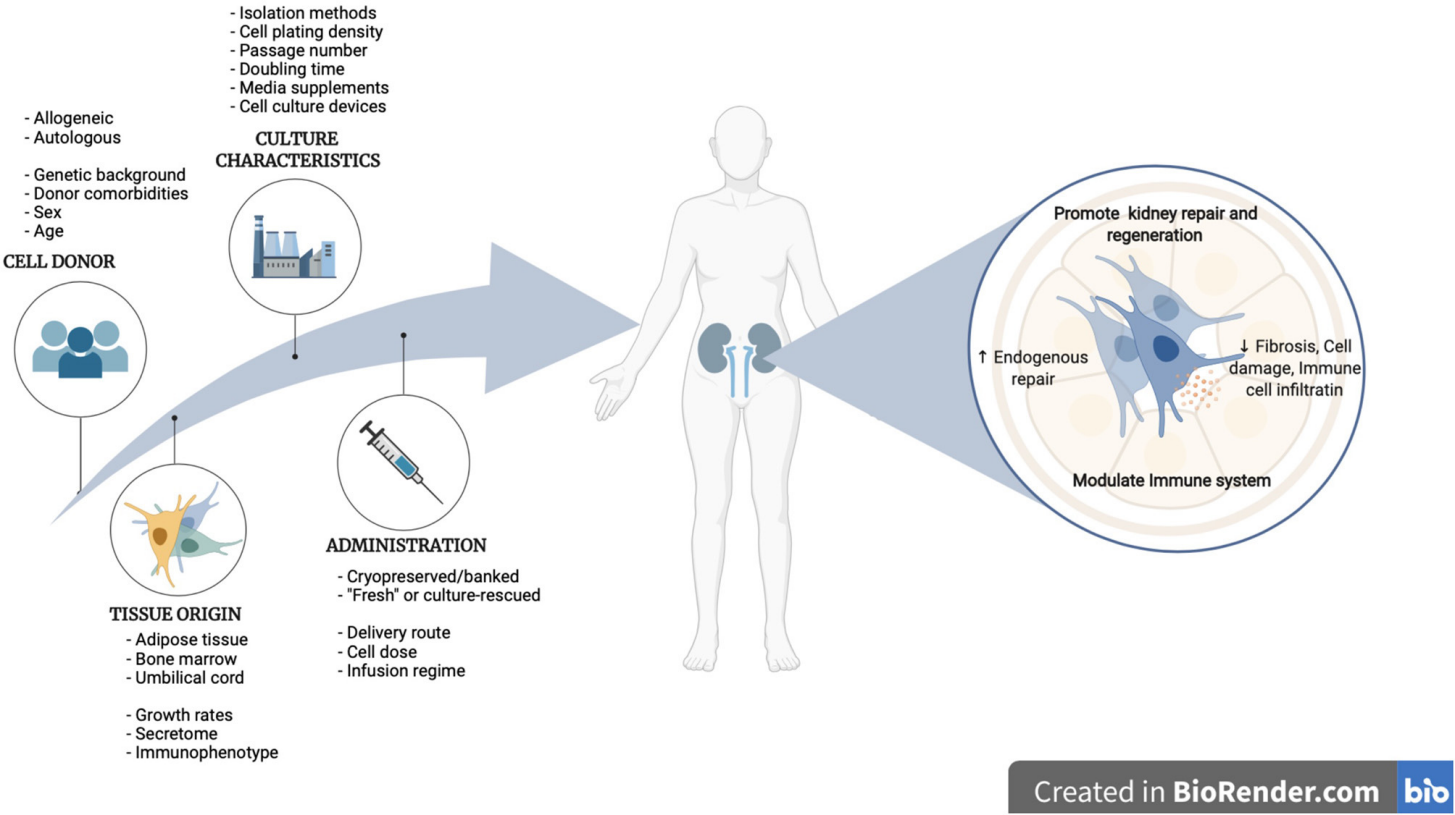
Roadmap to clinical translation of MSC therapies for kidney disease, from MSC manufacturing variables to therapeutical benefits in renal pathologies.

The regulatory agencies in the European Union (EU), United States of America (USA) and Asia, have adapted regulatory pathways to accelerate patient access to advanced therapies such as ATMPs. However, the legal frameworks for ATMPs, as well as the criteria to be met to define a product as such, differs across these regions (317–319). In Europe, MSC-based ATMPs are governed by the EU Regulation 1294/2007/EC and Directive 2009/120/EC, and its manufacturing must be compliant with European current good manufacturing practice (cGMP) guidelines (EudraLex Volume 4, Part IV). These ATMP-specific regulations have been put in place to ultimately ensure the safety and wellbeing of patients, as single alterations in the bioprocess hold the potential to alter the final product with potential risk to the patient. For this reason, in the absence of proof of product comparability, regulatory authorities are prompted to require further re-validation, which in the worst-case scenario have resulted in pre-clinical data being invalidated and clinical trial approval requiring re-authorization. Thus, optimal manufacturing variables must be considered and identified during early stages of development, as changes in the bioprocess workflow later in the translational pathway may have a significant long-term impact on the success of the therapy with time and cost consequences but also a significant time-to-market delay (320, 321). In addition, having full control of the process is crucial for ensuring consistent production and quality standards in terms of safety, identity, and potency, and this control can only be guaranteed by having in place systems of quality assurance (QA) and quality control (QC) across all stages of the bioprocess. While guidelines and some common criteria now exist for ATMP developers to follow, consistent clinical-grade production of MSCs is not yet achieved due to a lack of standardization and harmonization of manufacturing processes. Critical parameters in MSC manufacturing include the source of the starting material, and culture processing conditions, such as seeding density, passage number, media supplements and culture-expansion devices, among others (322, 323). These parameters have been shown to be highly variable among manufacturing centers and laboratories worldwide (47–49), increasing the number of variables, as illustrated in Figure 5, that should be considered when carving therapeutic approaches in specific clinical settings such as KD.

### Tissue Origin

For many years BM has been the predominant cell type in clinical trials for kidney disease (Figure 3D) and others (47, 48, 324). One of the main advantages of using BM-MSCs is the ability to use them in autologous settings without triggering anti-donor immunoreactions. However, donor-related parameters such as age, disease severity and presence of comorbidities should be considered as they have been shown to affect MSC characteristics (53, 235, 325, 326). Recent attention has been given to donor gender, as there is now increasing evidence of gender influencing MSC properties such as growth kinetics, paracrine secretion and *in vivo* therapeutic potential (52, 53, 327, 328). Another disadvantage of using BM-MSCs is the need for large amounts of raw material to allow for extensive *ex vivo* cell expansion to obtain clinical doses, as these cells constitute a rare population (only 0.001–0.1%) within the whole bone marrow fraction (329). Finally, BM collection requires an invasive bone harvest procedure, which is accompanied by pain, risk of infection and other limitations such as patient's comorbidities that can render this procedure unsuitable.

Alternative sources such as AT have been considered for many years. Subcutaneous AT has been shown to be an abundant source of AT-MSCs, with a yield of MSC precursors 500 times higher than from an equivalent amount of BM (330). AT-MSCs can be used both in autologous and allogeneic settings, which is advantageous. In addition, AT can be easily accessible as it is discarded as medical waste in many operations, which would be useful in allogeneic settings. In autologous settings, AT is harvested by less invasive procedures than BM, such as liposuction. However, like in BM sources, the therapeutic efficacy of autologous MSC therapies may be limited by the intrinsic impact that the disease, age, and gender may have on MSCs characteristics (50, 51).

Thus, in the past few years, more consideration has been given to the potential use of allogeneic MSC therapies due to the hypoimmunogeneic phenotype of these cells (331). Perinatal and birth-associated tissues have become an attractive source of allogeneic MSCs for many reasons, the main being that this material is considered medical waste and discarded every day in hospitals worldwide. Also, these MSCs are obtained from the youngest donors possible (neonates), removing donor age-related confounding effects. Perinatal tissue sources such as UC have reported isolation rates ranging from 0.2–1.8% (216), and these cells have also been shown to have significantly higher proliferation rates, compared to MSCs isolated from adult tissues (217, 237, 241).

Overall, results from our search showed that the predominant source of MSCs used in clinical trials in renal pathologies is BM (58.1%), followed by UC (23.6%) and AT (11%) (Figure 3D). Also, autologous MSC therapies are predominant (62.5%) over allogeneic. Similar trends have been also observed in other studies (47, 48, 324). Interestingly, in renal pathologies, BM-MSCs have been mostly used in allogeneic settings (59.4%), while AT-MSCs have been used mostly in an autologous manner (80%) (Figures 3Ei–iii).

### Culture Processing Characteristics

Based on ongoing clinical trial data in kidney disease, human MSCs are transplanted at typical doses of 1–2 million cells/kg and often not exceeding 10 million cells/kg (Table 2). Doing a basic dose extrapolation, for an 80 kg person the estimated human MSC doses per patient would range between 80 and 800 million cells per patient. Thus, the generation of clinical doses of MSC requires large-scale *ex vivo* cell expansion and having an optimal scale-up strategy for MSC manufacturing is critical for ensuring product quality while minimizing costs and time of production, as well as avoiding potential risks. The following are some key variables in the cell culture bioprocess to consider when designing MSC therapeutics.

#### Cell Plating Density

Cell plating density is a key parameter to ensure adequate expansion rates while maintaining stemness properties (332). The literature suggests that plating densities, both at isolation and subculturing, can influence functional and molecular characteristics of the MSCs (49, 219, 333), and yet it is something not well-standardized across laboratories. There are contradictory reports regarding the optimal subculturing seeding densities. Generally, higher plating densities (i.e., >5000 cells/cm^2^) have resulted in reduced proliferation rates, most likely due to contact inhibition by confluency and the need for continuous premature passaging (332), which is known to critically affect the proliferation rate of MSCs (242, 333–337). Also, the log phase has a longer duration in cells plated at low densities, and therefore more population doublings occur due to a longer exponential growth phase (338). Thus, finding the optimum seeding density for a maximal expansion of therapeutic MSCs while being cost-effective is crucial (339). Some studies have recommended using very low seeding densities when subculturing, as such required for clonal selection (i.e., <500 cells/cm^2^), as it has been shown to result in the highest cell proliferation rates (339–342). Other studies have used slightly higher densities between 2, 000 and 4, 000 cells/cm^2^ (49, 206, 343). The disadvantage of using very low seeding densities (i.e. <100 cells/cm^2^) for a clinical-scale production of MSCs is the large surface area required to culture therapeutic doses of MSCs, which is not feasible when using 175 cm^2^ flasks, due to the need for large incubator occupancy, a sizable amount of lab reagents and increased handling times. Plating densities of 1, 000 cells/cm^2^ have been considered reasonable, as this density still allows for a high number of harvested cells (243, 342, 344). However, often more cost/labor compromises are undertaken with most current clinical trials using plating densities of over 3, 000 cells/cm^2^ (48). In the kidney disease clinical trial arena, a mix of low and high plating densities have been reported, ranging from 100 cells/cm^2^ to 500, 000cells/cm^2^ (Table 2).

Cell plating densities at the isolation phase have also shown similar outcomes in clinical trials. Sotiropoulou et al., have shown that initial plating density of bone marrow mononuclear cells (BM-MNCs) had a great impact on the size of the MSC-enriched population derived, with the maximum number of adherent cells at P0 obtained when using lower plating densities (<25, 000 cells/cm^2^) compared to high plating densities (>50, 000 cells/cm^2^), with 1, 000 cells/cm^2^ being the optimal condition (341). But similar challenges are encountered here, where large surface areas may be needed for the initial plating. For instance, given that up to 1 × 10^8^ BM-MNC are commonly obtained, ~600 × 175 cm^2^ would be needed to seed 1 × 10^8^ BM-MNC at 1, 000 cells/cm^2^, which is not practical or cost-efficient. Indeed, the most common seeding densities used in clinical trials are 1.5–1.6 × 10^5^ cells/cm^2^, followed by 1 × 10^6^ cells/cm^2^ (48). In our search on clinical trials for kidney disease, seeding densities at isolation have been reported to be 1–2 × 10^5^ cells/cm^2^ (Table 2). One approach to reducing the plating surface area at isolation would be cell enrichment by prospective immunoselection using antibodies directed against specific cell surface markers to obtain a more homogeneous, pure, and well-defined functional subset of MSC subpopulation. For instance, some markers that have been used to purify distinct subsets of MSCs include CD146 (345), CD271 (346, 347), Stro-1 (348) and CD362 (349), which have shown properties such as having greater paracrine immunomodulatory and anti-inflammatory properties (188, 345, 349), increased osteogenic commitment (346, 347), and higher production of cardiovascular-relevant cytokine production (348).

#### Passage Number

MSCs are an adherent cell population and have normal growth inhibition when confluent. This has led to the use of successive passages for obtaining a large amount of MSCs. Passage number, which refers to the number of times cells have been sub-cultured, is often recorded as an indicator of cellular age. Cell expansion requires enzyme dissociation and cell subculture, and while the evaluation of the optimal cell confluence may vary among operators, a 70–80% confluence is recommended to be reached before detachment (323). In general, passage numbers from 1 to 5 are commonly used in clinical trials (48, 324) and we have also found similar trends in our search (Table 2). The use of low passage MSCs for therapy is currently preferred to higher passages due to the impact that extended passaging has in decreasing the cell proliferation rates and increasing senescence times (242, 333–337). Long-term culture has also been shown to affect other properties of MSC such as immunosuppressive activity (242, 335), trilineage differentiation (333, 334, 337), *in vivo* therapeutic potential (333, 337, 350), and have also been shown to increase genomic instability although not to induce *in vivo* tumorigenicity (336, 337). Also, the advantage of transplanting MSC at earlier passages over late passages was demonstrated clinically in patients with GVHD, where 1-year survival rates were higher in those patients that received MSCs at passages 1–2 (75%) compared to those that received later passage MSCs (passage 3-4) (350). Effects of passage number in combination with cryopreservation cycles have also been described to impact the safety profile of MSC products, with cells cultured for extended times triggering stronger prothrombotic events compared with cells cultured for shorter times and “fresh” cells (44, 168). For these reasons, regulatory agencies have recognized the importance of cellular age tracking during the manufacturing process, as well as the need for karyotypic analysis as a product release criterion. While passage number has been traditionally used for cellular age tracking, this is largely dependent on the specific seeding and harvest density conditions, and therefore it is challenging to make comparisons between studies. Population doubling level (PDL), which refer to the total number of times the cells have doubled during *in vitro* culture, is, therefore, a more robust and accurate parameter to define cellular aging. An upper limit of PDL, before culture ceases to replicate, must be defined for each product. The literature suggests a maximum number of cell population doubling to be between 15 and 30 (333), although this may be influenced by the cell type (i.e., UC-MSC showed higher proliferation rates and later senescence than AT- and BM-MSCs) (230, 242, 243, 351) and the culture processing characteristics (219).

#### Media Supplements

The most common basal media employed in current MSC expansion protocols are DMEM or αMEM (48), although αMEM has shown to be more suitable for both isolation and expansion of MSCs (341, 352). MSCs however require media supplements such as serum and/or growth factors to be added to the basal medium for optimal MSC growth. Most expansion protocols, especially at laboratory-scale and in early phase clinical trials, have used fetal calf serum (FCS) with 10% being the standard concentration used for MSC expansion (48). Basic fibroblast growth factor (b-FGF), at a final concentration of 1-2 ng/ml, is also added to the basal media to enhance the proliferation rate of cultured cells while maintaining the multilineage differentiation potential (353, 354). Nevertheless, the use of FCS for large-scale production of clinical doses of MSCs is not a viable option (355). Limitations in the availability of the raw material are a major cost driver and represent a current bottleneck. Also, due to the batch-to-batch variability, FCS lots must be carefully tested to ensure optimal MSC expansion rates and tri-lineage differentiation potential. In addition to this, there are current regulatory challenges associated with the use of FCS to produce clinical-grade MSCs due to the risk of inter-species cross-contamination, and regulators urge the development of xenogeneic-free compositions. Considerations to using human-derived blood components such as human platelet lysate (hPL), often at a final concentration of 5–10%, have been given (47, 356). While hPL has been shown to have comparable growth factors and cytokines to FCS to support MSC growth (355), it poses some important challenges (357). HPL can be derived from autologous collections, but it does not represent a good commercial model. Large scale, allogeneic, off-the-shelf pools are easier to standardize, have less lot-to-lot variation, is more economical to produce and therefore represents a better commercial model. Nevertheless, while hPL collections can be obtained from up to 100 different individuals, the size of pools is a current issue and a topic of debate. Recently, regulators have expressed their concern about the increased risk of transmission of infectious agents in large pooled hPL products. The *European Pharmacopeia* have recommended the limitation of pooled donations unless sufficient methods for inactivation or removal of viruses are applied during the production, although it does not give specific recommendations to the pool size (Chapter 5.2.12). However, representatives of the German Federal Regulatory Authority specified the restriction to a maximum of 16 donors (358). This imposes many challenges for ATMP developers and commercial entities, who must fast adapt their products to the constantly evolving regulatory framework. Overall, a consensus is needed to ensure the quality and safety of hPL supplements regarding the source of platelet concentrate, donor- and lot- variability, manufacturing processes and minimum release criteria (357).

Due to the concerns mentioned above, the development of new xenogeneic-free, chemically defined formulations is urgently needed. Chemically defined media (CDM) are generally composed of basal media to which supplements of known composition (i.e., growth factors, hormones, attachment factors, binding proteins, and vitamins) are added (320). An ideal MSC media should contain chemically defined constituents preferably of recombinant human origin that support the isolation and culture expansion of human MSCs obtained from different tissue sources while maintaining MSC phenotypic characteristics, morphology, and mechanism of functional benefit. Ideally, it should also support the attachment of MSCs without coating. Extensive testing is however required to ensure these media fulfill MSC requirements, but when successful, this type of media will have the potential to enhance batch-to-batch consistency in the cell manufacturing process and will therefore represent a more cost-effective and risk-reduced approach. To date, 10% FCS continues to be the most common media supplement employed in clinical trials for kidney disease, although some consideration has been given to the use of xenogeneic-free media such as hPL (5 or 10%), human serum albumin (HSA) and a CDM (Table 2). Currently, some commercial and non-commercial CDM formulations have been investigated (359–363), however, there is still limited availability of some of these media for large-scale manufacturing at GMP quality level.

#### Cell Culture Devices

Currently, a complete closed system that allows MSC acquisition, expansion, and delivery at the bedside, is not yet available. GMP conditions have been mainly achieved using laminar airflow cabinets to perform the main steps in the bioprocess such as culture inception, medium changes, subculturing and packaging. Traditionally, scale-out of MSC manufacturing has been achieved using 2D monolayer cultures using multilayered flasks (Corning® CellSTACK and Nunc^TM^ Cell Factory^TM^) of 1 to 40 levels, and surfaces ranging from 636 cm to 25, 440 cm. However, this is not an optimal system for large-scale production of therapeutic doses, as it is labor-intense, requires significant manual handling, and is not cost-effective (320, 364). These are also static systems, which lack real-time process monitoring of culture conditions, and are more susceptible to batch-to-batch variation due to a non-homogeneous environment within layers (320). Alternatively, GMP-compliant, closed, automated, high-volume cell expansion systems offer great advantages, as they enable the real-time monitoring of process variables such as pH, pO_2_, pCO_2_, metabolite accumulation or presence of contaminants, and hence it guarantees a homogeneous distribution of culture environment and ensures a culture process under well-controlled and reproducible conditions and production of quality MSCs for clinical use. A variety of bioreactors are available including stirred tank bioreactors with microcarriers (365, 366), rocking motion (367), disposable fixed bed (368), and hollow fiber-based continuous perfusion bioreactors (369, 370). The surface area from these bioreactors ranges from 21, 000 cm^2^/unit to up to 3, 750, 000 cm^2^/unit, and they all offer distinct advantages and limitations that must be considered (320, 364, 371). In our search, an overwhelming majority of clinical trials have used 2D culture conditions, with only 1 study considering a 3D bioreactor, the Quantum Cell Expansion System (Terumo BCT) (NEPHSTROM clinical trial, NCT02585622) (Table 2).

## Conclusions and Future Perspectives

While the field of cell-based therapies evolves, the selection of particular MSC types in specific clinical conditions remains to be elucidated. In the past decades, BM has been the preferred source of MSCs used in clinical trials of kidney disease, but recently allogeneic sources have emerged as strong candidates in the clinical research arena. Ideally an allogeneic, “off-the-shelf” MSC product would be preferred, especially for acute kidney disease settings where delivery time is crucial. We hypothesize that, the use of MSCs may be rationalized by the intrinsic origin-specific properties which may make one cell type more advantageous for a specific disease condition. Overall, the high proliferative capacity, the stronger immunosuppressive effects and hypo-immunogenetic properties of UC-MSCs paired with their allogeneic nature makes them ideal to be used in an “off-the-shelf,” large-scale, universal production model. Although it is likely that the choice of MSC type may be driven by intellectual and/or industrial property on isolation methods, protocols and/or reagents in addition to issues of functional and biological superiority, considerations should be also given to the safety of this therapies, in particular accounting for differences in immune and hemocompatibility characteristics. Considering this constellation of variables, robust clinical guidelines and well-characterized therapeutic products are urgently needed to deliver safer, effective, and potent MSC therapies to improve clinical outcomes. This will require a greater understanding of the biology of MSCs from different tissue sources along with an alignment with disease pathophysiology coupled with consideration and standardization of the cell manufacturing variables reviewed in this article.

## Author Contributions

SC-C, CS-N, and TO'B wrote sections of the manuscript. All authors contributed to manuscript revision, read, and approved the submitted version.

## Funding

This work was supported by grants from SFI CÚRAM Research Center (Grant No. 13/RC/2073), Science Foundation Ireland (SFI) (Grant No. 18/IF/6252) and from the European Union's Horizon 2020 research and innovation programme under the Marie Skłodowska-Curie Grant Agreement No. 813839.

## Conflict of Interest

TO'B is a founder, director and equity holder in Orbsen Therapeutics Ltd. The remaining authors declare that the research was conducted in the absence of any commercial or financial relationships that could be construed as a potential conflict of interest.

## Publisher's Note

All claims expressed in this article are solely those of the authors and do not necessarily represent those of their affiliated organizations, or those of the publisher, the editors and the reviewers. Any product that may be evaluated in this article, or claim that may be made by its manufacturer, is not guaranteed or endorsed by the publisher.

## References

[1] WHO. Global Health Estimates. (2019). Available online at: https://www.who.int/data/global-health-estimates (accessed July 19, 2021).

[2] Hill NR Fatoba ST Oke JL Hirst JA O'Callaghan CA Lasserson DS. Global prevalence of chronic kidney disease – a systematic review and meta-analysis. PLoS ONE. (2016) 11:e0158765. 10.1371/journal.pone.0158765 27383068 PMC4934905

[3] GBD Chronic Kidney Disease Collaboration: Bikbov B Purcell CA Levey AS Smith M Abdoli A Abebe M. Global, regional, and national burden of chronic kidney disease, 1990–2017: a systematic analysis for the Global Burden of Disease Study 2017. Lancet. (2020) 395:709–33. 10.1016/s0140-6736(20)30045-3 32061315 PMC7049905

[4] Kidney Disease: Improving Global Outcomes (KDIGO) CKD Work Group. KDIGO 2012 clinical practice guideline for the evaluation and management of chronic kidney disease. Kidney. (2013) 3:1–150. 10.1038/kisup.2012.73

[5] Khwaja A. KDIGO clinical practice guidelines for acute kidney injury. Nephron Clin Pract. (2012) 120:c179–84. 10.1159/000339789 22890468

[6] Wang LL Janes ME Kumbhojkar N Kapate N Clegg JR Prakash S. Cell therapies in the clinic. Bioeng Transl Med. (2021) 6:e10214. 10.1002/btm2.10214 34027097 PMC8126820

[7] Heathman TR Nienow AW McCall MJ Coopman K Kara B Hewitt CJ. The translation of cell-based therapies: clinical landscape and manufacturing challenges. Regen Med. (2015) 10:49–64. 10.2217/rme.14.73 25562352

[8] Roemhild A Otto NM Moll G Abou-El-Enein M Kaiser D Bold G. Regulatory T cells for minimising immune suppression in kidney transplantation: phase I/IIa clinical trial. BMJ. (2020) 371:m3734. 10.1136/bmj.m3734 33087345 PMC7576328

[9] Sawitzki B Harden PN Reinke P Moreau A Hutchinson JA Game DS. Regulatory cell therapy in kidney transplantation (The ONE Study): a harmonised design and analysis of seven non-randomised, single-arm, phase 1/2A trials. Lancet Lond Engl. (2020) 395:1627–39. 10.1016/s0140-6736(20)30167-7 32446407 PMC7613154

[10] Griffin TP Martin WP Islam N O'Brien T Griffin MD. The promise of mesenchymal stem cell therapy for diabetic kidney disease. Curr Diabetes Rep. (2016) 16:42. 10.1007/s11892-016-0734-6 27007719

[11] Zhu X Lerman A Lerman LO. Concise review: mesenchymal stem cell treatment for ischemic kidney disease. Stem Cells. (2013) 31:1731–6. 10.1002/stem.1449 23766020 PMC3795813

[12] Morigi M Imberti B Zoja C Corna D Tomasoni S Abbate M. Mesenchymal stem cells are renotropic, helping to repair the kidney and improve function in acute renal failure. J Am Soc Nephrol. (2004) 15:1794–804. 10.1097/01.asn.0000128974.07460.34 15213267

[13] Papazova DA Oosterhuis NR Gremmels H Koppen A van Joles JA Verhaar MC. Cell-based therapies for experimental chronic kidney disease: a systematic review and meta-analysis. Dis Model Mech. (2015) 8:281–93. 10.1242/dmm.017699 25633980 PMC4348565

[14] Gooch A Doty J Flores J Swenson L Töegel FE Reiss GR. Initial report on a phase I clinical trial: prevention and treatment of post- operative Acute Kidney Injury with allogeneic Mesenchymal Stem Cells in patients who require on-pump ca. Cell Therap Transpl. (2008) 1:31–5. 10.3205/ctt-2008-en-000028.01

[15] Tögel FE Westenfelder C. Mesenchymal stem cells: a new therapeutic tool for AKI. Nat Rev Nephrol. (2010) 6:179–83. 10.1038/nrneph.2009.229 20186233

[16] Tögel FE Westenfelder C. Kidney protection and regeneration following acute injury: progress through stem cell therapy. Am J Kidney Dis. (2012) 60:1012–22. 10.1053/j.ajkd.2012.08.034 23036928

[17] Westenfelder C Togel FE. Protective actions of administered mesenchymal stem cells in acute kidney injury: relevance to clinical trials. Kidney Int Suppl. (2011) 1:103–6. 10.1038/kisup.2011.24 25018910 PMC4089688

[18] Swaminathan M Stafford-Smith M Chertow GM Warnock DG Paragamian V Brenner RM. Allogeneic mesenchymal stem cells for treatment of AKI after cardiac surgery. J Am Soc Nephrol. (2018) 29:260–7. 10.1681/asn.2016101150 29038286 PMC5748899

[19] Perico N Casiraghi F Remuzzi G. Clinical translation of mesenchymal stromal cell therapies in nephrology. J Am Soc Nephrol. (2018) 29:362–75. 10.1681/asn.2017070781 29191959 PMC5791082

[20] McIntyre LA Stewart DJ Mei SHJ Courtman D Watpool I Granton J. Cellular immunotherapy for septic shock. A phase I clinical trial. Am J Resp Crit Care. (2018) 197:337–47. 10.1164/rccm.201705-1006oc 28960096

[21] Makhlough A Shekarchian S Moghadasali R Einollahi B Hosseini SE Jaroughi N. Safety and tolerability of autologous bone marrow mesenchymal stromal cells in ADPKD patients. Stem Cell Res Ther. (2017) 8:116. 10.1186/s13287-017-0557-7 28535817 PMC5442691

[22] Saad A Dietz AB Herrmann SMS Hickson LJ Glockner JF McKusick MA. Autologous mesenchymal stem cells increase cortical perfusion in renovascular disease. J Am Soc Nephrol. (2017) 28:2777–85. 10.1681/asn.2017020151 28461553 PMC5576946

[23] Packham DK Fraser IR Kerr PG Segal KR. Allogeneic Mesenchymal Precursor Cells (MPC) in diabetic nephropathy: a randomized, placebo-controlled, dose escalation study. Ebiomedicine. (2016) 12:263–9. 10.1016/j.ebiom.2016.09.011 27743903 PMC5078602

[24] Liang J Zhang H Hua B Wang H Lu L Shi S. Allogenic mesenchymal stem cells transplantation in refractory systemic lupus erythematosus: a pilot clinical study. Ann Rheum Dis. (2010) 69:1423. 10.1136/ard.2009.123463 20650877

[25] Sun L Wang D Liang J Zhang H Feng X Wang H. Umbilical cord mesenchymal stem cell transplantation in severe and refractory systemic lupus erythematosus. Arthritis Rheum. (2010) 62:2467–5. 10.1002/art.27548 20506343

[26] Wang D Zhang H Liang J Li X Feng X Wang H. Allogeneic mesenchymal stem cell transplantation in severe and refractory systemic lupus erythematosus: 4 years of experience. Cell Transplant. (2012) 22:2267–77. 10.3727/096368911x582769c 24388428 PMC11849135

[27] Wang D Li J Zhang Y Zhang M Chen J Li X. Umbilical cord mesenchymal stem cell transplantation in active and refractory systemic lupus erythematosus: a multicenter clinical study. Arthritis Res Ther. (2014) 16:R79. 10.1186/ar4520 24661633 PMC4060570

[28] Deng D Zhang P Guo Y Lim TO. A randomised double-blind, placebo-controlled trial of allogeneic umbilical cord-derived mesenchymal stem cell for lupus nephritis. Ann Rheum Dis. (2017) 76:1436. 10.1136/annrheumdis-2017-211073 28478399

[29] Choi CB Lee TY Kim KS Bae SC. AB0370 Safety of CS20AT04, a haploidentical allogeneic bone marrow-derived mesenchymal stem cells, in a phase 1 study in lupus nephritis. Ann Rheum Dis. (2020) 79:1485. 10.1136/annrheumdis-2020-eular.328732719042

[30] Tan J Wu W Xu X Liao L Zheng F Messinger S. Induction therapy with autologous mesenchymal stem cells in living-related kidney transplants: a randomized controlled trial. JAMA. (2012) 307:1169–77. 10.1001/jama.2012.316 22436957

[31] Reinders MEJ de Fijter JW Roelofs H Bajema IM de Vries DK Schaapherder AF. Autologous bone marrow-derived mesenchymal stromal cells for the treatment of allograft rejection after renal transplantation: results of a Phase I Study. Stem Cell Transl Med. (2013) 2:107–11. 10.5966/sctm.2012-0114 23349326 PMC3659754

[32] Perico N Casiraghi F Introna M Gotti E Todeschini M Cavinato RA. Autologous mesenchymal stromal cells and kidney transplantation: a pilot study of safety and clinical feasibility. Clin J Am Soc Nephro. (2011) 6:412–2. 10.2215/cjn.04950610 20930086 PMC3052234

[33] Perico N Casiraghi F Gotti E Introna M Todeschini M Cavinato RA. Mesenchymal stromal cells and kidney transplantation: pretransplant infusion protects from graft dysfunction while fostering immunoregulation. Transplant Int. (2013) 26:867–78. 10.1111/tri.12132 23738760

[34] Perico N Casiraghi F Todeschini M Cortinovis M Gotti E Portalupi V. Long-term clinical and immunological profile of kidney transplant patients given mesenchymal stromal cell immunotherapy. Front Immunol. (2018) 9:1359. 10.3389/fimmu.2018.01359 29963053 PMC6014158

[35] Peng Y Ke M Xu L Liu L Chen X Xia W. Donor-derived mesenchymal stem cells combined with low-dose tacrolimus prevent acute rejection after renal transplantation. Transplant J. (2013) 95:161–8. 10.1097/tp.0b013e3182754c53 23263506

[36] Sun Q Huang Z Han F Zhao M Cao R Zhao D. Allogeneic mesenchymal stem cells as induction therapy are safe and feasible in renal allografts: pilot results of a multicenter randomized controlled trial. J Transl Med. (2018) 16:52. 10.1186/s12967-018-1422-x 29514693 PMC5842532

[37] Sun Q Hong L Huang Z Na N Hua X Peng Y. Allogeneic mesenchymal stem cell as induction therapy to prevent both delayed graft function and acute rejection in deceased donor renal transplantation: study protocol for a randomized controlled trial. Trials. (2017) 18:545. 10.1186/s13063-017-2291-y 29145879 PMC5689202

[38] Mudrabettu C Kumar V Rakha A Yadav AK Ramachandran R Kanwar DB. Safety and efficacy of autologous mesenchymal stromal cells transplantation in patients undergoing living donor kidney transplantation: a pilot study. Nephrology. (2015) 20:25–33. 10.1111/nep.12338 25230334

[39] Rakha A Todeschini M Casiraghi F. Assessment of anti-donor T cell proliferation and cytotoxic T lymphocyte-mediated lympholysis in living donor kidney transplant patients. Methods Mol Biol. (2014) 1213:355–64. 10.1007/978-1-4939-1453-1_29 25173397

[40] Reinders MEJ Dreyer GJ Bank JR Roelofs H Heidt S Roelen DL. Safety of allogeneic bone marrow derived mesenchymal stromal cell therapy in renal transplant recipients: the Neptune study. J Transl Med. (2015) 13:344. 10.1186/s12967-015-0700-0 26537851 PMC4632480

[41] Reinders ME Bank JR Dreyer GJ Roelofs H Heidt S Roelen DL. Autologous bone marrow derived mesenchymal stromal cell therapy in combination with Everolimus to preserve renal structure and function in renal transplant recipients. J Transl Med. (2014) 12:331. 10.1186/s12967-014-0331-x 25491391 PMC4273432

[42] Detry O Delbouille M-H Lechanteur C Somja J Roover AD Weekers L. Infusion of Third-party Mesenchymal stem Cells (MSC) after kidney and liver transplantation: a phase I-II, open-label, clinical study. Soc Francophone Transpl. (2013). Available online at: http://hdl.handle.net/2268/152646

[43] Sanz-Nogués C O'Brien T. Current good manufacturing practice considerations for mesenchymal stromal cells as therapeutic agents. Biomaterials Biosyst. (2021) 2:100018. 10.1016/j.bbiosy.2021.100018 PMC9934414 36824657

[44] Moll G Ankrum JA Kamhieh-Milz J Bieback K Ringdén O Volk H-D. Intravascular mesenchymal stromal/stem cell therapy product diversification: time for new clinical guidelines. Trends Mol Med. (2019) 25:149–63. 10.1016/j.molmed.2018.12.006 30711482

[45] Caplan H Olson SD Kumar A George M Prabhakara KS Wenzel P. Mesenchymal stromal cell therapeutic delivery: translational challenges to clinical application. Front Immunol. (2019) 10:1645. 10.3389/fimmu.2019.01645 31417542 PMC6685059

[46] Yin JQ Zhu J Ankrum JA. Manufacturing of primed mesenchymal stromal cells for therapy. Nat Biomed Eng. (2019) 3:90–104. 10.1038/s41551-018-0325-8 30944433

[47] Trento C Bernardo ME Nagler A Kuçi S Bornhäuser M Köhl U. Manufacturing mesenchymal stromal cells for the treatment of graft-versus-host disease: a survey among centers affiliated with the European society for blood and marrow transplantation. Biol Blood Marrow Treat. (2018) 24:2365–70. 10.1016/j.bbmt.2018.07.015 30031938 PMC6299357

[48] Ikebe C Suzuki K. Mesenchymal stem cells for regenerative therapy: optimization of cell preparation protocols. Biomed Res Int. (2014) 2014:951512. 10.1155/2014/951512 24511552 PMC3912818

[49] Stroncek DF Jin P McKenna DH Takanashi M Fontaine MJ Pati S. Human Mesenchymal Stromal Cell (MSC) characteristics vary among laboratories when manufactured from the same source material: a report by the cellular therapy team of the Biomedical Excellence for Safer Transfusion (BEST) collaborative. Front Cell Dev Biol. (2020) 8:458. 10.3389/fcell.2020.00458 32612991 PMC7308721

[50] Liu M Lei H Dong P Fu X Yang Z Yang Y. Adipose-derived mesenchymal stem cells from the elderly exhibit decreased migration and differentiation abilities with senescent properties. Cell Transplant. (2017) 26:1505–19. 10.1177/0963689717721221 29113467 PMC5680952

[51] Choudhery MS Badowski M Muise A Pierce J Harris DT. Donor age negatively impacts adipose tissue-derived mesenchymal stem cell expansion and differentiation. J Transl Med. (2014) 12:8. 10.1186/1479-5876-12-8 24397850 PMC3895760

[52] Crisostomo PR Markel TA Wang M Lahm T Lillemoe KD Meldrum DR. In the adult mesenchymal stem cell population, source gender is a biologically relevant aspect of protective power. Surgery. (2007) 142:215–21. 10.1016/j.surg.2007.04.013 17689688

[53] Siegel G Kluba T Hermanutz-Klein U Bieback K Northoff H Schäfer R. Phenotype, donor age and gender affect function of human bone marrow-derived mesenchymal stromal cells. BMC Med. (2013) 11:146. 10.1186/1741-7015-11-146 23758701 PMC3694028

[54] Andrzejewska A Catar R Schoon J Qazi TH Sass FA Jacobi D. Multi-parameter analysis of biobanked human bone marrow stromal cells shows little influence for donor age and mild comorbidities on phenotypic and functional properties. Front Immunol. (2019) 10:2474. 10.3389/fimmu.2019.02474 31781089 PMC6857652

[55] Lalu MM McIntyre L Pugliese C Fergusson D Winston BW Marshall JC. Safety of Cell Therapy with Mesenchymal Stromal Cells (SafeCell): a systematic review and meta-analysis of clinical trials. PLoS ONE. (2012) 7:e47559. 10.1371/journal.pone.0047559 23133515 PMC3485008

[56] Kot M Baj-Krzyworzeka M Szatanek R Musiał-Wysocka A Suda-Szczurek M Majka M. The Importance of HLA Assessment in “Off-the-Shelf” allogeneic mesenchymal stem cells based-therapies. Int J Mol Sci. (2019) 20:5680. 10.3390/ijms20225680 31766164 PMC6888380

[57] Friedenstein AJ Chailakhjan RK Lalykina KS. The development of fibroblast colonies in monolayer cultures of guinea-pig bone marrow and spleen cells. Cell Proliferat. (1970) 3:393–403. 10.1111/j.1365-2184.1970.tb00347.x 5523063

[58] Friedenstein AJ Petrakova KV Kurolesova AI Frolova GP. Heterotopic transplants of bone marrow. Transplantation. (1968) 6:230–47. 10.1097/00007890-196803000-00009 5654088

[59] Pittenger MF Mackay AM Beck SC Jaiswal RK Douglas R Mosca JD. Multilineage potential of adult human mesenchymal stem cells. Science. (1999) 284:143–7. 10.1126/science.284.5411.143 10102814

[60] Prockop DJ. Marrow stromal cells as stem cells for nonhematopoietic tissues. Science. (1997) 276:71–4. 10.1126/science.276.5309.71 9082988

[61] Murray IR West CC Hardy WR James AW Park TS Nguyen A. Natural history of mesenchymal stem cells, from vessel walls to culture vessels. Cell Mol Life Sci. (2013) 71:1353–74. 10.1007/s00018-013-1462-6 24158496 PMC11113613

[62] Mosna F Sensebé L Krampera M. Human bone marrow and adipose tissue mesenchymal stem cells: a user's guide. Stem Cells Dev. (2010) 19:1449–70. 10.1089/scd.2010.0140 20486777

[63] Crisan M Yap S Casteilla L Chen C-W Corselli M Park TS. A perivascular origin for mesenchymal stem cells in multiple human organs. Cell Stem Cell. (2008) 3:301–13. 10.1016/j.stem.2008.07.003 18786417

[64] Bianco P. “Mesenchymal” stem cells. Annu Rev Cell Dev Bi. (2014) 30:677–704. 10.1146/annurev-cellbio-100913-013132 25150008

[65] Zuk PA Zhu M Mizuno H Huang J Futrell JW Katz AJ. Multilineage cells from human adipose tissue: implications for cell-based therapies. Tissue Eng. (2001) 7:211–28. 10.1089/107632701300062859 11304456

[66] Gronthos S Brahim J Li W Fisher LW Cherman N Boyde A. Stem cell properties of human dental pulp stem cells. J Dent Res. (2002) 81:531–5. 10.1177/154405910208100806 12147742

[67] Huang GT-J Gronthos S Shi S. Mesenchymal stem cells derived from dental tissues vs. those from other sources: their biology and role in regenerative medicine. J Dent Res. (2009) 88:792–806. 10.1177/0022034509340867 19767575 PMC2830488

[68] Schüring AN Schulte N Kelsch R Röpke A Kiesel L Götte M. Characterization of endometrial mesenchymal stem-like cells obtained by endometrial biopsy during routine diagnostics. Fertil Steril. (2011) 95:423–6. 10.1016/j.fertnstert.2010.08.035 20864098

[69] Meng X Ichim TE Zhong J Rogers A Yin Z Jackson J. Endometrial regenerative cells: a novel stem cell population. J Transl Med. (2007) 5:57. 10.1186/1479-5876-5-57 18005405 PMC2212625

[70] Allickson JG. Recent studies assessing the proliferative capability of a novel adult stem cell identified in menstrual blood. Open Stem Cell J. (2011) 3:4–10. 10.2174/1876893801103010004 21686032 PMC3113522

[71] Zvaifler NJ Marinova-Mutafchieva L Adams G Edwards CJ Moss J Burger JA. Mesenchymal precursor cells in the blood of normal individuals. Arthritis Res Ther. (2000) 2:477. 10.1186/ar130 11056678 PMC17820

[72] Kadir RA Ariffin SHZ Wahab RMA Kermani S Senafi S. Characterization of mononucleated human peripheral blood cells. Sci World J. (2012) 2012:843843. 10.1100/2012/843843 22666162 PMC3354670

[73] Erices A Conget P Minguell JJ. Mesenchymal progenitor cells in human umbilical cord blood. Brit J Haematol. (2000) 109:235–42. 10.1046/j.1365-2141.2000.01986.x 10848804

[74] Anker PS in ‘t Scherjon SA der Keur CK Noort WA Claas FHJ Willemze R. Amniotic fluid as a novel source of mesenchymal stem cells for therapeutic transplantation. Blood. (2003) 102:1548–9. 10.1182/blood-2003-04-1291 12900350

[75] Wang H-S Hung S-C Peng S-T Huang C-C Wei H-M Guo Y-J. Mesenchymal stem cells in the Wharton's Jelly of the Human Umbilical Cord. Stem Cells. (2004) 22:1330–7. 10.1634/stemcells.2004-0013 15579650

[76] Miao Z Jin J Chen L Zhu J Huang W Zhao J. Isolation of mesenchymal stem cells from human placenta: comparison with human bone marrow mesenchymal stem cells. Cell Biol Int. (2006) 30:681–7. 10.1016/j.cellbi.2006.03.009 16870478

[77] Kim J Kang HM Kim H Kim MR Kwon HC Gye MC. *Ex vivo* characteristics of human amniotic membrane-derived stem cells. Cloning Stem Cells. (2007) 9:581–94. 10.1089/clo.2007.0027 18154518

[78] Tsai M Lee J Chang Y Hwang S. Isolation of human multipotent mesenchymal stem cells from second-trimester amniotic fluid using a novel two-stage culture protocol. Hum Reprod. (2004) 19:1450–6. 10.1093/humrep/deh279 15105397

[79] Tögel F Hu Z Weiss K Isaac J Lange C Westenfelder C. Administered mesenchymal stem cells protect against ischemic acute renal failure through differentiation-independent mechanisms. Am J Physiol. (2005) 289:F31–42. 10.1152/ajprenal.00007.2005 15713913

[80] Crespo-Diaz R Behfar A Butler GW Padley DJ Sarr MG Bartunek J. Platelet lysate consisting of a natural repair proteome supports human mesenchymal stem cell proliferation and chromosomal stability. Cell Transplant. (2011) 20:797–812. 10.3727/096368910x543376 21092406

[81] Detry O Vandermeulen M Delbouille M-H Somja J Bletard N Briquet A. Infusion of mesenchymal stromal cells after deceased liver transplantation: a phase I–II, open-label, clinical study. J Hepatol. (2017) 67:47–55. 10.1016/j.jhep.2017.03.001 28284916

[82] Ferenbach DA Bonventre JV. Acute kidney injury and chronic kidney disease: from the laboratory to the clinic. Néphrol Thérap. (2016) 12:S41–8. 10.1016/j.nephro.2016.02.005 26972097 PMC5475438

[83] Ishani A Xue JL Himmelfarb J Eggers PW Kimmel PL Molitoris BA. Acute kidney injury increases risk of ESRD among elderly. J Am Soc Nephrol. (2009) 20:223–8. 10.1681/asn.2007080837 19020007 PMC2615732

[84] Levey AS James MT. Acute kidney injury. Ann Intern Med. (2017) 167:ITC66. 10.7326/aitc201711070 29114754

[85] Bellomo R Kellum JA Ronco C. Acute kidney injury. Lancet. (2012) 380:756–66. 10.1016/s0140-6736(11)61454-2 22617274

[86] Wasung ME Chawla LS Madero M. Biomarkers of renal function, which and when? Clin Chim Acta. (2015) 438:350–7. 10.1016/j.cca.2014.08.039 25195004

[87] Miller RP Tadagavadi RK Ramesh G Reeves WB. Mechanisms of cisplatin nephrotoxicity. Toxins. (2010) 2:2490–518. 10.3390/toxins2112490 22069563 PMC3153174

[88] Kosieradzki M Rowiński W. Ischemia/reperfusion injury in kidney transplantation: mechanisms and prevention. Transplant Prevt. (2008) 40:3279–88. 10.1016/j.transproceed.2008.10.004 19100373

[89] Malek M Nematbakhsh M. Renal ischemia/reperfusion injury; from pathophysiology to treatment. J Ren Inj Prev. (2015) 4:20–7. 10.12861/jrip.2015.06 26060833 PMC4459724

[90] Ferenbach DA Bonventre JV. Mechanisms of maladaptive repair after AKI leading to accelerated kidney ageing and CKD. Nat Rev Nephrol. (2015) 11:264–76. 10.1038/nrneph.2015.3 25643664 PMC4412815

[91] Lange C Tögel F Ittrich H Clayton F Nolte-Ernsting C Zander AR. Administered mesenchymal stem cells enhance recovery from ischemia/reperfusion-induced acute renal failure in rats. Kidney Int. (2005) 68:1613–7. 10.1111/j.1523-1755.2005.00573.x 16164638

[92] Geng Y Zhang L Fu B Zhang J Hong Q Hu J. Mesenchymal stem cells ameliorate rhabdomyolysis-induced acute kidney injury via the activation of M2 macrophages. Stem Cell Res Ther. (2014) 5:80. 10.1186/scrt469 24961539 PMC4230233

[93] Collino F Bruno S Incarnato D Dettori D Neri F Provero P. AKI recovery induced by mesenchymal stromal cell-derived extracellular vesicles carrying MicroRNAs. J Am Soc Nephrol. (2015) 26:2349–60. 10.1681/asn.2014070710 25901032 PMC4587694

[94] Bi B Schmitt R Israilova M Nishio H Cantley LG. Stromal cells protect against acute tubular injury via an endocrine effect. J Am Soc Nephrol. (2007) 18:2486–96. 10.1681/asn.2007020140 17656474

[95] Li L Wang R Jia Y Rong R Xu M Zhu T. Exosomes derived from mesenchymal stem cells ameliorate renal ischemic-reperfusion injury through inhibiting inflammation and cell apoptosis. Front Med. (2019) 6:269. 10.3389/fmed.2019.00269 31867333 PMC6907421

[96] He J Wang Y Sun S Yu M Wang C Pei X. Bone marrow stem cells-derived microvesicles protect against renal injury in the mouse remnant kidney model. Nephrology. (2012) 17:493–500. 10.1111/j.1440-1797.2012.01589.x 22369283

[97] Yasuda K Ozaki T Saka Y Yamamoto T Gotoh M Ito Y. Autologous cell therapy for cisplatin-induced acute kidney injury by using non-expanded adipose tissue-derived cells. Cytotherapy. (2012) 14:1089–100. 10.3109/14653249.2012.693157 22731757

[98] Chen Y Tang X Li P Zhou Y Xue T Liu J. Bone marrow derived mesenchymal stromal cells ameliorate ischemia/reperfusion injury-induced acute kidney injury in rats via secreting tumor necrosis factor-inducible gene 6 protein. Biomed Res Int. (2019) 2019:1–12. 10.1155/2019/9845709 30984789 PMC6432703

[99] Zhu G Pei L Lin F Yin H Li X He W. Exosomes from human-bone-marrow-derived mesenchymal stem cells protect against renal ischemia/reperfusion injury via transferring miR-199a-3p. J Cell Physiol. (2019) 234:23736–49. 10.1002/jcp.28941 31180587

[100] Chen Y-T Sun C-K Lin Y-C Chang L-T Chen Y-L Tsai T-H. Adipose-derived mesenchymal stem cell protects kidneys against ischemia-reperfusion injury through suppressing oxidative stress and inflammatory reaction. J Transl Med. (2011) 9:51. 10.1186/1479-5876-9-51 21545725 PMC3112438

[101] Zhuo W Liao L Xu T Wu W Yang S Tan J. Mesenchymal stem cells ameliorate ischemia-reperfusion-induced renal dysfunction by improving the antioxidant/oxidant balance in the ischemic kidney. Urol Int. (2011) 86:191–6. 10.1159/000319366 20881358

[102] Zhang G Zou X Huang Y Wang F Miao S Liu G. Mesenchymal stromal cell-derived extracellular vesicles protect against acute kidney injury through anti-oxidation by enhancing Nrf2/ARE activation in rats. Kidney Blood Press Res. (2016) 41:119–28. 10.1159/000443413 26894749

[103] Moll G Jitschin R von Bahr L Rasmusson-Duprez I Sundberg B Lönnies L. Mesenchymal stromal cells engage complement and complement receptor bearing innate effector cells to modulate immune responses. PLoS ONE. (2011) 6:e21703. 10.1371/journal.pone.0021703 21747949 PMC3128611

[104] Zilberman-Itskovich S Abu-Hamad R Zarura R Sova M Hachmo Y Stark M. Human mesenchymal stromal cells ameliorate complement induced inflammatory cascade and improve renal functions in a rat model of ischemia-reperfusion induced acute kidney injury. PLoS ONE. (2019) 14:e0222354. 10.1371/journal.pone.0222354 31513644 PMC6741994

[105] Tang M Zhang K Li Y He Q Li G Zheng Q. Mesenchymal stem cells alleviate acute kidney injury by down-regulating C5a/C5aR pathway activation. Int Urol Nephrol. (2018) 50:1545–53. 10.1007/s11255-018-1844-7 29594894

[106] Zhou L Xu L Shen J Song Q Wu R Ge Y. Preischemic administration of nonexpanded adipose stromal vascular fraction attenuates acute renal ischemia/reperfusion injury and fibrosis. Stem Cell Transl Med. (2016) 5:1277–88. 10.5966/sctm.2015-0223 27365485 PMC4996434

[107] Zhou L Song Q Shen J Xu L Xu Z Wu R. Comparison of human adipose stromal vascular fraction and adipose-derived mesenchymal stem cells for the attenuation of acute renal ischemia/reperfusion injury. Sci Rep. (2017) 7:44058. 10.1038/srep44058 28276451 PMC5343423

[108] Zou X Zhang G Cheng Z Yin D Du T Ju G. Microvesicles derived from human Wharton's Jelly mesenchymal stromal cells ameliorate renal ischemia-reperfusion injury in rats by suppressing CX3CL1. Stem Cell Res Ther. (2014) 5:40. 10.1186/scrt428 24646750 PMC4055103

[109] Feng Z Ting J Alfonso Z Strem BM Fraser JK Rutenberg J. Fresh and cryopreserved, uncultured adipose tissue-derived stem and regenerative cells ameliorate ischemia–reperfusion-induced acute kidney injury. Nephrol Dial Transpl. (2010) 25:3874–84. 10.1093/ndt/gfq603 20921297 PMC2989793

[110] Collett JA Traktuev DO Mehrotra P Crone A Merfeld-Clauss S March KL. Human adipose stromal cell therapy improves survival and reduces renal inflammation and capillary rarefaction in acute kidney injury. J Cell Mol Med. (2017) 21:1420–30. 10.1111/jcmm.13071 28455887 PMC5487924

[111] Tsuda H Yamahara K Otani K Okumi M Yazawa K Kaimori J. Transplantation of allogenic fetal membrane-derived mesenchymal stem cells protects against ischemia/reperfusion-induced acute kidney injury. Cell Transpl. (2014) 23:889–99. 10.3727/096368913x665594 23562186

[112] Liu X Cai J Jiao X Yu X Ding X. Therapeutic potential of mesenchymal stem cells in acute kidney injury is affected by administration timing. Acta Bioch Bioph Sin. (2017) 49:338–48. 10.1093/abbs/gmx016 28338909

[113] Jia X Pan J Li X Li N Han Y Feng X. Bone marrow mesenchymal stromal cells ameliorate angiogenesis and renal damage via promoting PI3k-Akt signaling pathway activation *in vivo*. Cytotherapy. (2016) 18:838–45. 10.1016/j.jcyt.2016.03.300 27210720

[114] Tögel F Zhang P Hu Z Westenfelder C. VEGF is a mediator of the renoprotective effects of multipotent marrow stromal cells in acute kidney injury. J Cell Mol Med. (2008) 13:2109–14. 10.1111/j.1582-4934.2008.00641.x 19397783 PMC4940776

[115] Tögel F Cohen A Zhang P Yang Y Hu Z Westenfelder C. Autologous and allogeneic marrow stromal cells are safe and effective for the treatment of acute kidney injury. Stem Cells Dev. (2009) 18:475–85. 10.1089/scd.2008.0092 18564903 PMC3190285

[116] Zou X Gu D Xing X Cheng Z Gong D Zhang G. Human mesenchymal stromal cell-derived extracellular vesicles alleviate renal ischemic reperfusion injury and enhance angiogenesis in rats. Am J Transl Res. (2016) 8:4289–99. 27830012 PMC5095321

[117] Lindoso RS Collino F Bruno S Araujo DS Sant'Anna JF Tetta C. Extracellular vesicles released from mesenchymal stromal cells modulate miRNA in renal tubular cells and inhibit ATP depletion injury. Stem Cells Dev. (2014) 23:1809–19. 10.1089/scd.2013.0618 24669934 PMC4103261

[118] Perico L Morigi M Rota C Breno M Mele C Noris M. Human mesenchymal stromal cells transplanted into mice stimulate renal tubular cells and enhance mitochondrial function. Nat Commun. (2017) 8:983. 10.1038/s41467-017-00937-2 29042548 PMC5754365

[119] Gu D Zou X Ju G Zhang G Bao E Zhu Y. Mesenchymal stromal cells derived extracellular vesicles ameliorate acute renal ischemia reperfusion injury by inhibition of mitochondrial fission through miR-30. Stem Cells Int. (2016) 2016:1–12. 10.1155/2016/2093940 27799943 PMC5069372

[120] Zhang F Wan X Cao Y Sun D Cao C. Klotho gene-modified BMSCs showed elevated antifibrotic effects by inhibiting the Wnt/β-catenin pathway in kidneys after acute injury. Cell Biol Int. (2018) 42:1670–79. 10.1002/cbin.11068 30358003

[121] Damasceno PKF de Santana TA Santos GC Orge ID Silva DN Albuquerque JF. Genetic engineering as a strategy to improve the therapeutic efficacy of mesenchymal stem/stromal cells in regenerative medicine. Front Cell Dev Biol. (2020) 8:737. 10.3389/fcell.2020.00737 32974331 PMC7471932

[122] Liu Y Fang J. Mesenchymal stem cells as therapeutic agents and novel carriers for the delivery of candidate genes in acute kidney injury. Stem Cells Int. (2020) 2020:8875554. 10.1155/2020/8875554 33381189 PMC7748887

[123] Jang MJ You D Park JY Kim K Aum J Lee C. Hypoxic preconditioned mesenchymal stromal cell therapy in a rat model of renal ischemia-reperfusion injury: development of optimal protocol to potentiate therapeutic efficacy. Int J Stem Cells. (2018) 11:157–67. 10.15283/ijsc18073 30497128 PMC6285294

[124] Zhang W Liu L Huo Y Yang Y Wang Y. Hypoxia-pretreated human MSCs attenuate acute kidney injury through enhanced angiogenic and antioxidative capacities. Biomed Res Int. (2014) 2014:1–10. 10.1155/2014/462472 25133162 PMC4123714

[125] Collino F Lopes JA Corrêa S Abdelhay E Takiya CM Wendt CHC. Adipose-derived mesenchymal stromal cells under hypoxia: changes in extracellular vesicles secretion and improvement of renal recovery after ischemic injury. Cell Physiol Biochem. (2019) 52:1463–83. 10.33594/000000102 31099507

[126] Noronha N de C Mizukami A Caliári-Oliveira C Cominal JG Rocha JLM Covas DT. Priming approaches to improve the efficacy of mesenchymal stromal cell-based therapies. Stem Cell Res Ther. (2019) 10:131. 10.1186/s13287-019-1224-y 31046833 PMC6498654

[127] Miceli V Bulati M Iannolo G Zito G Gallo A Conaldi PG. Therapeutic properties of mesenchymal stromal/stem cells: the need of cell priming for cell-free therapies in regenerative medicine. Int J Mol Sci. (2021) 22:763. 10.3390/ijms22020763 33466583 PMC7828743

[128] Kanai R Nakashima A Doi S Kimura T Yoshida K Maeda S. Interferon-γ enhances the therapeutic effect of mesenchymal stem cells on experimental renal fibrosis. Sci Rep. (2021) 11:850. 10.1038/s41598-020-79664-6 33441701 PMC7807061

[129] Gao L Zhong X Jin J Li J Meng X. Potential targeted therapy and diagnosis based on novel insight into growth factors, receptors, and downstream effectors in acute kidney injury and acute kidney injury-chronic kidney disease progression. Signal Transduct Target Ther. (2020) 5:9. 10.1038/s41392-020-0106-1 32296020 PMC7018831

[130] Chawla LS Kimmel PL. Acute kidney injury and chronic kidney disease: an integrated clinical syndrome. Kidney Int. (2012) 82:516–524. 10.1038/ki.2012.208 22673882

[131] Leung KCW Tonelli M James MT. Chronic kidney disease following acute kidney injury—risk and outcomes. Nat Rev Nephrol. (2012) 9:77–85. 10.1038/nrneph.2012.280 23247572

[132] Kiriakidou M Ching CL. Systemic Lupus Erythematosus. Ann Intern Med. (2020) 172:ITC81–ITC96. 10.7326/aitc202006020 32479157

[133] D'Agati VD Kaskel FJ Falk RJ. Focal Segmental Glomerulosclerosis. New Engl J Medicine. (2011) 365:2398–411. 10.1056/nejmra1106556 22187987

[134] Heung M Chawla LS. Acute kidney injury: gateway to chronic kidney disease. Nephron Clin Pract. (2014) 127:30–4. 10.1159/000363675 25343817

[135] Zeisberg M Neilson EG. Mechanisms of tubulointerstitial fibrosis. J Am Soc Nephrol. (2010) 21:1819–34. 10.1681/asn.2010080793 20864689

[136] Gatti S Bruno S Deregibus MC Sordi A Cantaluppi V Tetta C. Microvesicles derived from human adult mesenchymal stem cells protect against ischaemia–reperfusion-induced acute and chronic kidney injury. Nephrol Dial Transpl. (2011) 26:1474–83. 10.1093/ndt/gfr015 21324974

[137] Sun D Bu L Liu C Yin Z Zhou X Li X. Therapeutic effects of human amniotic fluid-derived stem cells on renal interstitial fibrosis in a murine model of unilateral ureteral obstruction. PLoS ONE. (2013) 8:e65042. 10.1371/journal.pone.0065042 23724119 PMC3665750

[138] Donizetti-Oliveira C Semedo P Burgos-Silva M Cenedeze MA Malheiros DMAC Reis MA. Adipose tissue-derived stem cell treatment prevents renal disease progression. Cell Transpl. (2012) 21:1727–41. 10.3727/096368911x623925 22305061

[139] Zhu F Shin OLSCL Pei G Hu Z Yang J Zhu H. Adipose-derived mesenchymal stem cells employed exosomes to attenuate AKI-CKD transition through tubular epithelial cell dependent Sox9 activation. Oncotarget. (2017) 8:70707–26. 10.18632/oncotarget.19979 29050313 PMC5642588

[140] Wang B Yao K Huuskes BM Shen H-H Zhuang J Godson C. Mesenchymal stem cells deliver exogenous MicroRNA-let7c via exosomes to attenuate renal fibrosis. Mol Ther. (2016) 24:1290–301. 10.1038/mt.2016.90 27203438 PMC5088767

[141] Semedo P Correa-Costa M Cenedeze MA Malheiros DMAC dos Reis MA Shimizu MH. Mesenchymal stem cells attenuate renal fibrosis through immune modulation and remodeling properties in a rat remnant kidney model. Stem Cells. (2009) 27:214. 10.1002/stem.214 19750536

[142] van Koppen A Joles JA Bongartz LG van den Brandt J Reichardt HM Goldschmeding R. Healthy bone marrow cells reduce progression of kidney failure better than CKD bone marrow cells in rats with established chronic kidney disease. Cell Transplant. (2012) 21:2299–312. 10.3727/096368912x636795 23231961

[143] Rivera-Valdés JJ García-Bañuelos J Salazar-Montes A García-Benavides L Rosales-Dominguez A Armendáriz-Borunda J. Human adipose derived stem cells regress fibrosis in a chronic renal fibrotic model induced by adenine. PLoS ONE. (2017) 12:e0187907. 10.1371/journal.pone.0187907 29281649 PMC5744925

[144] Jang E Jeong M Kim S Jang K Kang B-K Lee DY. Infusion of human bone marrow-derived mesenchymal stem cells alleviates autoimmune nephritis in a lupus model by suppressing follicular helper T-cell development. Cell Transplant. (2015) 25:1–15. 10.3727/096368915x688173 25975931

[145] Yuan X Qin X Wang D Zhang Z Tang X Gao X. Mesenchymal stem cell therapy induces FLT3L and CD1c+ dendritic cells in systemic lupus erythematosus patients. Nat Commun. (2019) 10:2498. 10.1038/s41467-019-10491-8 31175312 PMC6555800

[146] Zhou T Liao C Li H-Y Lin W Lin S Zhong H. Efficacy of mesenchymal stem cells in animal models of lupus nephritis: a meta-analysis. Stem Cell Res Ther. (2020) 11:48. 10.1186/s13287-019-1538-9 32019582 PMC7001209

[147] Nagaishi K Mizue Y Chikenji T Otani M Nakano M Konari N. Mesenchymal stem cell therapy ameliorates diabetic nephropathy via the paracrine effect of renal trophic factors including exosomes. Sci Rep-uk. (2016) 6:34842. 10.1038/srep34842 27721418 PMC5056395

[148] Fang Y Tian X Bai S Fan J Hou W Tong H. Autologous transplantation of adipose-derived mesenchymal stem cells ameliorates streptozotocin-induced diabetic nephropathy in rats by inhibiting oxidative stress, pro-inflammatory cytokines and the p38 MAPK signaling pathway. Int J Mol Med. (2012) 30:85–92. 10.3892/ijmm.2012.977 22552764

[149] Lv S Liu G Sun A Wang J Cheng J Wang W. Mesenchymal stem cells ameliorate diabetic glomerular fibrosis *in vivo* and *in vitro* by inhibiting TGF-β signalling via secretion of bone morphogenetic protein 7. Diabetes Vasc Dis Res. (2014) 11:251–61. 10.1177/1479164114531300 24845071

[150] Konari N Nagaishi K Kikuchi S Fujimiya M. Mitochondria transfer from mesenchymal stem cells structurally and functionally repairs renal proximal tubular epithelial cells in diabetic nephropathy *in vivo*. Sci Rep. (2019) 9:5184. 10.1038/s41598-019-40163-y 30914727 PMC6435708

[151] Ge W Jiang J Arp J Liu W Garcia B Wang H. Regulatory T-cell generation and kidney allograft tolerance induced by mesenchymal stem cells associated with indoleamine 2,3-dioxygenase expression. Transplantation. (2010) 90:1312–20. 10.1097/tp.0b013e3181fed001 21042238

[152] Bai M Zhang L Fu B Bai J Zhang Y Cai G. IL-17A improves the efficacy of mesenchymal stem cells in ischemic-reperfusion renal injury by increasing Treg percentages by the COX-2/PGE2 pathway. Kidney Int. (2018) 93:814–25. 10.1016/j.kint.2017.08.030 29132705

[153] Casiraghi F Todeschini M Azzollini N Cravedi P Cassis P Solini S. Effect of timing and complement receptor antagonism on intragraft recruitment and pro-tolerogenic effects of mesenchymal stromal cells in murine kidney transplantation. Transplantation. (2019) 103:1121–30. 10.1097/tp.0000000000002611 30801518 PMC6934941

[154] Casiraghi F Azzollini N Todeschini M Cavinato RA Cassis P Solini S. Localization of mesenchymal stromal cells dictates their immune or proinflammatory effects in kidney transplantation. Am J Transplant. (2012) 12:2373–83. 10.1111/j.1600-6143.2012.04115.x 22642544

[155] Sivanathan KN Rojas-Canales DM Hope CM Krishnan R Carroll RP Gronthos S. Interleukin-17A-induced human mesenchymal stem cells are superior modulators of immunological function. Stem Cells. (2015) 33:2850–63. 10.1002/stem.2075 26037953

[156] Franquesa M Herrero E Torras J Ripoll E Flaquer M Gomà M. Mesenchymal stem cell therapy prevents interstitial fibrosis and tubular atrophy in a rat kidney allograft model. Stem Cells Dev. (2012) 21:3125–35. 10.1089/scd.2012.0096 22494435 PMC3495114

[157] Martino MD Zonta S Rampino T Gregorini M Frassoni F Piotti G. Mesenchymal stem cells infusion prevents acute cellular rejection in rat kidney transplantation. Transplant Prev. (2010) 42:1331–5. 10.1016/j.transproceed.2010.03.079 20534294

[158] Lee S-R Lee S-H Moon J-Y Park J-Y Lee D Lim SJ. Repeated administration of bone marrow-derived mesenchymal stem cells improved the protective effects on a remnant kidney model. Renal Failure. (2010) 32:840–8. 10.3109/0886022x.2010.494803 20662698

[159] Cai J Jiao X Zhao S Liang Y Ning Y Shi Y. Transforming growth factor-β1-overexpressing mesenchymal stromal cells induced local tolerance in rat renal ischemia/reperfusion injury. Cytotherapy. (2019) 21:535–45. 10.1016/j.jcyt.2018.12.003 30685215

[160] Cao Z Zhang G Wang F Liu H Liu L Han Y. Protective effects of mesenchymal stem cells with CXCR4 up-regulation in a rat renal transplantation model. PLoS ONE. (2013) 8:e82949. 10.1371/journal.pone.0082949 24386129 PMC3875425

[161] Gregorini M Bosio F Rocca C Corradetti V Valsania T Pattonieri EF. Mesenchymal stromal cells reset the scatter factor system and cytokine network in experimental kidney transplantation. BMC Immunol. (2014) 15:44. 10.1186/s12865-014-0044-1 25277788 PMC4193986

[162] Zonta S Martino MD Bedino G Piotti G Rampino T Gregorini M. Which is the most suitable and effective route of administration for mesenchymal stem cell-based immunomodulation therapy in experimental kidney transplantation: endovenous or arterial? Transplant Prev. (2010) 42:1336–40. 10.1016/j.transproceed.2010.03.081 20534295

[163] Ramirez-Bajo MJ Rovira J Lazo-Rodriguez M Banon-Maneus E Tubita V Moya-Rull D. Impact of mesenchymal stromal cells and their extracellular vesicles in a rat model of kidney rejection. Front Cell Dev Biol. (2020) 8:10. 10.3389/fcell.2020.00010 32064259 PMC7000363

[164] Koch M Lehnhardt A Hu X Brunswig-Spickenheier B Stolk M Bröcker V. Isogeneic MSC application in a rat model of acute renal allograft rejection modulates immune response but does not prolong allograft survival. Transpl Immunol. (2013) 29:43–50. 10.1016/j.trim.2013.08.004 23994720

[165] Mendicino M Bailey AM Wonnacott K Puri RK Bauer SR. MSC-based product characterization for clinical trials: an FDA perspective. Cell Stem Cell. (2014) 14:141–5. 10.1016/j.stem.2014.01.013 24506881

[166] Eggenhofer E Benseler V Kroemer A Popp FC Geissler EK Schlitt HJ. Mesenchymal stem cells are short-lived and do not migrate beyond the lungs after intravenous infusion. Front Immunol. (2012) 3:297. 10.3389/fimmu.2012.00297 23056000 PMC3458305

[167] Fischer UM Harting MT Jimenez F Monzon-Posadas WO Xue H Savitz SI. Pulmonary passage is a major obstacle for intravenous stem cell delivery: the pulmonary first-pass effect. Stem Cells Dev. (2009) 18:683–92. 10.1089/scd.2008.0253 19099374 PMC3190292

[168] Moll G Rasmusson-Duprez I von Bahr L Connolly-Andersen A Elgue G Funke L. Are therapeutic human mesenchymal stromal cells compatible with human blood? Stem Cells. (2012) 30:1565–74. 10.1002/stem.1111 22522999

[169] Gleeson BM Martin K Ali MT Kumar AHS Pillai MG-K Kumar SPG. Bone marrow-derived mesenchymal stem cells have innate procoagulant activity and cause microvascular obstruction following intracoronary delivery: amelioration by antithrombin therapy. Stem Cells Dayt Ohio. (2015) 33:2726–37. 10.1002/stem.2050 25969127

[170] Christy BA Herzig MC Montgomery RK Delavan C Bynum JA Reddoch KM. Procoagulant activity of human mesenchymal stem cells. J Trauma Acute Care. (2017) 83:S164–9. 10.1097/ta.0000000000001485 28628602

[171] Bamoulid J Staeck O Halleck F Khadzhynov D Brakemeier S Dürr M. The need for minimization strategies: current problems of immunosuppression. Transplant Int. (2015) 28:891–900. 10.1111/tri.12553 25752992

[172] Berglund AK Fortier LA Antczak DF Schnabel LV. Immunoprivileged no more: measuring the immunogenicity of allogeneic adult mesenchymal stem cells. Stem Cell Res Ther. (2017) 8:288. 10.1186/s13287-017-0742-8 29273086 PMC5741939

[173] la Torre RS Quiñones-Vico MI Fernández-González A Sánchez-Díaz M Montero-Vílchez T Sierra-Sánchez Á. Alloreactive immune response associated to human mesenchymal stromal cells treatment: a systematic review. J Clin Medicine. (2021) 10:2991. 10.3390/jcm10132991 34279481 PMC8269175

[174] Ankrum JA Ong JF Karp JM. Mesenchymal stem cells: immune evasive, not immune privileged. Nat Biotechnol. (2014) 32:252–60. 10.1038/nbt.2816 24561556 PMC4320647

[175] Lohan P Treacy O Griffin MD Ritter T Ryan AE. Anti-donor immune responses elicited by allogeneic mesenchymal stem cells and their extracellular vesicles: are we still learning? Front Immunol. (2017) 8:1626. 10.3389/fimmu.2017.01626 29225601 PMC5705547

[176] Hoogduijn MJ Lombardo E. Mesenchymal stromal cells anno 2019: dawn of the therapeutic era? Concise Review. Stem Cell Transl Med. (2019) 8:1126–34. 10.1002/sctm.19-0073 31282113 PMC6811696

[177] Chabot D Lewin A Loubaki L Bazin R. Functional impairment of MSC induced by transient warming events: correlation with loss of adhesion and altered cell size. Cytotherapy. (2018) 20:990–1000. 10.1016/j.jcyt.2018.05.010 30093326

[178] Moll G Alm JJ Davies LC von Bahr L Heldring N Stenbeck-Funke L. Do cryopreserved mesenchymal stromal cells display impaired immunomodulatory and therapeutic properties? Stem Cells. (2014) 32:2430–42. 10.1002/stem.1729 24805247 PMC4381870

[179] François M Copland IB Yuan S Romieu-Mourez R Waller EK Galipeau J. Cryopreserved mesenchymal stromal cells display impaired immunosuppressive properties as a result of heat-shock response and impaired interferon-γ licensing. Cytotherapy. (2011) 14:147–52. 10.3109/14653249.2011.623691 22029655 PMC3279133

[180] Chinnadurai R Copland IB Garcia MA Petersen CT Lewis CN Waller EK. Cryopreserved mesenchymal stromal cells are susceptible to t-cell mediated apoptosis which is partly rescued by IFNγ licensing: thawed MSCs are susceptible to T cell mediated lysis. Stem Cells. (2016) 34:2429–42. 10.1002/stem.2415 27299362 PMC5016228

[181] Hoogduijn MJ de Witte SFH Luk F Vroonhoven MCGN van den H Ignatowicz L Catar R. Effects of freeze-thawing and intravenous infusion on mesenchymal stromal cell gene expression. Stem Cells Dev. (2016) 25:586–97. 10.1089/scd.2015.0329 26914168

[182] Gramlich OW Burand AJ Brown AJ Deutsch RJ Kuehn MH Ankrum JA. Cryopreserved mesenchymal stromal cells maintain potency in a retinal ischemia/reperfusion injury model: toward an off-the-shelf therapy. Sci Rep. (2016) 6:26463. 10.1038/srep26463 27212469 PMC4876464

[183] Luetzkendorf J Nerger K Hering J Moegel A Hoffmann K Hoefers C. Cryopreservation does not alter main characteristics of Good Manufacturing Process-grade human multipotent mesenchymal stromal cells including immunomodulating potential and lack of malignant transformation. Cytotherapy. (2015) 17:186–98. 10.1016/j.jcyt.2014.10.018 25593077

[184] Galipeau J Krampera M Leblanc K Nolta JA Phinney DG Shi Y. Mesenchymal stromal cell variables influencing clinical potency: the impact of viability, fitness, route of administration and host predisposition. Cytotherapy. (2021) 23:368–72. 10.1016/j.jcyt.2020.11.007 33714704 PMC11708105

[185] Yuan X Logan TM Ma T. Metabolism in human mesenchymal stromal cells: a missing link between hMSC biomanufacturing and therapy? Front Immunol. (2019) 10:977. 10.3389/fimmu.2019.00977 31139179 PMC6518338

[186] Oja S Kaartinen T Ahti M Korhonen M Laitinen A Nystedt J. The Utilization of freezing steps in Mesenchymal Stromal Cell (MSC) manufacturing: potential impact on quality and cell functionality attributes. Front Immunol. (2019) 10:1627. 10.3389/fimmu.2019.01627 31379832 PMC6646664

[187] Galleu A Riffo-Vasquez Y Trento C Lomas C Dolcetti L Cheung TS. Apoptosis in mesenchymal stromal cells induces in vivo recipient-mediated immunomodulation. Sci Transl Med. (2017) 9:eaam7828. 10.1126/scitranslmed.aam7828 29141887

[188] Witte SFH Luk F Parraga JMS Gargesha M Merino A Korevaar SS. Immunomodulation by Therapeutic Mesenchymal Stromal Cells (MSC) is triggered through phagocytosis of MSC by monocytic cells. Stem Cells. (2018) 36:602–15. 10.1002/stem.2779 29341339

[189] Weiss DJ English K Krasnodembskaya A Isaza-Correa JM Hawthorne IJ Mahon BP. The necrobiology of mesenchymal stromal cells affects therapeutic efficacy. Front Immunol. (2019) 10:1228. 10.3389/fimmu.2019.01228 31214185 PMC6557974

[190] Moll G Geißler S Catar R Ignatowicz L Hoogduijn MJ Strunk D Bieback K Ringdén O. Cryopreserved or Fresh Mesenchymal Stromal Cells: Only a Matter of Taste or Key to Unleash the Full Clinical Potential of MSC Therapy? Adv Exp Med Biol. (2016) 951:77–98. 10.1007/978-3-319-45457-3_7 27837556

[191] Third Party Mesenchymal Stromal Cell Infusion in Kidney Transplant Recipient: 6-Month Safety Interim Analysis - ATC Abstracts. Available online at: https://atcmeetingabstracts.com/abstract/third-party-mesenchymal-stromal-cell-infusion-in-kidney-transplant-recipient-6-month-safety-interim-analysis/ (accessed May 7, 2021).

[192] Assfalg V Hüser N van Meel M Haller B Rahmel A de Boer J. High-urgency kidney transplantation in the Eurotransplant Kidney Allocation System: success or waste of organs? The Eurotransplant 15-year all-centre survey. Nephrol Dial Transpl. (2016) 31:1515–22. 10.1093/ndt/gfv446 26908765

[193] Summers DM Johnson RJ Allen J Fuggle SV Collett D Watson CJ. Analysis of factors that affect outcome after transplantation of kidneys donated after cardiac death in the UK: a cohort study. Lancet. (2010) 376:1303–11. 10.1016/s0140-6736(10)60827-6 20727576

[194] Snoeijs MGJ Winkens B Heemskerk MBA Hoitsma AJ Christiaans MHL Buurman WA Heurn LWE. Kidney transplantation from donors after cardiac death; a 25-year experience. Transplantation. (2010) 90:1106–12. 10.1097/tp.0b013e3181f83b0b 20861804

[195] Casiraghi F Remuzzi G. Mesenchymal stromal cells in kidney transplantation. Curr Opin Nephrol Hyg. (2019) 28:40–6. 10.1097/mnh.0000000000000461 30300159

[196] Peerapornratana S Manrique-Caballero CL Gómez H Kellum JA. Acute kidney injury from sepsis: current concepts, epidemiology, pathophysiology, prevention and treatment. Kidney Int. (2019) 96:1083–99. 10.1016/j.kint.2019.05.026 31443997 PMC6920048

[197] Liu Y Cui J Wang H Hezam K Zhao X Huang H. Enhanced therapeutic effects of MSC-derived extracellular vesicles with an injectable collagen matrix for experimental acute kidney injury treatment. Stem Cell Res Ther. (2020) 11:161. 10.1186/s13287-020-01668-w 32321594 PMC7178991

[198] Miller BLK Garg P Bronstein B LaPointe E Lin H Charytan DM. Extracorporeal stromal cell therapy for subjects with dialysis-dependent acute kidney injury. Kidney Int Rep. (2018) 3:1119–27. 10.1016/j.ekir.2018.05.009 30197978 PMC6127415

[199] Ko K-W Park S-Y Lee EH Yoo Y-I Kim D-S Kim JY. Integrated bioactive scaffold with polydeoxyribonucleotide and stem-cell-derived extracellular vesicles for kidney regeneration. Acs Nano. (2021) 15:7575–85. 10.1021/acsnano.1c01098 33724774

[200] Malhotra R Siew ED. Biomarkers for the early detection and prognosis of acute kidney injury. Clin J Am Soc Nephro. (2017) 12:149–73. 10.2215/cjn.01300216 27827308 PMC5220647

[201] Textor SC Lerman LO. Paradigm shifts in atherosclerotic renovascular disease: where are we now? J Am Soc Nephrol Jasn. (2015) 26:2074–80. 10.1681/asn.2014121274 25868641 PMC4552124

[202] Chade AR Rodriguez-Porcel M Grande JP Krier JD Lerman A Romero JC. Distinct renal injury in early atherosclerosis and renovascular disease. Circulation. (2002) 106:1165–71. 10.1161/01.cir.0000027105.02327.48 12196346

[203] Nassar W El-Ansary M Sabry D Mostafa MA Fayad T Kotb E. Umbilical cord mesenchymal stem cells derived extracellular vesicles can safely ameliorate the progression of chronic kidney diseases. Biomater Res. (2016) 20:21. 10.1186/s40824-016-0068-0 27499886 PMC4974791

[204] Rani S Ryan AE Griffin MD Ritter T. Mesenchymal stem cell-derived extracellular vesicles: toward cell-free therapeutic applications. Mol Ther. (2015) 23:812–23. 10.1038/mt.2015.44 25868399 PMC4427881

[205] Rhijn MR Reinders MEJ de Klein A Douben H Korevaar SS Mensah FKF. Mesenchymal stem cells derived from adipose tissue are not affected by renal disease. Kidney Int. (2012) 82:748–58. 10.1038/ki.2012.187 22695328

[206] Reinders MEJ Rhijn MR Khairoun M Lievers E de Vries DK Schaapherder AFM. Bone marrow-derived mesenchymal stromal cells from patients with end-stage renal disease are suitable for autologous therapy. Cytotherapy. (2013) 15:663–72. 10.1016/j.jcyt.2013.01.010 23419679

[207] van Rhijn-Brouwer FCC van Balkom BWM Papazova DA Hazenbrink DHM Meijer AJ Brete I. Paracrine proangiogenic function of human bone marrow-derived mesenchymal stem cells is not affected by chronic kidney disease. Stem Cells Int. (2019) 2019:1–12. 10.1155/2019/1232810 31933648 PMC6942892

[208] Tuttle KR Bakris GL Bilous RW Chiang JL de Boer IH Goldstein-Fuchs J. Diabetic kidney disease: a report from an ADA consensus conference. Am J Kidney Dis. (2014) 64:510–33. 10.1053/j.ajkd.2014.08.001 25257325

[209] Skyler JS Fonseca VA Segal KR Rosenstock J Investigators M-D. Allogeneic mesenchymal precursor cells in type 2 diabetes: a randomized, placebo-controlled, dose-escalation safety and tolerability pilot study. Diabetes Care. (2015) 38:1742–9. 10.2337/dc14-2830 26153271 PMC4542273

[210] Mahmoud M Abu-Shahba N Azmy O El-Badri N. Impact of diabetes mellitus on human mesenchymal stromal cell biology and functionality: implications for autologous transplantation. Stem Cell Rev Rep. (2019) 15:194–217. 10.1007/s12015-018-9869-y 30680660

[211] Cassidy FC Shortiss C Murphy CG Kearns SR Curtin W Buitléir CD. Impact of type 2 diabetes mellitus on human bone marrow stromal cell number and phenotypic characteristics. Int J Mol Sci. (2020) 21:2476. 10.3390/ijms21072476 32252490 PMC7177361

[212] Yu F Haas M Glassock R Zhao M-H. Redefining lupus nephritis: clinical implications of pathophysiologic subtypes. Nat Rev Nephrol. (2017) 13:483–95. 10.1038/nrneph.2017.85 28669995

[213] Cheng R-J Xiong A-J Li Y-H Pan S-Y Zhang Q-P Zhao Y. Mesenchymal stem cells: allogeneic MSC may be immunosuppressive but autologous MSC are dysfunctional in lupus patients. Front Cell Dev Biol. (2019) 7:285. 10.3389/fcell.2019.00285 31799252 PMC6874144

[214] Wang D Wang S Huang S Zhang Z Yuan X Feng X. Serum IFN-γ predicts the therapeutic effect of mesenchymal stem cells transplantation in systemic lupus erythematosus patients. Stem Cell Transl Med. (2017) 6:1777–85. 10.1002/sctm.17-0002 28755405 PMC5689761

[215] Wang D Akiyama K Zhang H Yamaza T Li X Feng X. Double allogenic mesenchymal stem cells transplantations could not enhance therapeutic effect compared with single transplantation in systemic lupus erythematosus. Clin Dev Immunol. (2012) 2012:273291. 10.1155/2012/273291 22829849 PMC3399403

[216] Wegmeyer H Bröske A-M Leddin M Kuentzer K Nisslbeck AK Hupfeld J. Mesenchymal stromal cell characteristics vary depending on their origin. Stem Cells Dev. (2013) 22:2606–18. 10.1089/scd.2013.0016 23676112 PMC3780294

[217] Kern S Eichler H Stoeve J Klüter H Bieback K. Comparative analysis of mesenchymal stem cells from bone marrow, umbilical cord blood, or adipose tissue. Stem Cells. (2006) 24:1294–301. 10.1634/stemcells.2005-0342 16410387

[218] Moll G Ignatowicz L Catar R Luecht C Sadeghi B Hamad O. Different procoagulant activity of therapeutic mesenchymal stromal cells derived from bone marrow and placental decidua. Stem Cells Dev. (2015) 24:2269–79. 10.1089/scd.2015.0120 26192403

[219] Winkel A Jaimes Y Melzer C Dillschneider P Hartwig H Stiesch M. Cell culture media notably influence properties of human mesenchymal stroma/stem-like cells from different tissues. Cytotherapy. (2020) 22:653–68. 10.1016/j.jcyt.2020.07.005 32855067

[220] Khan RS Newsome PN. A comparison of phenotypic and functional properties of mesenchymal stromal cells and multipotent adult progenitor cells. Front Immunol. (2019) 10:1952. 10.3389/fimmu.2019.01952 31555259 PMC6724467

[221] Stenderup K Justesen J Clausen C Kassem M. Aging is associated with decreased maximal life span and accelerated senescence of bone marrow stromal cells. Bone. (2003) 33:919–26. 10.1016/j.bone.2003.07.005 14678851

[222] Greenberg SB Grove GL Cristofalo VJ. Cell size in aging monolayer cultures. Vitro. (1977) 13:297–300. 10.1007/bf02616174 326658

[223] Kim M Bae YK Um S Kwon JH Kim G-H Choi SJ. A small-sized population of human umbilical cord blood-derived mesenchymal stem cells shows high stemness properties and therapeutic benefit. Stem Cells Int. (2020) 2020:1–17. 10.1155/2020/5924983 32399043 PMC7204153

[224] Bruder SP Jaiswal N Haynesworth SE. Growth kinetics, self-renewal, and the osteogenic potential of purified human mesenchymal stem cells during extensive subcultivation and following cryopreservation. J Cell Biochem. (1997) 64:278–94. 10.1002/(sici)1097-4644(199702)64:2<278::aid-jcb11>3.0.co;2-f 9027588

[225] Wagner W Ho AD Zenke M. Different facets of aging in human mesenchymal stem cells. Tissue Eng Part B Rev. (2010) 16:445–53. 10.1089/ten.teb.2009.0825 20196648

[226] Fernandez-Rebollo E Franzen J Goetzke R Hollmann J Ostrowska A Oliverio M. Senescence-associated metabolomic phenotype in primary and iPSC-derived mesenchymal stromal cells. Stem Cell Rep. (2020) 14:201–9. 10.1016/j.stemcr.2019.12.012 31983656 PMC7013233

[227] Heathman TRJ Rafiq QA Chan AKC Coopman K Nienow AW Kara B. Characterization of human mesenchymal stem cells from multiple donors and the implications for large scale bioprocess development. Biochem Eng J. (2016) 108:14–23. 10.1016/j.bej.2015.06.018

[228] Bara JJ Richards RG Alini M Stoddart MJ. Concise review: Bone marrow-derived mesenchymal stem cells change phenotype following in vitro culture: implications for basic research and the clinic. Stem Cells Dayt Ohio. (2014) 32:1713–23. 10.1002/stem.1649 24449458

[229] Redaelli S Bentivegna A Foudah D Miloso M Redondo J Riva G. From cytogenomic to epigenomic profiles: monitoring the biologic behavior of in vitro cultured human bone marrow mesenchymal stem cells. Stem Cell Res Ther. (2012) 3:47. 10.1186/scrt138 23168092 PMC3580477

[230] Petrenko Y Vackova I Kekulova K Chudickova M Koci Z Turnovcova K. A comparative analysis of multipotent mesenchymal stromal cells derived from different sources, with a focus on neuroregenerative potential. Sci Rep. (2020) 10:4290. 10.1038/s41598-020-61167-z 32152403 PMC7062771

[231] Hass R Kasper C Böhm S Jacobs R. Different populations and sources of human mesenchymal stem cells (MSC): a comparison of adult and neonatal tissue-derived MSC. Cell Commun Signal. (2011) 9:12. 10.1186/1478-811x-9-12 21569606 PMC3117820

[232] Dmitrieva RI Minullina IR Bilibina AA Tarasova OV Anisimov SV Zaritskey AY. Bone marrow- and subcutaneous adipose tissue-derived mesenchymal stem cells: differences and similarities. Cell Cycle Georget Tex. (2012) 11:377–83. 10.4161/cc.11.2.18858 22189711

[233] Burrow KL Hoyland JA Richardson SM. Human adipose-derived stem cells exhibit enhanced proliferative capacity and retain multipotency longer than donor-matched bone marrow mesenchymal stem cells during expansion *in vitro*. Stem Cells Int. (2017) 2017:1–15. 10.1155/2017/2541275 28553357 PMC5434475

[234] Mohamed-Ahmed S Fristad I Lie SA Suliman S Mustafa K Vindenes H. Adipose-derived and bone marrow mesenchymal stem cells: a donor-matched comparison. Stem Cell Res Ther. (2018) 9:168. 10.1186/s13287-018-0914-1 29921311 PMC6008936

[235] Mohamed SA Howard L McInerney V Hayat A Krawczyk J Naughton S. Autologous bone marrow mesenchymal stromal cell therapy for “no-option” critical limb ischemia is limited by karyotype abnormalities. Cytotherapy. (2020) 22:313–21. 10.1016/j.jcyt.2020.02.007 32273232

[236] Bieback K Kern S Kocaömer A Ferlik K Bugert P. Comparing mesenchymal stromal cells from different human tissues: bone marrow, adipose tissue and umbilical cord blood. Bio-med Mater Eng. (2008) 18:S71–6. 18334717

[237] Baksh D Yao R Tuan RS. Comparison of proliferative and multilineage differentiation potential of human mesenchymal stem cells derived from umbilical cord and bone marrow. Stem Cells. (2007) 25:1384–92. 10.1634/stemcells.2006-0709 17332507

[238] Barlow S Brooke G Chatterjee K Price G Pelekanos R Rossetti T. Comparison of human placenta- and bone marrow–derived multipotent mesenchymal stem cells. Stem Cells Dev. (2008) 17:1095–108. 10.1089/scd.2007.0154 19006451

[239] Choudhery MS Badowski M Muise A Harris DT. Comparison of human mesenchymal stem cells derived from adipose and cord tissue. Cytotherapy. (2013) 15:330–43. 10.1016/j.jcyt.2012.11.010 23318344

[240] Amable PR Teixeira MVT Carias RBV Granjeiro JM Borojevic R. Protein synthesis and secretion in human mesenchymal cells derived from bone marrow, adipose tissue and Wharton's jelly. Stem Cell Res Ther. (2014) 5:53. 10.1186/scrt442 24739658 PMC4055160

[241] Heo JS Choi Y Kim H-S Kim HO. Comparison of molecular profiles of human mesenchymal stem cells derived from bone marrow, umbilical cord blood, placenta and adipose tissue. Int J Mol Med. (2016) 37:115–25. 10.3892/ijmm.2015.2413 26719857 PMC4687432

[242] de Witte SFH Lambert EE Merino A Strini T Douben HJCW O'Flynn L. Aging of bone marrow- and umbilical cord-derived mesenchymal stromal cells during expansion. Cytotherapy. (2017) 19:798–807. 10.1016/j.jcyt.2017.03.071 28462821

[243] Li X Bai J Ji X Li R Xuan Y Wang Y. Comprehensive characterization of four different populations of human mesenchymal stem cells as regards their immune properties, proliferation and differentiation. Int J Mol Med. (2014) 34:695–704. 10.3892/ijmm.2014.1821 24970492 PMC4121354

[244] Jin H Bae Y Kim M Kwon S-J Jeon H Choi S. Comparative analysis of human mesenchymal stem cells from bone marrow, adipose tissue, and umbilical cord blood as sources of cell therapy. Int J Mol Sci. (2013) 14:17986–8001. 10.3390/ijms140917986 24005862 PMC3794764

[245] Batsali AK Pontikoglou C Koutroulakis D Pavlaki KI Damianaki A Mavroudi I. Differential expression of cell cycle and WNT pathway-related genes accounts for differences in the growth and differentiation potential of Wharton's jelly and bone marrow-derived mesenchymal stem cells. Stem Cell Res Ther. (2017) 8:102. 10.1186/s13287-017-0555-9 28446235 PMC5406919

[246] Kehl D Generali M Mallone A Heller M Uldry A-C Cheng P. Proteomic analysis of human mesenchymal stromal cell secretomes: a systematic comparison of the angiogenic potential. Npj Regen Medicine. (2019) 4:8. 10.1038/s41536-019-0070-y 31016031 PMC6467904

[247] Pietilä M Palomäki S Lehtonen S Ritamo I Valmu L Nystedt J. Mitochondrial function and energy metabolism in umbilical cord blood- and bone marrow-derived mesenchymal stem cells. Stem Cells Dev. (2011) 21:575–88. 10.1089/scd.2011.0023 21615273 PMC3280604

[248] Facchin F Bianconi E Romano M Impellizzeri A Alviano F Maioli M. Comparison of oxidative stress effects on senescence patterning of human adult and perinatal tissue-derived stem cells in short and long-term cultures. Int J Med Sci. (2018) 15:1486–501. 10.7150/ijms.27181 30443170 PMC6216057

[249] Zhou W Lin J Zhao K Jin K He Q Hu Y. Single-cell profiles and clinically useful properties of human mesenchymal stem cells of adipose and bone marrow origin. Am J Sports Med. (2019) 47:1722–33. 10.1177/0363546519848678 31100005

[250] Burnham AJ Foppiani EM Horwitz EM. Key metabolic pathways in MSC-mediated immunomodulation: implications for the prophylaxis and treatment of graft versus host disease. Front Immunol. (2020) 11:609277. 10.3389/fimmu.2020.609277 33365034 PMC7750397

[251] Li C Wu X Tong J Yang X Zhao J Zheng Q. Comparative analysis of human mesenchymal stem cells from bone marrow and adipose tissue under xeno-free conditions for cell therapy. Stem Cell Res Ther. (2015) 6:55. 10.1186/s13287-015-0066-5 25884704 PMC4453294

[252] Menard C Pacelli L Bassi G Dulong J Bifari F Bezier I. Clinical-grade mesenchymal stromal cells produced under various good manufacturing practice processes differ in their immunomodulatory properties: standardization of immune quality controls. Stem Cells Dev. (2013) 22:1789–801. 10.1089/scd.2012.0594 23339531 PMC3668498

[253] Dominici M Blanc KL Mueller I Slaper-Cortenbach I Marini FC Krause DS. Minimal criteria for defining multipotent mesenchymal stromal cells. The International Society for Cellular Therapy position statement. Cytotherapy. (2006) 8:315–7. 10.1080/14653240600855905 16923606

[254] Reinisch A Etchart N Thomas D Hofmann NA Fruehwirth M Sinha S. Epigenetic and *in vivo* comparison of diverse MSC sources reveals an endochondral signature for human hematopoietic niche formation. Blood. (2015) 125:249–60. 10.1182/blood-2014-04-572255 25406351 PMC4287636

[255] Xu L Liu Y Sun Y Wang B Xiong Y Lin W. Tissue source determines the differentiation potentials of mesenchymal stem cells: a comparative study of human mesenchymal stem cells from bone marrow and adipose tissue. Stem Cell Res Ther. (2017) 8:275. 10.1186/s13287-017-0716-x 29208029 PMC5718061

[256] Wu M Zhang R Zou Q Chen Y Zhou M Li X. Comparison of the biological characteristics of mesenchymal stem cells derived from the human placenta and umbilical cord. Sci Rep. (2018) 8:5014. 10.1038/s41598-018-23396-1 29568084 PMC5864926

[257] Zhang X Hirai M Cantero S Ciubotariu R Dobrila L Hirsh A. Isolation and characterization of mesenchymal stem cells from human umbilical cord blood: Reevaluation of critical factors for successful isolation and high ability to proliferate and differentiate to chondrocytes as compared to mesenchymal stem cells from bone marrow and adipose tissue. J Cell Biochem. (2011) 112:1206–18. 10.1002/jcb.23042 21312238

[258] Karagianni M Brinkmann I Kinzebach S Grassl M Weiss C Bugert P. A comparative analysis of the adipogenic potential in human mesenchymal stromal cells from cord blood and other sources. Cytotherapy. (2013) 15:76–88.e2. 10.1016/j.jcyt.2012.11.001 23260088

[259] Chang Y Shih DT Tseng C Hsieh T Lee D Hwang S. Disparate mesenchyme-lineage tendencies in mesenchymal stem cells from human bone marrow and umbilical cord blood. Stem Cells. (2006) 24:679–85. 10.1634/stemcells.2004-0308 16179428

[260] Brennan MÁ Barilani M Rusconi F de Lima J Vidal L Lavazza C. Chondrogenic and BMP-4 primings confer osteogenesis potential to human cord blood mesenchymal stromal cells delivered with biphasic calcium phosphate ceramics. Sci Rep. (2021) 11:6751. 10.1038/s41598-021-86147-9 33762629 PMC7991626

[261] Vizoso FJ Eiro N Cid S Schneider J Perez-Fernandez R. Mesenchymal stem cell secretome: toward cell-free therapeutic strategies in regenerative medicine. Int J Mol Sci. (2017) 18:1852. 10.3390/ijms18091852 28841158 PMC5618501

[262] Zha K Li X Yang Z Tian G Sun Z Sui X. Heterogeneity of mesenchymal stem cells in cartilage regeneration: from characterization to application. Npj Regen Med. (2021) 6:14. 10.1038/s41536-021-00122-6 33741999 PMC7979687

[263] Ham O Lee CY Kim R Lee J Oh S Lee MY. Therapeutic potential of differentiated mesenchymal stem cells for treatment of osteoarthritis. Int J Mol Sci. (2015) 16:14961–78. 10.3390/ijms160714961 26147426 PMC4519882

[264] Donders R Bogie JFJ Ravanidis S Gervois P Vanheusden M Marée R. Human Wharton's jelly-derived stem cells display a distinct immunomodulatory and proregenerative transcriptional signature compared to bone marrow-derived stem cells. Stem Cells Dev. (2018) 27:65–84. 10.1089/scd.2017.0029 29267140

[265] Bianco P Cao X Frenette PS Mao JJ Robey PG Simmons PJ. The meaning, the sense and the significance: translating the science of mesenchymal stem cells into medicine. Nat Med. (2013) 19:35–42. 10.1038/nm.3028 23296015 PMC3998103

[266] Wright A Arthaud-Day ML Weiss ML. Therapeutic use of mesenchymal stromal cells: the need for inclusive characterization guidelines to accommodate all tissue sources and species. Front Cell Dev Biol. (2021) 9:632717. 10.3389/fcell.2021.632717 33665190 PMC7921162

[267] Lee NE Kim SJ Yang S-J Joo S-Y Park H Lee KW. Comparative characterization of mesenchymal stromal cells from multiple abdominal adipose tissues and enrichment of angiogenic ability via CD146 molecule. Cytotherapy. (2017) 19:170–80. 10.1016/j.jcyt.2016.11.002 28024875

[268] Witkowski M Landmesser U Rauch U. Tissue factor as a link between inflammation and coagulation. Trends Cardiovas Med. (2016) 26:297–303. 10.1016/j.tcm.2015.12.001 26877187

[269] George MJ Prabhakara K Toledano-Furman NE Wang Y Gill BS Wade CE. Clinical cellular therapeutics accelerate clot formation. Stem Cell Transl Med. (2018) 7:731–9. 10.1002/sctm.18-0015 30070065 PMC6186273

[270] Shiratsuki S Terai S Murata Y Takami T Yamamoto N Fujisawa K. Enhanced survival of mice infused with bone marrow-derived as compared with adipose-derived mesenchymal stem cells. Hepatol Res. (2015) 45:1353–9. 10.1111/hepr.12507 25692387

[271] Busser H Najar M Raicevic G Pieters K Pombo RV Philippart P. Isolation and characterization of human mesenchymal stromal cell subpopulations: comparison of bone marrow and adipose tissue. Stem Cells Dev. (2015) 24:2142–57. 10.1089/scd.2015.0172 26086188

[272] Holley RJ Tai G Williamson AJK Taylor S Cain SA Richardson SM. Comparative quantification of the surfaceome of human multipotent mesenchymal progenitor cells. Stem Cell Rep. (2015) 4:473–88. 10.1016/j.stemcr.2015.01.007 25684225 PMC4375938

[273] Álvarez-Viejo M Menéndez-Menéndez Y Otero-Hernández J. CD271 as a marker to identify mesenchymal stem cells from diverse sources before culture. World J Stem Cells. (2015) 7:470. 10.4252/wjsc.v7.i2.470 25815130 PMC4369502

[274] Murphy MB Moncivais K Caplan AI. Mesenchymal stem cells: environmentally responsive therapeutics for regenerative medicine. Exp Mol Medicine. (2013) 45:e54–e54. 10.1038/emm.2013.94 24232253 PMC3849579

[275] Burja B Barlič A Erman A Mrak-Poljšak K Tomšič M Sodin-Semrl S. Human mesenchymal stromal cells from different tissues exhibit unique responses to different inflammatory stimuli. Curr Res Transl Med. (2020) 68:217–24. 10.1016/j.retram.2020.05.006 32843323

[276] Rezaie J Mehranjani MS Rahbarghazi R Shariatzadeh MA. Angiogenic and restorative abilities of human mesenchymal stem cells were reduced following treatment with serum from diabetes mellitus type 2 patients. J Cell Biochem. (2018) 119:524–35. 10.1002/jcb.26211 28608561

[277] Tsuji K Kitamura S Wada J. Secretomes from mesenchymal stem cells against acute kidney injury: possible heterogeneity. Stem Cells Int. (2018) 2018:1–14. 10.1155/2018/8693137 30651737 PMC6311717

[278] Liu Y Holmes C. Tissue regeneration capacity of extracellular vesicles isolated from bone marrow-derived and adipose-derived mesenchymal stromal/stem cells. Front Cell Dev Biol. (2021) 9:648098. 10.3389/fcell.2021.648098 33718390 PMC7952527

[279] Shin S Lee J Kwon Y Park K-S Jeong J-H Choi S-J. Comparative proteomic analysis of the mesenchymal stem cells secretome from adipose, bone marrow, placenta and Wharton's Jelly. Int J Mol Sci. (2021) 22:845. 10.3390/ijms22020845 33467726 PMC7829982

[280] González PL Carvajal C Cuenca J Alcayaga-Miranda F Figueroa FE Bartolucci J. Chorion mesenchymal stem cells show superior differentiation, immunosuppressive, and angiogenic potentials in comparison with haploidentical maternal placental cells. Stem Cell Transl Med. (2015) 4:1109–21. 10.5966/sctm.2015-0022 26273064 PMC4572900

[281] Lu H Wang F Mei H Wang S Cheng L. Human adipose mesenchymal stem cells show more efficient angiogenesis promotion on endothelial colony-forming cells than umbilical cord and endometrium. Stem Cells Int. (2018) 2018:7537589. 10.1155/2018/7537589 30651736 PMC6311802

[282] Hsiao ST-F Asgari A Lokmic Z Sinclair R Dusting GJ Lim SY. Comparative analysis of paracrine factor expression in human adult mesenchymal stem cells derived from bone marrow, adipose, and dermal tissue. Stem Cells Dev. (2012) 21:2189–203. 10.1089/scd.2011.0674 22188562 PMC3411362

[283] Du WJ Chi Y Yang ZX Li ZJ Cui JJ Song BQ. Heterogeneity of proangiogenic features in mesenchymal stem cells derived from bone marrow, adipose tissue, umbilical cord, and placenta. Stem Cell Res Ther. (2016) 7:163. 10.1186/s13287-016-0418-9 27832825 PMC5103372

[284] Liu J Hao H Xia L Ti D Huang H Dong L. Hypoxia pretreatment of bone marrow mesenchymal stem cells facilitates angiogenesis by improving the function of endothelial cells in diabetic rats with lower ischemia. PLoS ONE. (2015) 10:e0126715. 10.1371/journal.pone.0126715 25996677 PMC4440823

[285] Liu J Hao H Huang H Tong C Ti D Dong L. Hypoxia regulates the therapeutic potential of mesenchymal stem cells through enhanced autophagy. Int J Low Extremity Wounds. (2015) 14:63–72. 10.1177/1534734615573660 25759412

[286] Du W Li X Chi Y Ma F Li Z Yang S. VCAM-1+ placenta chorionic villi-derived mesenchymal stem cells display potent pro-angiogenic activity. Stem Cell Res Ther. (2016) 7:49. 10.1186/s13287-016-0297-0 27044487 PMC4820943

[287] Kachgal S Putnam AJ. Mesenchymal stem cells from adipose and bone marrow promote angiogenesis via distinct cytokine and protease expression mechanisms. Angiogenesis. (2010) 14:47–59. 10.1007/s10456-010-9194-9 21104120 PMC3369878

[288] van Niel G D'Angelo G Raposo G. Shedding light on the cell biology of extracellular vesicles. Nat Rev Mol Cell Bio. (2018) 19:213–28. 10.1038/nrm.2017.125 29339798

[289] Mendt M Rezvani K Shpall E. Mesenchymal stem cell-derived exosomes for clinical use. Bone Marrow Transpl. (2019) 54:789–92. 10.1038/s41409-019-0616-z 31431712

[290] Keshtkar S Azarpira N Ghahremani MH. Mesenchymal stem cell-derived extracellular vesicles: novel frontiers in regenerative medicine. Stem Cell Res Ther. (2018) 9:63. 10.1186/s13287-018-0791-7 29523213 PMC5845209

[291] Börger V Bremer M Ferrer-Tur R Gockeln L Stambouli O Becic A. Mesenchymal stem/stromal cell-derived extracellular vesicles and their potential as novel immunomodulatory therapeutic agents. Int J Mol Sci. (2017) 18:1450. 10.3390/ijms18071450 28684664 PMC5535941

[292] Wang K Jiang Z Webster KA Chen J Hu H Zhou Y. Enhanced cardioprotection by human endometrium mesenchymal stem cells driven by exosomal MicroRNA-21. Stem Cell Transl Med. (2017) 6:209–22. 10.5966/sctm.2015-0386 28170197 PMC5442741

[293] Pomatto M Gai C Negro F Cedrino M Grange C Ceccotti E. Differential therapeutic effect of extracellular vesicles derived by bone marrow and adipose mesenchymal stem cells on wound healing of diabetic ulcers and correlation to their cargoes. Int J Mol Sci. (2021) 22:3851. 10.3390/ijms22083851 33917759 PMC8068154

[294] Liang X Zhang L Wang S Han Q Zhao RC. Exosomes secreted by mesenchymal stem cells promote endothelial cell angiogenesis by transferring miR-125a. J Cell Sci. (2016) 129:2182–9. 10.1242/jcs.170373 27252357

[295] Xue C Shen Y Li X Li B Zhao S Gu J. Exosomes derived from hypoxia-treated human adipose mesenchymal stem cells enhance angiogenesis through the PKA signaling pathway. Stem Cells Dev. (2018) 27:456–65. 10.1089/scd.2017.0296 29415626

[296] Tracy SA Ahmed A Tigges JC Ericsson M Pal AK Zurakowski D. A comparison of clinically relevant sources of mesenchymal stem cell-derived exosomes: bone marrow and amniotic fluid. J Pediatr Surg. (2018) 54:86–90. 10.1016/j.jpedsurg.2018.10.020 30361074

[297] Anderson JD Johansson HJ Graham CS Vesterlund M Pham MT Bramlett CS. Comprehensive proteomic analysis of mesenchymal stem cell exosomes reveals modulation of angiogenesis via nuclear factor-KappaB signaling. Stem Cells. (2016) 34:601–13. 10.1002/stem.2298 26782178 PMC5785927

[298] Teng X Chen L Chen W Yang J Yang Z Shen Z. Mesenchymal stem cell-derived exosomes improve the microenvironment of infarcted myocardium contributing to angiogenesis and anti-inflammation. Cell Physiol Biochem. (2015) 37:2415–24. 10.1159/000438594 26646808

[299] Shabbir A Cox A Rodriguez-Menocal L Salgado M Badiavas EV. Mesenchymal stem cell exosomes induce proliferation and migration of normal and chronic wound fibroblasts, and enhance angiogenesis *in vitro*. Stem Cells Dev. (2015) 24:1635–47. 10.1089/scd.2014.0316 25867197 PMC4499790

[300] Zhang J Guan J Niu X Hu G Guo S Li Q. Exosomes released from human induced pluripotent stem cells-derived MSCs facilitate cutaneous wound healing by promoting collagen synthesis and angiogenesis. J Transl Med. (2015) 13:49. 10.1186/s12967-015-0417-0 25638205 PMC4371881

[301] Ferguson SW Wang J Lee CJ Liu M Neelamegham S Canty JM. The microRNA regulatory landscape of MSC-derived exosomes: a systems view. Sci Rep. (2018) 8:1419. 10.1038/s41598-018-19581-x 29362496 PMC5780426

[302] Reis M Mavin E Nicholson L Green K Dickinson AM Wang X. Mesenchymal stromal cell-derived extracellular vesicles attenuate dendritic cell maturation and function. Front Immunol. (2018) 9:2538. 10.3389/fimmu.2018.02538 30473695 PMC6237916

[303] Fang S Xu C Zhang Y Xue C Yang C Bi H. Umbilical cord-derived mesenchymal stem cell-derived exosomal MicroRNAs suppress myofibroblast differentiation by inhibiting the transforming growth factor-β/SMAD2 pathway during wound healing. Stem Cell Transl Med. (2016) 5:1425–39. 10.5966/sctm.2015-0367 27388239 PMC5031180

[304] Qiu G Zheng G Ge M Wang J Huang R Shu Q. Mesenchymal stem cell-derived extracellular vesicles affect disease outcomes via transfer of microRNAs. Stem Cell Res Ther. (2018) 9:320. 10.1186/s13287-018-1069-9 30463593 PMC6249826

[305] Baglio SR Rooijers K Koppers-Lalic D Verweij FJ Lanzón MP Zini N. Human bone marrow- and adipose-mesenchymal stem cells secrete exosomes enriched in distinctive miRNA and tRNA species. Stem Cell Res Ther. (2015) 6:127. 10.1186/s13287-015-0116-z 26129847 PMC4529699

[306] Whittaker TE Nagelkerke A Nele V Kauscher U Stevens MM. Experimental artefacts can lead to misattribution of bioactivity from soluble mesenchymal stem cell paracrine factors to extracellular vesicles. J Extracell Vesicles. (2020) 9:1807674. 10.1080/20013078.2020.1807674 32944192 PMC7480412

[307] Blanc KL Davies LC. Mesenchymal stromal cells and the innate immune response. Immunol Lett. (2015) 168:140–6. 10.1016/j.imlet.2015.05.004 25982165

[308] Wang S Zhu R Li H Li J Han Q Zhao RC. Mesenchymal stem cells and immune disorders: from basic science to clinical transition. Front Med. (2019) 13:138–51. 10.1007/s11684-018-0627-y 30062557

[309] Wang Y Chen X Cao W Shi Y. Plasticity of mesenchymal stem cells in immunomodulation: pathological and therapeutic implications. Nat Immunol. (2014) 15:1009–16. 10.1038/ni.3002 25329189

[310] Kronsteiner B Wolbank S Peterbauer A Hackl C Redl H van Griensven M. Human mesenchymal stem cells from adipose tissue and amnion influence T-cells depending on stimulation method and presence of other immune cells. Stem Cells Dev. (2011) 20:2115–26. 10.1089/scd.2011.0031 21381973

[311] Bárcia RN Santos JM Filipe M Teixeira M Martins JP Almeida J. What makes umbilical cord tissue-derived mesenchymal stromal cells superior immunomodulators when compared to bone marrow derived mesenchymal stromal cells? Stem Cells Int. (2015) 2015:1–14. 10.1155/2015/583984 26064137 PMC4443932

[312] Ménard C Dulong J Roulois D Hébraud B Verdière L Pangault C. Integrated transcriptomic, phenotypic, and functional study reveals tissue-specific immune properties of mesenchymal stromal cells: MSC properties rely on their tissue of origin. Stem Cells. (2019) 38:146–59. 10.1002/stem.3077 31502731

[313] Paladino FV Rodrigues J de M da Silva A Goldberg AC. The immunomodulatory potential of Wharton's Jelly Mesenchymal Stem/Stromal Cells. Stem Cells Int. (2019) 2019:1–7. 10.1155/2019/3548917 31281372 PMC6594275

[314] Tago Y Kobayashi C Ogura M Wada J Yamaguchi S Yamaguchi T. Human amnion-derived mesenchymal stem cells attenuate xenogeneic graft-versus-host disease by preventing T cell activation and proliferation. Sci Rep. (2021) 11:2406. 10.1038/s41598-021-81916-y 33510297 PMC7843654

[315] Li M Soder R Abhyankar S Abdelhakim H Braun MW Trinidad CV. WJMSC-derived small extracellular vesicle enhance T cell suppression through PD-L1. J Extracell Vesicles. (2021) 10:e12067. 10.1002/jev2.12067 33598108 PMC7869022

[316] Mattar P Bieback K. Comparing the immunomodulatory properties of bone marrow, adipose tissue, and birth-associated tissue mesenchymal stromal cells. Front Immunol. (2015) 6:560. 10.3389/fimmu.2015.00560 26579133 PMC4630659

[317] Iglesias-Lopez C Obach M Vallano A Agustí A. Comparison of regulatory pathways for the approval of advanced therapies in the European Union and the United States. Cytotherapy. (2021) 23:261–74. 10.1016/j.jcyt.2020.11.008 33483292

[318] Sakai D Schol J Foldager CB Sato M Watanabe M. Regenerative technologies to bed side: evolving the regulatory framework. J Orthop Transl. (2017) 9:1–7. 10.1016/j.jot.2017.02.001 29662794 PMC5822969

[319] Iglesias-López C Agustí A Obach M Vallano A. Regulatory framework for advanced therapy medicinal products in Europe and United States. Front Pharmacol. (2019) 10:921. 10.3389/fphar.2019.00921 31543814 PMC6728416

[320] Jayaraman P Lim R Ng J Vemuri MC. Acceleration of translational mesenchymal stromal cell therapy through consistent quality GMP manufacturing. Front Cell Dev Biol. (2021) 9:648472. 10.3389/fcell.2021.648472 33928083 PMC8076909

[321] James D. How short-term gain can lead to long-term pain. Cell Gene Ther Insights. (2017) 3:18. 10.18609/cgti.2017.018

[322] Sensebé L Bourin P Tarte K. Good manufacturing practices production of mesenchymal stem/stromal cells. Hum Gene Therap. (2011) 22:19–26. 10.1089/hum.2010.197 21028982

[323] Mastrolia I Foppiani EM Murgia A Candini O Samarelli AV Grisendi G. Challenges in clinical development of mesenchymal stromal/stem cells: concise review. Stem Cells Transl Med. (2019) 8:1135–48. 10.1002/sctm.19-0044 31313507 PMC6811694

[324] Sciezyńska A Soszyńska M Szpak P Krześniak N Malejczyk J Kalaszczyńska I. Influence of hypothermic storage fluids on mesenchymal stem cell stability: a comprehensive review and personal experience. Cells. (2021) 10:1043. 10.3390/cells10051043 33925059 PMC8146384

[325] Stolzing A Jones E McGonagle D Scutt A. Age-related changes in human bone marrow-derived mesenchymal stem cells: consequences for cell therapies. Mechan Ageing Dev. (2008) 129:163–73. 10.1016/j.mad.2007.12.002 18241911

[326] Payne KA Didiano DM Chu CR. Donor sex and age influence the chondrogenic potential of human femoral bone marrow stem cells. Osteoarthritis and cartilage OARS. Osteoarthritis Res Society. (2010) 18:705–13. 10.1016/j.joca.2010.01.011 20171308 PMC2862807

[327] Sammour I Somashekar S Huang J Batlahally S Breton M Valasaki K. The Effect of Gender on Mesenchymal Stem Cell (MSC) efficacy in neonatal hyperoxia-induced lung injury. PLoS ONE. (2016) 11:e0164269. 10.1371/journal.pone.0164269 27711256 PMC5053475

[328] Tajiri N Duncan K Borlongan M Pabon M Acosta S de la Pena I. Adult stem cell transplantation: is gender a factor in stemness? Int J Mol Sci. (2014) 15:15225–43. 10.3390/ijms150915225 25170809 PMC4200754

[329] Bernardo ME Locatelli F Fibbe WE. Mesenchymal stromal cells. Ann Ny Acad Sci. (2009) 1176:101–7. 10.1111/j.1749-6632.2009.04607.x 19796238

[330] Fraser JK Wulur I Alfonso Z Hedrick MH. Fat tissue: an underappreciated source of stem cells for biotechnology. Trends Biotechnol. (2006) 24:150–4. 10.1016/j.tibtech.2006.01.010 16488036

[331] Griffin MD Ryan AE Alagesan S Lohan P Treacy O Ritter T. Anti-donor immune responses elicited by allogeneic mesenchymal stem cells: what have we learned so far? Immunol Cell Biol. (2013) 91:40–51. 10.1038/icb.2012.67 23207278

[332] Kim DS Lee MW Lee T-H Sung KW Koo HH Yoo KH. Cell culture density affects the stemness gene expression of adipose tissue-derived mesenchymal stem cells. Biomed Rep. (2017) 6:300–6. 10.3892/br.2017.845 28451390 PMC5403436

[333] Banfi A Muraglia A Dozin B Mastrogiacomo M Cancedda R Quarto R. Proliferation kinetics and differentiation potential of ex vivo expanded human bone marrow stromal cells Implications for their use in cell therapy. Exp Hematol. (2000) 28:707–15. 10.1016/s0301-472x(00)00160-0 10880757

[334] Yang Y-HK Ogando CR See CW Chang T-Y Barabino GA. Changes in phenotype and differentiation potential of human mesenchymal stem cells aging *in vitro*. Stem Cell Res Ther. (2018) 9:131. 10.1186/s13287-018-0876-3 29751774 PMC5948736

[335] LI X-Y Ding J Zheng Z-H Li X-Y Wu Z-B Zhu P. Long-term culture *in vitro* impairs the immunosuppressive activity of mesenchymal stem cells on T cells. Mol Med Rep. (2012) 6:1183–9. 10.3892/mmr.2012.1039 22923041

[336] Wang Y Zhang Z Chi Y Zhang Q Xu F Yang Z. Long-term cultured mesenchymal stem cells frequently develop genomic mutations but do not undergo malignant transformation. Cell Death Dis. (2013) 4:e950. 10.1038/cddis.2013.480 24309937 PMC3877551

[337] Zhao Q Zhang L Wei Y Yu H Zou L Huo J. Systematic comparison of hUC-MSCs at various passages reveals the variations of signatures and therapeutic effect on acute graft-versus-host disease. Stem Cell Res Ther. (2019) 10:354. 10.1186/s13287-019-1478-4 31779707 PMC6883552

[338] Neuhuber B Swanger SA Howard L Mackay A Fischer I. Effects of plating density and culture time on bone marrow stromal cell characteristics. Exp Hematol. (2008) 36:1176–1185. 10.1016/j.exphem.2008.03.019 18495329 PMC2603339

[339] Bartmann C Rohde E Schallmoser K Pürstner P Lanzer G Linkesch W. Two steps to functional mesenchymal stromal cells for clinical application. Transfusion. (2007) 47:1426–35. 10.1111/j.1537-2995.2007.01219.x 17655587

[340] Both SK van der Muijsenberg AJC van Blitterswijk CA de Boer J de Bruijn JD. A Rapid and efficient method for expansion of human mesenchymal stem cells. Tissue Eng. (2007) 13:3–9. 10.1089/ten.2005.0513 17518576

[341] Sotiropoulou PA Perez SA Salagianni M Baxevanis CN Papamichail M. Characterization of the optimal culture conditions for clinical scale production of human mesenchymal stem cells. Stem Cells. (2006) 24:462–71. 10.1634/stemcells.2004-0331 16109759

[342] Sekiya I Larson BL Smith JR Pochampally R Cui J Prockop DJ. Expansion of human adult stem cells from bone marrow stroma: conditions that maximize the yields of early progenitors and evaluate their quality. Stem Cells. (2002) 20:530–41. 10.1634/stemcells.20-6-530 12456961

[343] Hoffmann A Floerkemeier T Melzer C Hass R. Comparison of in vitro-cultivation of human mesenchymal stroma/stem cells derived from bone marrow and umbilical cord. J Tissue Eng Regen M. (2017) 11:2565–81. 10.1002/term.2153 27125777

[344] Sensebé L Bourin P Douay L. Good Manufacturing Practices: Clinical-Scale Production of Mesenchymal Stem Cells. Wiley-VCH Verlag GmbH & Co. KGaA. (2006) 10.1002/3527608745.ch6

[345] Bowles AC Kouroupis D Willman MA Orfei CP Agarwal A Correa D. Signature quality attributes of CD146+ mesenchymal stem/stromal cells correlate with high therapeutic and secretory potency. Stem Cells. (2020) 38:1034–49. 10.1002/stem.3196 32379908

[346] Ilas DC Baboolal TG Churchman SM Jones WG Giannoudis PV Bühring H-J. The osteogenic commitment of CD271+CD56+ bone marrow stromal cells (BMSCs) in osteoarthritic femoral head bone. Sci Rep-uk. (2020) 10:11145. 10.1038/s41598-020-67998-0 32636407 PMC7341749

[347] Cuthbert RJ Giannoudis PV Wang XN Nicholson L Pawson D Lubenko A. Examining the feasibility of clinical grade CD271+ enrichment of mesenchymal stromal cells for bone regeneration. PLoS ONE. (2015) 10:e0117855. 10.1371/journal.pone.0117855 25760857 PMC4356586

[348] Psaltis PJ Paton S See F Arthur A Martin S Itescu S. Enrichment for STRO-1 expression enhances the cardiovascular paracrine activity of human bone marrow-derived mesenchymal cell populations. J Cell Physiol. (2010) 223:530–40. 10.1002/jcp.22081 20162565

[349] Masterson C Devaney J Horie S O'Flynn L Deedigan L Elliman S. Syndecan-2–positive, bone marrow–derived human mesenchymal stromal cells attenuate bacterial-induced acute lung injury and enhance resolution of ventilator-induced lung injury in rats. Anesthesiology. (2018) 129:502–16. 10.1097/aln.0000000000002327 29979191

[350] von Bahr L Sundberg B Lönnies L Sander B Karbach H Hägglund H. Long-term complications, immunologic effects, and role of passage for outcome in mesenchymal stromal cell therapy. Biol Blood Marrow Treat. (2012) 18:557–64. 10.1016/j.bbmt.2011.07.023 21820393

[351] Mochizuki T Muneta T Sakaguchi Y Nimura A Yokoyama A Koga H. Higher chondrogenic potential of fibrous synovium– and adipose synovium–derived cells compared with subcutaneous fat–derived cells: Distinguishing properties of mesenchymal stem cells in humans. Arthritis Rheum. (2006) 54:843–53. 10.1002/art.21651 16508965

[352] Riis S Nielsen FM Pennisi CP Zachar V Fink T. Comparative analysis of media and supplements on initiation and expansion of adipose-derived stem cells. Stem Cell Transl Med. (2016) 5:314–24. 10.5966/sctm.2015-0148 26838270 PMC4807663

[353] Tsutsumi S Shimazu A Miyazaki K Pan H Koike C Yoshida E. Retention of multilineage differentiation potential of mesenchymal cells during proliferation in response to FGF. Biochem Bioph Res Co. (2001) 288:413–9. 10.1006/bbrc.2001.5777 11606058

[354] Solchaga LA Penick K Porter JD Goldberg VM Caplan AI Welter JF. FGF-2 enhances the mitotic and chondrogenic potentials of human adult bone marrow-derived mesenchymal stem cells. J Cell Physiol. (2005) 203:398–409. 10.1002/jcp.20238 15521064

[355] Tekkatte C Gunasingh GP Cherian KM Sankaranarayanan K. “Humanized” stem cell culture techniques: the animal serum controversy. Stem Cells Int. (2011) 2011:504723. 10.4061/2011/504723 21603148 PMC3096451

[356] Burnouf T Strunk D Koh MBC Schallmoser K. Human Platelet Lysate: Replacing Fetal Bovine Serum as a Gold Standard for Human Cell Propagation? (2016). Available online at: https://www.sciencedirect.com/science/article/abs/pii/S0142961215008753?via%3Dihub 10.1016/j.biomaterials.2015.10.065 26561934

[357] Schallmoser K Henschler R Gabriel C Koh MBC Burnouf T. Production and quality requirements of human platelet lysate: a position statement from the working party on cellular therapies of the international society of blood transfusion. Trends Biotechnol. (2020) 38:13–23. 10.1016/j.tibtech.2019.06.002 31326128

[358] Stühler A Blümel J. Specific aspects for virus safety of raw materials for cellular-based medicinal products. Bundesgesundheitsbl. (2015) 58:1233–8. 10.1007/s00103-015-2238-y 26383536

[359] Wu X Kang H Liu X Gao J Zhao K Ma Z. Serum and xeno-free, chemically defined, no-plate-coating-based culture system for mesenchymal stromal cells from the umbilical cord. Cell Proliferat. (2016) 49:579–88. 10.1111/cpr.12279 27492579 PMC6496339

[360] Patrikoski M Juntunen M Boucher S Campbell A Vemuri MC Mannerström B. Development of fully defined xeno-free culture system for the preparation and propagation of cell therapy-compliant human adipose stem cells. Stem Cell Res Therap. (2013) 4:1–5. 10.1186/scrt175 23497764 PMC3707027

[361] Bhat S Viswanathan P Chandanala S Prasanna SJ Seetharam RN. Expansion and characterization of bone marrow derived human mesenchymal stromal cells in serum-free conditions. Sci Rep. (2021) 11:3403. 10.1038/s41598-021-83088-1 33564114 PMC7873235

[362] Nikolits I Nebel S Egger D Kreß S Kasper C. Towards physiologic culture approaches to improve standard cultivation of mesenchymal stem cells. Cells. (2021) 10:886. 10.3390/cells10040886 33924517 PMC8069108

[363] Gottipamula S Muttigi MS Chaansa S Ashwin KM Priya N Kolkundkar U. Large-scale expansion of pre-isolated bone marrow mesenchymal stromal cells in serum-free conditions. J Tissue Eng Regen M. (2013) 10:108–19. 10.1002/term.1713 23495227

[364] Mizukami A Chilima TDP Orellana MD Neto MA Covas DT Farid SS. Technologies for large-scale umbilical cord-derived MSC expansion: experimental performance and cost of goods analysis. Biochem Eng J. (2018) 135:36–48. 10.1016/j.bej.2018.02.018

[365] Koh B Sulaiman N Fauzi MB Law JX Ng MH Idrus RBH. Three dimensional microcarrier system in mesenchymal stem cell culture: a systematic review. Cell Biosci. (2020) 10:75. 10.1186/s13578-020-00438-8 32518618 PMC7271456

[366] Schirmaier C Jossen V Kaiser SC Jüngerkes F Brill S Safavi-Nab A. Scale-up of adipose tissue-derived mesenchymal stem cell production in stirred single-use bioreactors under low-serum conditions. Eng Life Sci. (2017) 14:292–303. 10.1002/elsc.201300134

[367] Timmins NE Kiel M Günther M Heazlewood C Doran MR Brooke G. Closed system isolation and scalable expansion of human placental mesenchymal stem cells. Biotechnol Bioeng. (2012) 109:1817–26. 10.1002/bit.24425 22249999

[368] Mizukami A Orellana MD Caruso SR Prata KL Covas DT Swiech K. Efficient expansion of mesenchymal stromal cells in a disposable fixed bed culture system. Biotechnol Progr. (2013) 29:568–72. 10.1002/btpr.1707 23420706

[369] Hanley PJ Mei Z Durett AG Cabreira-Harrison M da G Klis M Li W. Efficient manufacturing of therapeutic mesenchymal stromal cells using the quantum cell expansion system. Cytotherapy. (2014) 16:1048–58. 10.1016/j.jcyt.2014.01.417 24726657 PMC4087082

[370] Lechanteur C. Large-scale clinical expansion of mesenchymal stem cells in the gmp-compliant, closed automated quantum® cell expansion system: comparison with expansion in traditional T-flasks. J Stem Cell Res Therap. (2014) 4:222. 10.4172/2157-7633.1000222

[371] Stephenson M Grayson W. Recent advances in bioreactors for cell-based therapies. F1000Research. (2018) 7:1. 10.12688/f1000research.12533.1 29770207 PMC5931275

